# Cell cycle dependent methylation of Dam1 contributes to kinetochore integrity and faithful chromosome segregation

**DOI:** 10.1371/journal.pgen.1011760

**Published:** 2025-06-16

**Authors:** Prashant K. Mishra, Wei-Chun Au, John S. Choy, Pedro G. Castineira, Afsa Khawar, Chloé Tessier, Sudipto Das, Andresson Thorkell, Peter H. Thorpe, Elaine Yeh, Kerry S. Bloom, Munira A. Basrai

**Affiliations:** 1 Genetics Branch, National Cancer Institute, National Institutes of Health, Bethesda, Maryland, United States of America; 2 The Catholic University of America, Washington DC, United States of America; 3 School of Biological and Behavioural Sciences, Queen Mary University of London, London, United Kingdom; 4 Protein Characterization Laboratory, Cancer Research Technology Program, Frederick National Laboratory for Cancer Research, Frederick, Maryland, United States of America; 5 University of North Carolina, Chapel Hill, North Carolina, United States of America; Oregon State University, UNITED STATES OF AMERICA

## Abstract

The kinetochore, a megadalton structure composed of centromeric (*CEN*) DNA and protein complexes, is required for faithful chromosome segregation in eukaryotes. The evolutionarily conserved Dam1/DASH complex (Ska1 in metazoans) is one of the essential protein sub-complexes of the budding yeast kinetochore. Previous studies showed that methylation of lysine residue 233 in Dam1 by Set1 is important for haploid growth as mutation of lysine 233 to alanine results in lethality. In this study, we report that Set1-mediated cell cycle dependent Dam1 lysine methylation contributes to kinetochore assembly and chromosomal stability. Our results show that Dam1 methylation is cell cycle regulated with the highest levels of methylation in metaphase. Consistent with these results, co-immunoprecipitation experiments revealed an interaction between Dam1 with Set1 in metaphase cells. Set1 has been shown to colocalize with Jhd2, a histone lysine demethylase which demethylates Set1-methylated histones. Affinity purification-based mass spectroscopy of Jhd2 associated proteins identified seven of the ten subunits of the Dam1 complex; an association of Jhd2 with non-histone proteins, such as Dam1 has not been previously reported. We confirmed the interaction of Jhd2 with Dam1 and showed that cells overexpressing *JHD2* exhibit reduced levels of methylated lysine in Dam1 in wild type and *UBP8* deletion strains, growth defects in kinetochore mutants, reduced levels of kinetochore proteins at *CEN* chromatin, defects in kinetochore biorientation and chromosome missegregation. In summary, we have shown that cell cycle dependent methylation of Dam1 plays a crucial role in the maintenance of kinetochore assembly for faithful chromosome segregation.

## Author summary

Faithful chromosome segregation in eukaryotes is regulated by the kinetochore, a megadalton structure composed of centromeric DNA and associated proteins. Errors in chromosome segregation during the cell cycle result in chromosomal instability that is associated with human diseases, such as cancer and birth defects. Hence, the mechanisms and processes underlying kinetochore function and high-fidelity chromosome segregation are of utmost importance. The epigenetic modifications of evolutionarily conserved kinetochore protein Dam1 play an important role in kinetochore function and chromosome segregation. Phosphorylation of Dam1 by Aurora kinase Ipl1 modulates kinetochore-microtubule interaction, chromosome biorientation and segregation. However, the role of cell cycle mediated lysine methylation of Dam1 is not known. In this study, we showed that lysine methylation of Dam1 is cell cycle regulated with maximum enrichment in mitotic cells. We established an interaction of the histone lysine demethylase, Jhd2 with Dam1 and showed that cells with inducible expression of Jhd2 show defects in Dam1 methylation and kinetochore integrity with chromosome missegregation. In summary, we have defined a novel role for cell cycle dependent methylation of Dam1 in ensuring proper kinetochore assembly and chromosome segregation.

## Introduction

The kinetochore is a multiprotein structure that assembles on centromeric (*CEN*) chromatin, and is marked epigenetically by a specialized histone H3 variant, Cse4 (CENP-A in humans) [1–9]. The structural and functional fidelity of kinetochore is essential for faithful chromosome segregation as its dysfunction results in chromosomal instability (CIN) and aneuploidy [1,6,7,10], a hallmark of many cancer cells [11,12]. Budding yeast contains point centromeres defined by unique *CEN* DNA sequences (~150 bp in size) and recruits a single Cse4-containing nucleosome that is sufficient for the assembly of a functional kinetochore [1,13,14]. In contrast, other eukaryotes have large regional *CEN* regions (4 kb-10 Mb in size) interspersed by CENP-A and canonical histone H3 nucleosomes that act as the platform for the assembly of several kinetochore protein sub-complexes, which facilitate the interactions of *CEN* chromatin with the spindle for faithful chromosome segregation [2–7]. Despite the differences in the centromere size and *CEN* DNA sequence compositions, kinetochore architecture and protein sub-complexes are evolutionarily conserved between budding yeast and other eukaryotes [1–7,15]. The kinetochore is architecturally divided into three components: inner, middle, and the outer kinetochore [16–18]. Inner kinetochore includes proteins such as evolutionarily conserved Cse4, Mif2 (CENP-C in humans), the middle kinetochore is composed of proteins such as Ctf19 (CENP-P in humans), and the outer kinetochore proteins include Dam1/DASH complex (Ska1 complex in humans) among others [16–18].

Several studies have defined the role of post-translational modifications (PTMs) of kinetochore proteins in chromosome segregation [19–28]. In budding yeast, for example, phosphorylation of Cse4 by protein kinases, such as Cdc5, Cdc7, Ipl1 are important for kinetochore assembly [29,30], cell cycle progression [31], and chromosome biorientation [32,33]. We recently reported that cell cycle dependent arginine methylation of Cse4 regulates chromosome segregation [24,34] and other studies showed that lysine methylation in Cse4 facilitates the assembly of Ndc80 complex at the kinetochore [25]. In addition, ubiquitination and sumoylation of Cse4 are shown to be crucial for the maintenance of cellular levels of Cse4 to prevent its mislocalization to non-*CEN* regions for chromosomal stability [35–42]. Likewise, PTMs of CENP-A in humans have also been reported to regulate kinetochore integrity and cell survival [20,21,43]. Moreover, identification and characterization of the role of PTMs of other kinetochore proteins from budding yeast, such as Ndc10, Ask1, Mif2, Dsn1 and from human cells, such as CENP-B, CENP-C, CENP-E, HJURP are also an active area of investigation [44,45].

Dam1, a component of the evolutionarily conserved Dam1/DASH complex of 10 protein subunits, oligomerizes into a ring-like structure around the microtubules, and maintains the integrity of the mitotic spindle during the cell cycle [46–51]. PTMs, such as phosphorylation and methylation of Dam1 have been identified [52–55]. Ipl1-mediated phosphorylation of Dam1 regulates kinetochore-microtubule interactions and chromosome segregation [26,49,51,53,54,56,57]. In addition to phosphorylation, Dam1 is also methylated at lysine residue (Dam1-K233) by histone lysine methyltransferase Set1 and methylation of Dam1 is physiologically significant as Dam1-K233A mutant strains are not viable [55]. Although cell cycle regulated phosphorylation of Dam1 has been extensively investigated [49,51,53,54,56–58], studies to date have not examined if the lysine methylation of Dam1 (^MeK^Dam1) is regulated by the cell cycle and whether cell cycle dependent dynamic methylation of Dam1 affects the structural and functional integrity of the kinetochore and chromosome segregation.

In this study, we show that cell cycle dependent ^MeK^Dam1, with a maximum enrichment in metaphase, is important for kinetochore function and faithful chromosome segregation. Interactome analysis showed an interaction of histone lysine demethylase Jhd2 with seven of the ten subunits of the Dam1 complex. We found that cells overexpressing *JHD2* show reduced levels of ^MeK^Dam1, synthetic growth defects with kinetochore mutants, reduced levels of kinetochore proteins at *CEN* chromatin, defects in kinetochore biorientation, and chromosomal instability. In summary, we have defined a novel role for cell cycle mediated ^MeK^Dam1 in kinetochore function and have showed that dynamic methylation of Dam1 contributes to faithful chromosome segregation.

## Results

### Methylation of Dam1 (^MeK^Dam1) is cell cycle regulated

**Fig 1:**
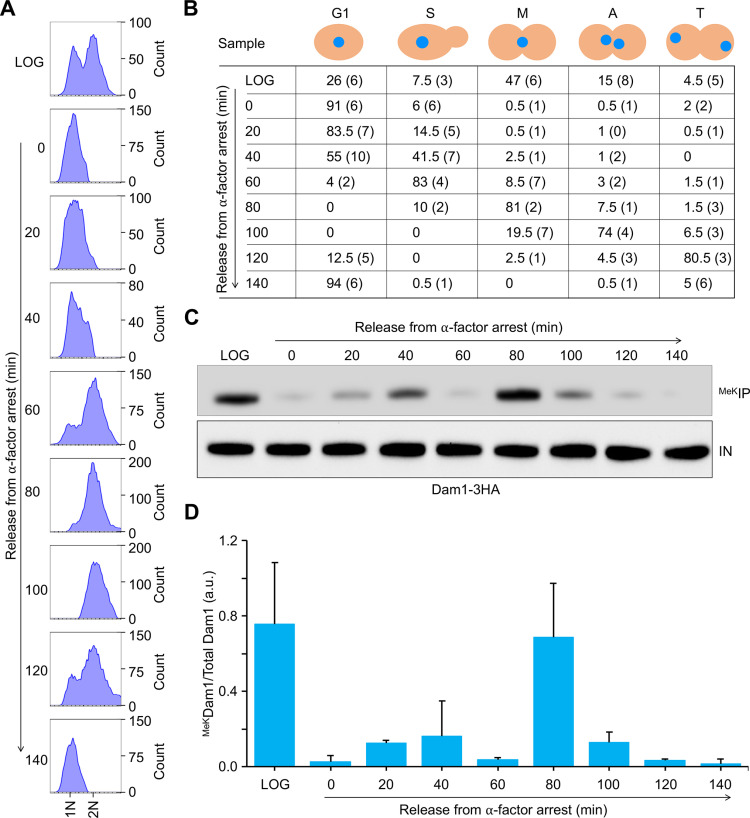
Methylation of Dam1 (^MeK^Dam1) is cell cycle regulated. **(A)** Flow cytometry analysis showing DNA content and cell cycle progression. Wild-type strain (strain# ZK1-5) with Dam1-3HA expressed from its endogenous promoter was grown in YPD to LOG phase at 25°C, synchronized in G1 with α-factor, washed and released into pheromone free media. α-factor was readded at 100 min time point to block cells in the next G1. Samples were taken at time points (min) after release from G1. **(B)** Bud morphology analysis show synchronization in G1 and release into the cell cycle. Cell cycle stage of samples from (A) were determined based on nuclear position and bud morphology by microscopic examination of at least 100 cells for each sample. Different stages of the cell cycle: G1, S-phase **(S)**, metaphase **(M)**, anaphase **(A)**, and telophase **(T)**. Average ± range (parenthesis) from two independent biological replicates is shown. **(C)** The levels of ^MeK^Dam1 are higher in metaphase cells. Methylated proteins were enriched by immunoprecipitation with anti-methyl lysine antibodies using whole cell extracts of wild-type strain from (A) and analyzed by western blotting using α-HA (Dam1-3HA) antibodies as described in *Materials and Methods*. IN, input; ^MeK^IP, immunoprecipitated samples. **(D)** Relative enrichment of ^MeK^Dam1 through the cell cycle after release from G1. Ratio of ^MeK^Dam1 to the total Dam1 was calculated using a semi-quantitative approach in Image J [117] as described in *Materials and Methods*. Two independent biological replicates were done. Average ±range is shown.

Dam1 is a key component of the budding yeast kinetochore and plays an important role in regulating the architecture of kinetochore during the cell cycle, which is essential for high fidelity chromosome segregation [26,49,50,56,57,59–61]. A previous study reported ^MeK^Dam1 and showed that the lysine residue 233 (Dam1-K233) is methylated by Set1 [55]. Cells with mutation of Dam1-K233 to Dam1-K233A are inviable suggesting that the PTMs of lysine 233 in Dam1 is required for cell survival under normal physiological conditions [55]. Despite these results, the molecular role of ^MeK^Dam1 and its physiological significance through the cell cycle remain unexplored. To facilitate the detection of ^MeK^Dam1, we used a methyl-lysine specific polyclonal antibody (PA5–77770, Thermo Fisher Scientific). The specificity of the antibody for methylated lysine was examined using kinetochore protein HA-Cse4 that shows lysine methylation [25] and its variant HA-Cse4^16KR^ as a negative control, in which all lysines were mutated to arginine [62]. The methylated proteins were immunoprecipitated using the methyl-lysine specific antibody from whole cell extracts and analyzed by western blotting by detection with anti-HA antibodies. The methyl-lysine specific antibody detected methylated Cse4 in wild-type HA-Cse4 strain, but reactivity was not observed in HA-Cse4^16KR^ strain (S1 Fig). These results established that the methyl-lysine specific antibody preferentially recognizes methylated lysine thereby providing an ideal tool for studies on ^MeK^Dam1. To determine whether ^MeK^Dam1 is cell cycle regulated, we assayed lysine methylation of endogenously expressed Dam1–3HA in a wild-type strain. Cells were synchronized in G1 (α-factor treatment) and released into pheromone free media (Fig 1A). Cells were collected at different time points after G1 release. Based on the flow cytometry, nuclear position and bud morphology (Figs 1A, 1B and S2), cells were categorized as G1, S, metaphase, anaphase, and telophase as described previously [29,63,64]. Whole cell extracts were prepared, and proteins methylated at lysine residues were immuno-precipitated using methyl-lysine specific antibody (PA5–77770, Thermo Fisher Scientific). The fraction of ^MeK^Dam1 in immunoprecipitated samples was determined by western blotting using anti-HA antibodies. ^MeK^Dam1 was barely detectable in G1 and S-phase (40–60 min post release from α-factor arrest), higher levels in metaphase cells (80 min post release from α-factor arrest) and reduced to the background levels in anaphase and telophase cells (Figs 1C and S2). We quantified the fraction of ^MeK^Dam1 and normalized this to total Dam1 levels (Dam1–3HA) in each stage of the cell cycle as described in the Materials and Methods. The enrichment of ^MeK^Dam1 was significantly higher in metaphase cells than any other stage of the cell cycle (Fig 1D).

**Fig 2:**
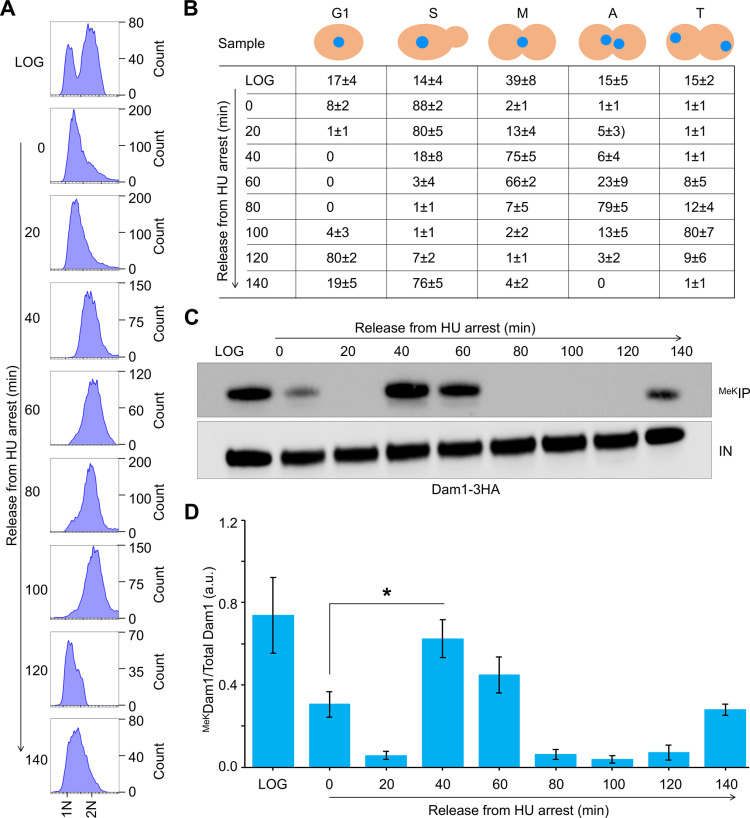
^MeK^Dam1 enrichment observed in metaphase cells. **(A)** Flow cytometry profiles and DNA content analysis. Wild-type strain (strain# ZK5) with Dam1-3HA expressed from its endogenous promoter was grown in YPD to LOG phase at 25°C, synchronized in S-phase with HU, washed and released into YPD media. HU was readded at 100 min time point to block cells in the next S-phase. Samples were collected at time points (min) after release from the S-phase. **(B)** Composition of cells in different cell cycle stage determined based on nuclear position and bud morphology by microscopic examination of at least 100 cells for each sample. Stages of the cell cycle: G1, S-phase **(S)**, metaphase **(M)**, anaphase **(A)**, and telophase **(T)**. Average ± SD from three independent biological replicates is shown. **(C)** ^MeK^Dam1 enrichment is higher in metaphase cells. The levels of ^MeK^Dam1 were determined in samples from (A) by immunoprecipitation with anti-methyl lysine antibodies followed by western blotting with α-HA (Dam1-3HA) antibodies as described in *Materials and Methods*. IN, input; ^MeK^IP, immunoprecipitated samples. **(D)** Relative enrichment of ^MeK^Dam1 after release from the S-phase. ^MeK^Dam1 enrichment was calculated as described in Fig 1D. Three independent biological replicates were done. Average ±SE is shown. *p value <0.05, Student’s *t*-*t*est.

We further confirmed the enrichment pattern of ^MeK^Dam1 in independent cell cycle arrest and release experiments. Cells were synchronized in S-phase (HU treatment) and released into the cell cycle (Fig 2A and 2B). Cell cycle stages were determined based on the flow cytometry profiles, nuclear position and bud morphology (Figs 2A, 2B and S3) following the approach as described previously [29,63,64]. Consistent with results from Fig 1, the highest enrichment of ^MeK^Dam1 was observed in metaphase cells (40–60 min post release from HU arrest), whereas it was barely detectable in anaphase, telophase or G1 cells (80–120 min post release from HU arrest) (Figs 2C, 2D and S3). Notably, slightly enhanced levels of ^MeK^Dam1 were detected in HU-treated cells (0 min and 140 min) (Fig 2C and 2D). Moreover, levels of ^MeK^Dam1 in LOG phase cells was not significantly different from metaphase cells (Figs 1 and 2). Taken together, our results indicate that ^MeK^Dam1 is cell cycle regulated with the highest enrichment of ^MeK^Dam1 observed in metaphase cells.

### Dam1 interacts *in vivo* with Set1 in a cell cycle dependent manner

**Fig 3:**
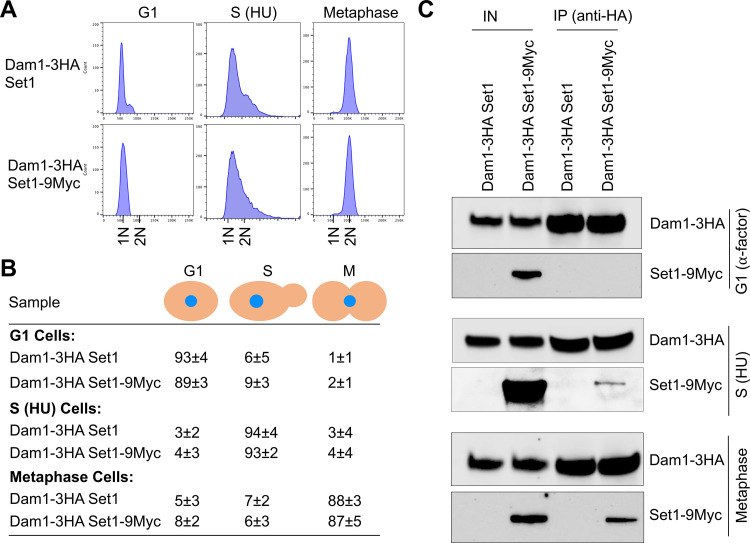
Dam1 interacts *in vivo* with Set1 in a cell cycle dependent manner. **(A)** Flow cytometry analysis showing DNA content. Wild-type strain (strain# 9MYC-SET1-#2) expressing HA-tagged Dam1 and Myc-tagged Set1 (Dam1-3HA Set1-9Myc) and a control strain (strain# ZK1-5) expressing HA-tagged Dam1 (Dam1-3HA Set1) from their native promoters were grown at 25°C to LOG phase, synchronized in G1 with α-factor, S-phase with HU and synchronized in G1 with α-factor, washed and released into pheromone free media for 80 min to capture cells in metaphase. **(B)** Nuclear position and bud morphology analysis show synchronization in different stages of the cell cycle. Cell cycle stage of samples from (A) were determined as described in Fig 2B. Average ± SD from three independent biological replicates for G1, S-phase **(S)**, and metaphase **(M)** is shown. **(C)** *In vivo* interaction of Dam1 with Set1 in G1, S-phase and metaphase cells. IP experiments using whole cell extracts from (A) were performed with α-HA conjugated agarose beads and analyzed by western blotting with α-HA (Dam1), and α-Myc (Set1) antibodies. IN, input; IP, immunoprecipitated samples.

Previous studies have shown an interaction of Dam1 with Set1 in LOG phase cells and a role of Set1 for *in vivo* ^MeK^Dam1 [55]. Based on our results for high levels of ^MeK^Dam1 in metaphase cells (Figs 1 and 2), we hypothesized that the *in vivo* interaction of Dam1 and Set1 may be cell cycle regulated. Co-IP experiments were done using a wild-type strain carrying endogenously expressed Dam1–3HA and Set1–9Myc. Protein extracts from cells synchronized in G1 (α-factor treatment), S-phase (HU treatment) and metaphase (80 min post release from α-factor arrest) were examined for Dam1-Set1 interaction (Fig 3A). The cell cycle synchronization was confirmed by flow cytometry and examination of nuclear and bud morphology (Figs 3A, 3B and S4). IP results showed an *in vivo* interaction between Dam1 and Set1 in metaphase cells (Figs 3C and S4), whereas interaction of Dam1 with Set1 was not detected in G1 cells despite the expression of these two proteins (Fig 3C). Consistent with ^MeK^Dam1 in HU-treated S-phase cells (Fig 2C and 2D), we observed an *in vivo* interaction between Dam1 and Set1 in these cells (Figs 3C and S4). As expected, no signals were detected in control experiments performed with an untagged Set1 strain (Figs 3C and S4). These results suggest that Set1-mediated ^MeK^Dam1 initiates in S-phase and peaks during metaphase. Taken together, we found that cell cycle regulated interaction between Dam1 and Set1 *in vivo* is coincident with the enrichment of ^MeK^Dam1.

### Kinetochore-microtubule interactions contribute to Dam1 methylation

**Fig 4:**
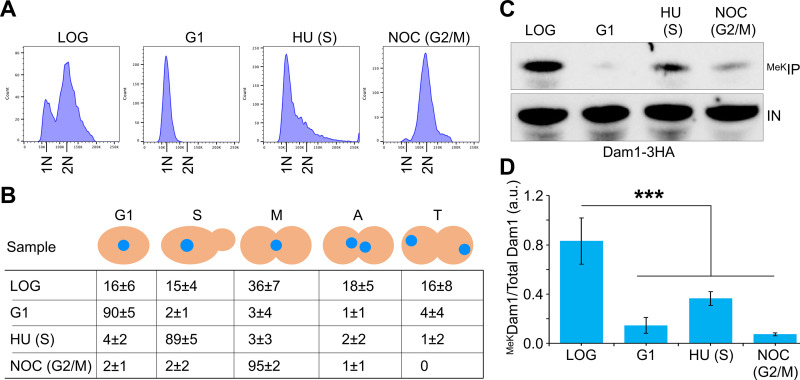
Enrichment of ^MeK^Dam1 requires kinetochore-microtubule interactions. Wild-type strain (strain# ZK5) with Dam1-3HA expressed from its endogenous promoter was grown in YPD to LOG phase at 25°C, synchronized in S-phase with HU, G2/M with nocodazole, and G1 (cells collected after release from G2/M). **(A)** Flow cytometry showing different stages of the cell cycle and DNA content. **(B)** Cell cycle stages of samples from (A) were determined as described in Fig 2B. G1, S-phase **(S)**, metaphase **(M)**, anaphase **(A)**, and telophase **(T)** are shown as average ± SD from three biological replicates. **(C)** The levels of ^MeK^Dam1 are reduced in nocodazole treated G2/M cells. Methylated proteins were enriched by IP with anti-methyl lysine antibodies using whole cell extracts from (A) and analyzed by western blotting using α-HA (Dam1-3HA) antibodies as described in *Materials and Methods*. IN, input; ^MeK^IP, immunoprecipitated samples. **(D)** Relative enrichment of ^MeK^Dam1 in different stages of the cell cycle. ^MeK^Dam1 enrichment was calculated as described in Fig 1D. Three biological replicates were done. Average ±SE is shown. ***p value <0.001, Student’s *t*-*t*est.

Dam1 regulates the dynamics of microtubules polymerization and depolymerization and is required for kinetochores to maintain attachments to microtubules in presence of tension [65–68]. To determine whether kinetochore-microtubule attachments is linked to ^MeK^Dam1, we treated cells with nocodazole that causes depolymerization of microtubules, thereby relieving the tension at the kinetochore [69,70]. As control, we examined the levels of ^MeK^Dam1 in G1, S-phase (HU treatment) and in LOG phase cells (Figs 4A, 4B, and S5). Whole cell extracts were immunoprecipitated using methyl-lysine specific antibody and examined by western blotting using anti-HA antibodies for levels of ^MeK^Dam1. As observed previously (Figs 1 and 2), ^MeK^Dam1 was observed in LOG phase cells, barely detectible in G1, and a slightly higher levels in HU-treated S-phase cells (Fig 4C, 4D and S5). In contrast, no significant enrichment of ^MeK^Dam1 was observed in nocodazole treated cells (Figs 4C, 4D and S5). Notably, reduced association of Dam1 with *CEN* chromatin has been reported in nocodazole treated cells [71]. Taken together, these observations show that kinetochore-microtubule interactions contribute to the enrichment of ^MeK^Dam1.

### Histone lysine demethylase Jhd2 interacts with Dam1 complex proteins *in vivo*

**Fig 5:**
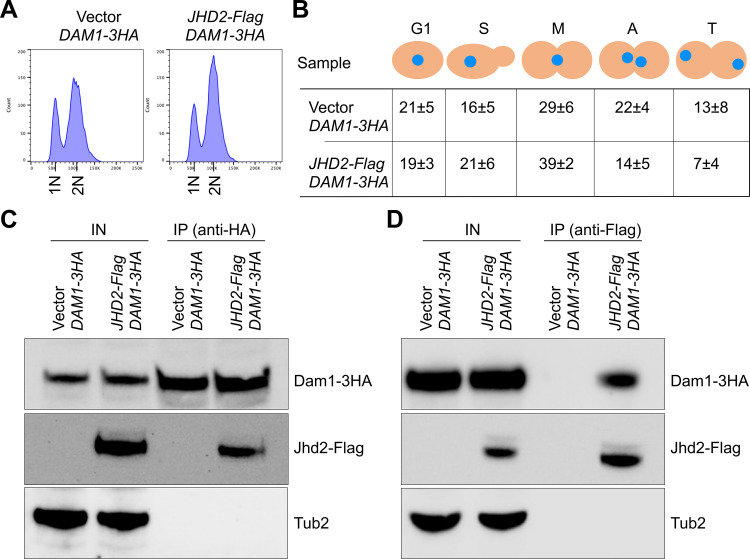
Jhd2 interacts with Dam1 *in vivo.* Wild-type strain expressing HA-tagged Dam1 (Dam1-3HA) from its endogenous promoter carrying *pGAL1*-*URA3*-vector (strain# YMB12579) or *pGAL1-FLAG-JHD2-URA3* (strain# YMB12580) was grown at 25°C to LOG phase in selective media with galactose+raffinose (2% each) for 6 hours. **(A)** Flow cytometry showing DNA content. **(B)** Cell cycle stages of samples from (A) were determined as described in Fig 2B. Cell cycle stages: G1, S-phase **(S)**, metaphase **(M)**, anaphase **(A)**, and telophase **(T)**. Average ± SD from three biological replicates is shown. **(C)** *In vivo* interaction of Jhd2 with Dam1. Whole cell extracts from sample in (A) were prepared and used in IP with α-HA conjugated and α-Flag conjugated agarose beads. Eluted proteins were analyzed by western blotting with α-HA (Dam1), and α-Flag (Jhd2) antibodies. IN, input; IP, immunoprecipitated samples. Tub2 served as a control.

Previous studies have shown that Set1 contributes to ^MeK^Dam1 [55], colocalizes with histone lysine demethylase Jhd2 [72], and that the histone H3-K4 methylated by Set1 is demethylated by Jhd2 [73,74]. Moreover, our results for cell cycle dependent oscillation of ^MeK^Dam1 imply a role for demethylation of Dam1 during the cell cycle. Considering these observations together with a functional relationship between Set1 and Jhd2 [72–74] and *in vivo* interaction between Set1 and Dam1 (Fig 3), we explored the role of Jhd2 in cell cycle dependent oscillation of ^MeK^Dam1. We performed affinity purification-based mass spectroscopy to identify *in vivo* interactors of Jhd2 in a wild-type strain with either *6HIS-HA-JHD2* expressed from the *GAL1* promoter or control strain with empty vector. Affinity purified proteins were analyzed by mass spectrometry and proteins identified in samples from vector containing strain were excluded. In total, 1906 proteins were identified as Jhd2-specific interactors by mass spectrometry, of which 507 and 1396 were encoded by essential and non-essential genes, respectively (S1 Table). Gene Ontology (GO) analysis of these *in vivo* interactors for biological processes was performed using goSlimMapper software from *Saccharomyces* Genome Database (https://www.yeastgenome.org/goSlimMapper). GO analysis of Jhd2-specific interactors identified proteins for kinetochore function, protein methylation, nuclear pore complex, chromosome segregation and spindle pole body (Table 1). Interactome of Jhd2 associated proteins shows an enrichment of proteins for kinetochore function and chromosome segregation such as inner kinetochore (e.g., Cbf1, Cbf2, Cep3, Scm3), outer kinetochore (e.g., Nuf2, Spc24), mitotic exit network (e.g., Nud1, Mob1, Kin4), spindle assembly checkpoint (e.g., Mad1, Mad2), mitotic spindle and microtubule interactions (e.g., Stu2, Slk19, Bni1) (Table 1). Remarkably, we observed an enrichment of Dam1 complex, where seven of ten subunits of the Dam1 complex were identified in kinetochore and chromosome segregation components (Table 1). The *in vivo* interaction of Jhd2 with Dam1 was examined using Co-IP experiments using a strain that expresses HA-tagged Dam1 from its endogenous promoter, and Flag-tagged Jhd2 from the *GAL1* promoter. Strains were grown to LOG phase and protein extracts were prepared for Co-IP and western blot analysis (Figs 5A, 5B and S6). Co-IP experiments performed with Dam1-HA showed Jhd2-Flag; reciprocal pull down with Jhd2-Flag showed Dam1-HA (Figs 5C, 5D and S6). No signals were detected in a control experiment with a vector alone strain (Figs 5C, 5D and S6). Taken together, these results show an *in vivo* interaction between Dam1 and histone lysine demethylase Jhd2.

**Table 1:** Histone lysine demethylase Jhd2 interacts *in vivo* with Dam1 complex. Gene Ontology (GO) based categorization of Jhd2-interactors into functional and structural protein complexes. Listed are the GO categories with protein names, and fraction of proteins from the input over the total number in a given category. Gene names in bold letters represent the component of the Dam1 complex. Statistical significance was determined from the input proteins normalized to the total number of proteins in the yeast genome, and *p* values were calculated based on one sample *t*-test with an expected population mean of 1. Categories with *p* values = < 0.003 are shown.

GO component	Gene Name	Fraction	*p* value
Kinetochore associated proteins	*BIK1 BUB3 CAC2 CBF1 CBF2 CEP3 CSM1* ***DAM1 DAD2 DAD3 DUO1*** *FUN30 HRR25* ***HSK3*** *IRR1 MAD1 MAD2 MCM16 MSI1 NUF2 PSH1 RIF1 RIO1 RLF2 RTS1 SCM3 SGT1 SLK19* ***SPC19 SPC34*** *STU2*	31/77	0.0022
Protein methylation and methyltransferases	*ABD1 BUD23 CDC21 CRG1 COQ5 DIM1 ECM31 EFM1 EMG1 GCD14 HMT1 HSL7 MTQ2 NNT1 OMS1 RKM1 RKM3 RKM4 RMT2 SEE1 SET2 SET5 SPB1 TAE1 TRM1 TRM2 TRM3 TRM5 TRM8 TRM11 TRM44*	31/71	0.0019
Nuclear pore complex	*APQ12 ASM4 LOS1 MEX67 MLP1 NIC96 NSP1 NUP1 NUP2 NUP42 NUP49 NUP53 NUP57 NUP82 NUP100 NUP133 NUP120 NUP145 NUP157 NUP159 NUP170 NUP188 NUP192 PER33 POM152 PSE1 SEH1 SNL1 UIP4*	29/55	0.0013
Chromosome segregation	*AFT1 CBF1 CBF2 CDC14 CHL1 DAD2 DAD3 DAM1 DUO1 GIP4 GLC8 HSK3 IRC15 IRR1 KAR3 MCM16 NUF2 NUP170 SCM3 SFH1 SLK19 SMC3 SMC4 SMC5 SPC19 SPC24 SPC34*	27/79	0.0025
Spindle pole body	*BUB2 CBF2 CDC31 CNM67 DSK2 KAR3 KIC1 KIN4 POM152 SPC19 SPC29 SPC34 SPC42 SPC72 SPC110 STU2 TRM1*	17/36	0.0009

### Jhd2 contributes to demethylation of ^MeK^Dam1 *in vivo*

**Fig 6:**
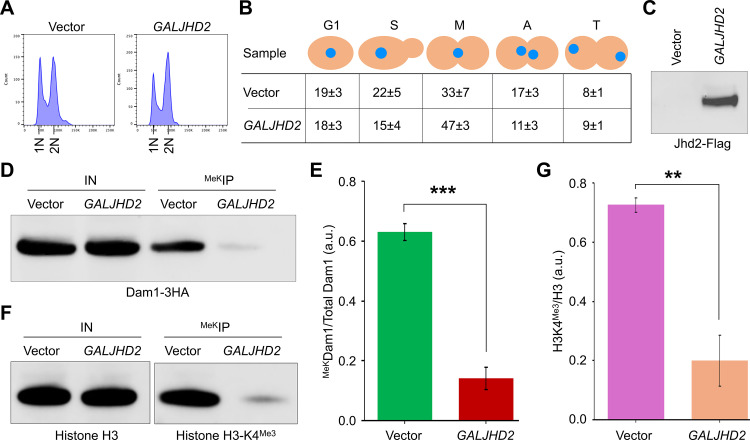
Jhd2 contributes to demethylation of Dam1. Wild-type strain expressing HA-tagged Dam1 (Dam1-3HA) from its endogenous promoter carrying *pGAL1*-*URA3* vector (strain# YMB12579) or *pGAL1-FLAG-JHD2-URA3* (strain# YMB12580) was grown at 25°C to LOG phase in selective media with galactose+raffinose (2% each) for 3 hours. **(A)** Flow cytometry showing DNA content. **(B)** Cell cycle stages of samples from (A) were determined as described in Fig 2B. Different stages are: G1, S-phase **(S)**, metaphase **(M)**, anaphase **(A)**, and telophase **(T)**. Average ± SD from three biological replicates is shown. **(C)** Western blot showing protein levels of Jhd2-Flag expressed from the *GAL1* promoter. **(D)** Overexpression of *JHD2* results in reduction in the levels of ^MeK^Dam1. Methylated proteins were enriched by IP with anti-methyl lysine antibodies using whole cell extracts of samples from (A) and analyzed by western blotting using α-HA (Dam1-3HA) antibodies as described in *Materials and Methods*. IN, input; ^MeK^IP, immunoprecipitated samples. **(E)** Relative enrichment of ^MeK^Dam1 in vector and *GALJHD2* strains. ^MeK^Dam1 enrichment was calculated as described in Fig 1D. Three biological replicates were done. Average ±SE is shown. ***p value <0.001, Student’s *t*-*t*est. **(F)** Overexpression of *JHD2* results in reduction in the levels of histone H3-K4^Me3^. Western blots using samples from (D) were probed with histone H3 and histone H3-K4^Me3^ antibodies. IN, input; ^MeK^IP, immunoprecipitated samples. **(G)** Relative enrichment of histone H3-K4^Me3^ in vector and *GALJHD2* strains. Ratio of histone H3-K4^Me3^ to the total histone H3 was calculated using Image J [117] as described in *Materials and Methods*. Three biological replicates were done. Average ±SE is shown. **p value <0.01, Student’s *t*-tes*t*.

Based on our results showing an *in vivo* interaction of Jhd2 and Dam1, we hypothesized that Jhd2 may regulate demethylation of Dam1. We examined levels of ^MeK^Dam1 using strain with Flag-tagged Jhd2 expressed from the *GAL1* promoter (*GALJHD2*) and HA-tagged Dam1 expressed from its own promoter at its endogenous locus. Protein extracts were prepared after growth of strains in galactose medium (Figs 6A, 6B and S7) and expression of Jhd2 was confirmed by western blotting (Figs 6C and S7). Proteins methylated at lysine residues were immuno-precipitated using a methyl-lysine specific antibody from whole cell extracts and levels of ^MeK^Dam1 were assayed as described earlier (Fig 1). We determined that *GALJHD2* strain showed significantly reduced levels of ^MeK^Dam1 (0.14 ± 0.037, average±standard error) when compared to the vector alone strain (0.63 ± 0.028, *p*-value = 0.0005) (Figs 6D, 6E and S7). As a control, we examined the levels of trimethylation of histone H3-K4 in *GALJHD2* strain. In agreement with previous reports showing demethylation of histone H3-K4 trimethylation by Jhd2 [73,74], the levels of histone H3-K4 trimethylation were reduced significantly in *GALJHD2* strain in comparison to the vector alone strain (Figs 6F, 6G and S7).

**Fig 7:**
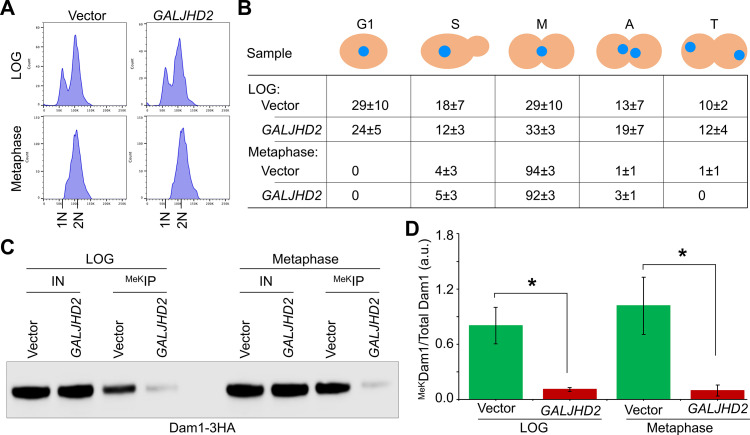
Jhd2 contributes to demethylation of Dam1 in LOG phase and metaphase cells. Wild-type strain expressing HA-tagged Dam1 (Dam1-3HA) from its endogenous promoter carrying *pGAL1*-*URA3* vector (strain# YMB12579) or *pGAL1-FLAG-JHD2-URA3* (strain# YMB12580) was grown at 25°C to LOG phase in selective media with galactose+raffinose (2% each) for 1 hours and then HU was added to synchronize cells in S-phase for 2 hours. Cells were collected, washed and released into selective media with galactose+raffinose (2% each) for 50 min to capture cells in metaphase. **(A)** Flow cytometry analysis showing DNA content of LOG phase and metaphase cells. **(B)** Cell cycle stages of LOG phase and metaphase cells. Cell cycle stage of samples from (A) were determined as described in Fig 2B. Different stages of the cell cycle: G1, S-phase **(S)**, metaphase **(M)**, anaphase **(A)**, and telophase **(T)**. Average ± SD from three biological replicates is shown. **(C)** Overexpression of *JHD2* results in reduced levels of ^MeK^Dam1 in metaphase cells. Methylated proteins were enriched by IP with anti-methyl lysine antibodies using whole cell extracts of samples from (A) and analyzed by western blotting using α-HA (Dam1-3HA) antibodies. IN, input; ^MeK^IP, immunoprecipitated samples. **(D)** Relative enrichment of ^MeK^Dam1 in vector and *GALJHD2* strains. Enrichment was determined as described in Fig 1D. Three biological replicates were done. Average ± SE is shown. *p value <0.05, ***p value <0.001, Student’s *t*-tes*t*.

Since maximum enrichment of ^MeK^Dam1 was observed in metaphase cells (Figs 1 and 2), we examined if overexpression of Jhd2 affects ^MeK^Dam1 in these cells. This was done by using cells synchronized in S-phase (HU treatment), and after release into the cell cycle for 50-min, which represents metaphase cells as determined by flow cytometry, nuclear position and bud morphology assays (Figs 7A, 7B and S8). The western blot analysis showed that the levels of ^MeK^Dam1 were significantly reduced in metaphase cells overexpressing Jhd2 in comparison to the levels observed in the vector strain (Figs 7C, 7D and S8).

We next determined if overexpression of *JHD2* affects the cell cycle regulated enrichment profile of ^MeK^Dam1. We examined the ^MeK^Dam1 pattern in strains carrying vector and compared it with *GALJHD2* strain. Cultures were grown in synthetic media, synchronized in G1 with α-factor and released into pheromone free media. Flow cytometry and bud morphology analyses revealed a delay in mitotic cell cycle progression in *GALJHD2* strains (S9 Fig). The enrichment of ^MeK^Dam1 in vector strain occurs at 120–150 min post G1 release coincident with cells in metaphase, whereas a significant reduction in the level of ^MeK^Dam1 was observed in *GALJHD2* strain throughout the cell cycle (S9 Fig). Taken together, these results indicate that Jhd2 contributes to cell cycle dependent *in vivo* demethylation of Dam1.

### Overexpression of *JHD2* (*GALJHD2*) exhibits growth defects with kinetochore mutants

**Fig 8:**
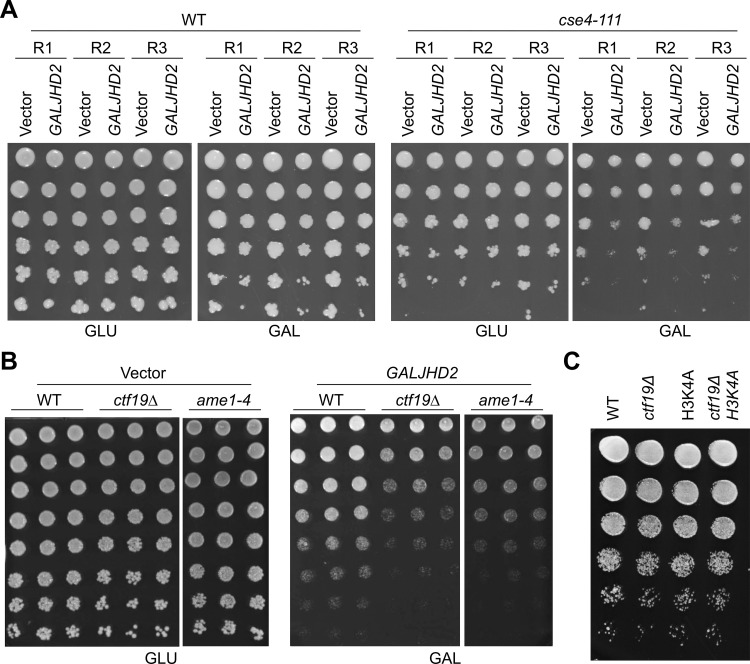
Overexpression of *JHD2* contributes to growth defects in kinetochore mutants. **(A)** Overexpression of *JHD2* contributes to growth defects in *cse4-111* strain. 5 μL of cell suspension (OD_600_ = 1 and its 5-fold dilutions) of *cse4-111* with *pGAL1-URA3* vector (strain# YMB12415) or *pGAL1-6HIS-HA-JHD2-URA3* (strain# YMB12416) and wild type with *pGAL1- URA3* vector (strain# YMB12411) or *pGAL1-6HIS-HA-JHD2-URA3* (strain# YMB12412) were plated on SC-URA with glucose (2%) or galactose+raffinose (2% each) plates and grown at 25°C for 3 days. **(B)** Overexpression of *JHD2* contributes to growth defects in *ctf19Δ and ame1-4* strains. 5 μL of cell suspension (OD_600_ = 1 and its 5-fold dilutions) of *ctf19Δ* with *pGAL1-URA3* vector (strain# YMB12274) or *pGAL1-6HIS-HA-JHD2-URA3* (strain# YMB12248); *ame1-4* with *pGAL1-URA3* vector (strain# YMB12307) or *pGAL1-6HIS-HA-JHD2-URA3* (strain# YMB12308); and wild type with *pGAL1-URA3* vector (strain# YMB12282) or *pGAL1-6HIS-HA-JHD2-URA3* (strain# YMB12281) were plated on SC-URA with glucose (2%) or galactose+raffinose (2% each) plates and grown at 25°C for 3 days. **(C)** Non-methylatable mutant of histone H3 (*H3-K4A*) does not show growth defects when combined with a kinetochore mutant. 5 μL of cell suspension (OD_600_ = 1 and its 5-fold dilutions) of wild-type (strain# WT-12380), *ctf19Δ* (strain# *ctf19Δ*-12381), *H3-K4A* (strain# *H3K4A*-12382), and *H3-K4A ctf19Δ* (strain# *H3K4Actf19Δ* -12383) were plated on YPD plates and grown at 25°C for 3 days.

Our results showing Jhd2-mediated demethylation of Dam1 allowed us to test the physiological significance of ^MeK^Dam1. Since Dam1 is an essential component of budding yeast kinetochore [47,50,52,75–77], we postulated that constitutive demethylation of Dam1 throughout the cell cycle by Jhd2 overexpression (*GALJHD2*) will weaken the kinetochore and affect the growth of kinetochore mutants. Notably, the *CEN* recruitment of Cse4, which is an essential component of *CEN* nucleosome allows for the assembly of kinetochore protein complexes such as, the COMA complex (Ctf19, Okp1, Mcm22, and Ame1) at the kinetochore for faithful chromosome segregation [78–80]. Hence, we examined the growth phenotype of wild-type and kinetochore mutants (e.g., *cse4–111*, *ctf19Δ* and *ame1–4*) upon *JHD2* overexpression. The *cse4–111* carries L194Q mutation that interferes with the interaction of Cse4 and histone H4 and exhibits growth defects at 37°C [81], whereas *ctf19Δ* and *ame1–4* mutants exhibit chromosome segregation, cohesion and cell cycle defects [82,83]. Growth assays showed that *cse4–111*, *ctf19Δ* and *ame1–4* strains exhibit growth defects with *GALJHD2* on galactose plates at the permissive temperature of 25°C (Fig 8A and 8B). To exclude the possibility that demethylation of histone H3-K4 trimethylation upon *JHD2* overexpression may contribute to growth defects observed in kinetochore mutants, we examined the growth phenotype of *ctf19Δ* in combination with a non-methylatable mutant of histone H3 (*H3-K4A*). The growth of *ctf19Δ H3-K4A* mutant was similar to the single mutant (*ctf19Δ* or *H3-K4A*) and that of a wild-type strain (Fig 8C). Taken together, these results show that overexpression of *JHD2* results in growth defects with kinetochore mutants, and this is independent of methylation of histone H3-K4.

A previous study has shown that deletion of *UBP8*, a deubiquitinating enzyme that removes ubiquitin from histone H2B [84], shows increased levels of ^MeK^Dam1 [85]. We hypothesized that the increased ^MeK^Dam1 levels upon *UBP8* deletion may counteract *GALJHD2* activity and this may suppress the growth defects observed in *ctf19∆ GALJHD2* strain. Hence, we examined the effect of *JHD2* overexpression on the growth of wild type, *ubp8Δ*, *ctf19Δ* and *ubp8Δ ctf19Δ* strains. Our results showed that growth defects of *ctf19∆ GALJHD2* were not suppressed by *ubp8∆* (S10 Fig). We reasoned that ^MeK^Dam1 levels may still be reduced due to overexpression of *JHD2* in *ubp8∆* strain. Western blot analysis confirmed that deletion of *UBP8* results in accumulation of ^MeK^Dam1 as reported previously [85]. However, levels of ^MeK^Dam1 were not significantly different between wild type *GALJHD2* and *ubp8∆ GALJHD2* strains (S11 Fig). These results suggest that increased levels of ^MeK^Dam1 in *ubp8∆* strain cannot counteract the effect of *GALJHD2*. Taken together, these results show that overexpression of *JHD2* contributes to growth defects in kinetochore mutants.

### The levels of kinetochore proteins at *CEN* chromatin are reduced in *GALJHD2* strains

**Fig 9:**
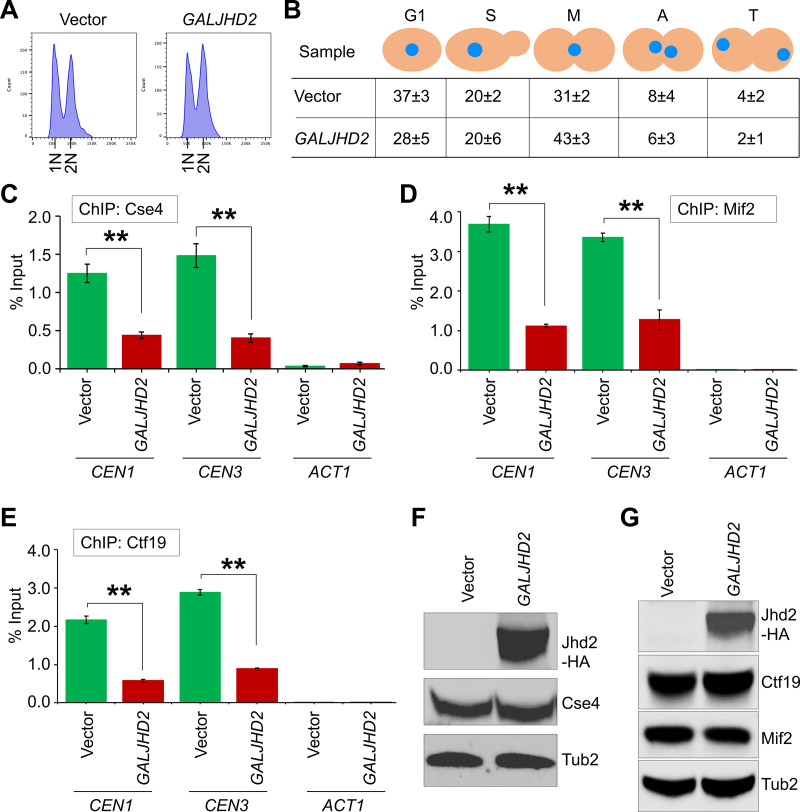
Reduced *CEN* association of kinetochore proteins Cse4, Mif2 and Ctf19 in *GALJHD2* strain. Wild-type strain with endogenously expressed GFP-Cse4 (only copy in the genome) carrying *pGAL1*-*URA3* vector (strain# YMB12364) or *pGAL1-FLAG-JHD2- URA3* (strain# YMB12365) was grown at 25°C to LOG phase in selective media with galactose+raffinose (2% each) for 6 hours. **(A)** Flow cytometry analysis showing DNA content. **(B)** Cell cycle stages of samples from (A) were determined as described in Fig 2B. Cell cycle stages are: G1, S-phase **(S)**, metaphase **(M)**, anaphase **(A)**, and telophase **(T)**. Average ± SD from three biological replicates is shown. **(C)** Levels of Cse4 are reduced at *CEN* chromatin in *GALJHD2* strains. ChIP was performed with α-GFP antibodies using chromatin from same strains as used in (A). Enrichment of Cse4 at *CENs* (*CEN1* and *CEN3*) and a negative control (*ACT1*) was determined by qPCR and is presented as % input. Average from three biological replicates ±SE. **p value <0.01, Student’s *t*-*t*est. **(D)** Levels of Mif2 are reduced at *CEN* chromatin in *GALJHD2* strains. ChIP was performed with α-Mif2 antibodies using chromatin from same strains as in (A). Enrichment of Mif2 at *CENs* (*CEN1* and *CEN3*) and a negative control (*ACT1*) as above. Average from three biological replicates ± SE. **p value <0.01, Student’s *t*-tes*t*. **(E)** Levels of Ctf19 are reduced at *CEN* chromatin in *GALJHD2* strains. ChIP was performed with α-Ctf19 antibodies using chromatin from same strains as in (A). Enrichment of Ctf19 at *CENs* (*CEN1* and *CEN3*) and a negative control (*ACT1*) was determined as above. Average from three biological replicates ± SE. **p value <0.01, Student’s *t*-test. **(F)** Expression of Cse4 in vec*t*or, and *GALJHD2* strains are similar. The western blotting of protein extracts was done using α-GFP (Cse4), α-HA (Jhd2) and α-Tub2 (loading control) antibodies. **(G)** Expression of Mif2 and Ctf19 in vector and *GALJHD2* strains are similar. The western blotting of protein extracts was done using α-Mif2, α-Ctf19, and α-Tub2 (loading control) antibodies.

We reasoned that growth defects due to *GALJHD2* in *cse4–111* and *ctf19∆* strains (Fig 8) may be due to defects in kinetochore that could potentially be exacerbated by reduction in the levels of ^MeK^Dam1 in these strains. Hence, we examined the levels of Cse4, Ctf19 and Cse4 interacting protein Mif2 [86–89] in wild-type strain with vector or *GALJHD2* (Figs 9A, 9B and S12). In agreement with our hypothesis, Chromatin Immunoprecipitation (ChIP)-qPCR showed that the enrichment of Cse4 at *CEN* was reduced significantly in *GALJHD2* strain when compared to the vector alone (Fig 9C). The reduced *CEN* association of Cse4 led us to examine if *CEN* localization of Mif2 is affected in *GALJHD2* strain. The enrichment of Mif2 at *CEN* was reduced significantly in *GALJHD2* strain when compared to the vector alone (Fig 9D). Similarly, *CEN* levels of Ctf19 were also reduced significantly in *GALJHD2* strain than the levels those observed for the vector strain (Fig 9E). No significant differences in *CEN* levels of Ctf19 were observed between wild type and *H3-K4A* strains (S13 Fig). Moreover, no significant enrichment of Cse4, Mif2 or Ctf19 was detected at the non-*CEN ACT1* locus used as a negative control (Figs 9 and S12). The western blot analysis showed similar levels of expression of Cse4, Mif2 and Ctf19 in strains carrying vector or *GALJHD2* (Figs 9F, 9G and S12). We propose that the growth defects for *GALJHD2* in kinetochore mutants (Fig 8) may be due to reduced *CEN* association of kinetochore proteins in *GALJHD2* strain.

### *GALJHD2* strains exhibit declustering of kinetochore and chromosome missegregation

**Fig 10:**
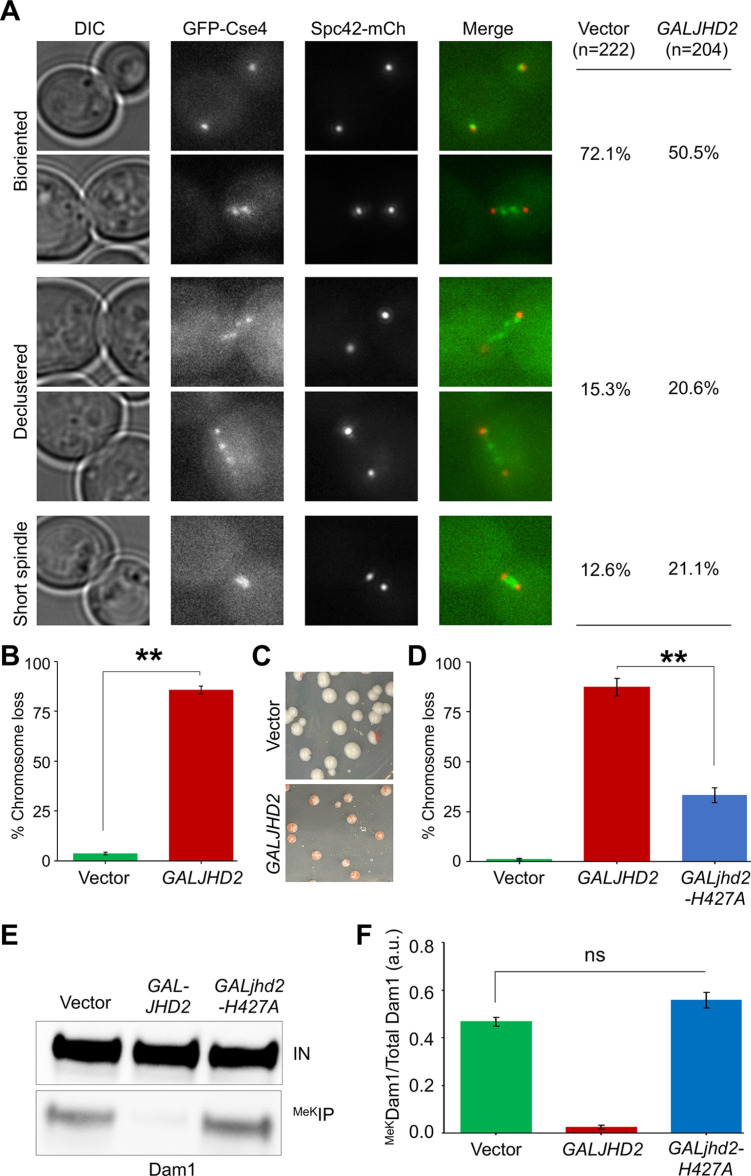
Overexpression of *JHD2* exhibits kinetochore declustering and CIN phenotypes. **(A)** Kinetochore declustering phenotype is increased in *GALJHD2* strain. Wild-type strain (GFP-Cse4 Spc42-mCherry) carrying *pGAL1*-*URA3* vector (strain# YMB12681) or *pGAL1-FLAG-JHD2-URA3* (strain# YMB12682) was grown in selective media with galactose (2%) for 6 hours. Mitotic cells based on the spindle size were examined for kinetochore biorientation, declustering and spindle phenotypes. Percentage of cells in each category are shown. n, number of the cells examined. **(B)** Overexpression of *JHD2* exhibits errors in chromosome segregation. Frequency of CF loss in wild-type with *pGAL1*-*URA3* vector (strain# YMB12467) or *pGAL1-6HIS-HA-JHD2-URA3* (strain# YMB12468) was determined as described in *Materials and Methods*. At least 1000 colonies each from three independent transformants were counted. Average ± SE is shown. **p value < 0.01, Student’s *t t*est. **(C)** Schematic images showing chromosome segregation errors upon overexpression of *JHD2*. **(D)** *GALJHD2-*induced chromosome loss is mediated by its catalytic activity. Frequency of CF loss in wild-type with *pGAL1*-*URA3* vector (strain# YMB12467), *pGAL1-6HIS-HA-JHD2-URA3* (strain# YMB12468) or a catalytically inactive *pGAL1-6HIS-HA-jhd2-H427A-URA3* (strain# YMB12469) was determined as described in *Materials and Methods*. At least 1000 colonies each from three independent transformants were counted. Average ± SE is shown. **p value < 0.01, Student’s *t* tes*t*. **(E)** Jhd2-mediated demethylation of Dam1 is modulated by its catalytic activity. Wild-type strain expressing HA-tagged Dam1 (Dam1-3HA) from its endogenous promoter carrying *pGAL1*-*URA3* vector (strain# YMB12850), *pGAL1-6HIS-HA-JHD2-URA3* (strain# YMB12851) or *pGAL1-6HIS-HA-jhd2-H427A-URA3* (strain# YMB12852) was grown at 25°C to LOG phase in selective media with galactose+raffinose (2% each) for 3 hours. Methylated proteins were enriched by IP with anti-methyl lysine antibodies using whole cell extracts and analyzed by western blotting using α-Dam1 antibodies following procedure as in *Materials and Methods*. IN, input; ^MeK^IP, immunoprecipitated samples. **(F)** Relative enrichment of ^MeK^Dam1 in *GALJHD2* and its catalytic mutant strains. ^MeK^Dam1 enrichment was calculated from the western blots in (E) as described in Fig 1D. Three biological replicates were done. Average ± SE is shown. ns = statistically not significant.

Previous studies have shown that Dam1 complex plays a role in sister kinetochore biorientation [76]. Furthermore, reduced levels of *CEN*-associated Cse4 contributes to defects in spindle biorientation and errors in chromosome segregation [87,90–92]. The reduced levels of *CEN*-associated Cse4, Mif2 and Ctf19 prompted us to examine the structural and functional integrity of kinetochores in *GALJHD2* strains. We monitored the biorientation of centromeres by examining the localization of Spc42-mCherry, a central component of spindle pole and GFP-Cse4 in mitotic cells of wild-type strain with vector or *GALJHD2.* The defects in Cse4 biorientation in *GALJHD2* strain was significantly higher than the vector strain (*p*-value = 2.73x10^-7^, χ^2^-test; Fig 10A). In the vector strain, 72% of the cells had bioriented kinetochores, 13% short spindle, and 15% declustered kinetochores. Whereas, in the *GALJHD2* strain, 50% of the cells had bioriented kinetochores, 21% short spindle, and 21% declustered kinetochores (Fig 10A). The defects observed in *GALJHD2* strains are similar to that observed for mutants defective in kinetochore integrity such as *dam1* mutants [56,93–95]. Our results show that the perturbation of cell cycle dependent ^MeK^Dam1 affects the structural and functional integrity of kinetochores.

We next examined if *GALJHD2* strain exhibits defects in chromosome segregation. This was done by assaying the frequency of loss of a reporter chromosome fragment (CF) in strains with vector or *GALJHD2* using a colony color assay as described previously [96]. The frequency of CF loss in *GALJHD2* strains was 85.6 ± 1.97% (average±SE), which was substantially higher and significantly different from the vector strain (3.7 ± 0.62%; *p*-value = < 0.0001; Fig 10B, 10C and S2 Table). The CF loss observed in *GALJHD2* strains is similar to the CF loss reported for kinetochore mutants, such as *ndc10–1*, *mcm21∆,* and *mad1∆* strains [97,98]. To determine if the increased frequency of CF loss is due to demethylase activity of *JHD2*, we examined CF loss in cells with catalytically inactive mutant of *JHD2* (*jhd2-H427A*) [73,99]. The increased frequency of CF loss was significantly reduced in *GALjhd2-H427A* strains when compared to *GALJHD2* strain (Fig 10D and S2 Table) suggesting that increased frequency of CF loss in *GALJHD2* strains is due to its demethylase activity. We note that the frequency of CF loss in *GALjhd2-H427A* is slightly higher than the vector control, which may be due to chromatin-associated role of Jhd2 independent of its demethylase activity [100]. We next examined the levels of ^MeK^Dam1 in wild type strain carrying catalytically inactive *GALjhd2-H427A, GALJHD2* and vector control. *GALJHD2* strains showed significantly reduced levels of ^MeK^Dam1, whereas no significant reduction in the levels of ^MeK^Dam1 was observed in strains overexpressing *jhd2-H427A* when compared to vector alone strain (Fig 10E, 10F and S14). Based on these results, we conclude that *GALJHD2* contributes to kinetochore dysfunction and chromosome missegregation.

## Discussion

Dam1 is an essential kinetochore protein with an important role in kinetochore-microtubule dynamics and chromosome segregation in budding yeast [26,49,50,56,57,59–61]. In this study, we found that cell cycle dependent ^MeK^Dam1 contributes to proper kinetochore function and faithful chromosome segregation. The enrichment of ^MeK^Dam1 in metaphase cells is dependent on kinetochore-microtubule attachments. Moreover, we provide evidence for a novel role for histone lysine demethylase Jhd2 in demethylation of a non-histone substrate Dam1 *in vivo*. Cells overexpressing *JHD2* (*GALJHD2*) show reduced levels of ^MeK^Dam1 with defects in kinetochore structure-function and errors in chromosome segregation.

Methylation of Dam1 is regulated by the cell cycle with maximum levels of ^MeK^Dam1 observed in metaphase cells. We observed a high degree of coordination between Dam1 methylation and the cell cycle with maximum enrichment of ^MeK^Dam1 in metaphase cells and reduced levels in other stages (anaphase, telophase, or G1) of the cell cycle. Moreover, enrichment of ^MeK^Dam1 in metaphase correlates with *in vivo* interaction of Dam1 with Set1, a previously reported lysine methyltransferase for Dam1 [55,85]. Notably, accumulation of ^MeK^Dam1 in metaphase is dependent on kinetochore-microtubule interactions as an enrichment of ^MeK^Dam1 was not observed in nocodazole treated cells. Our results for reduced levels of ^MeK^Dam1 in late mitotic cells (100 and 120 min; Fig 1) is consistent with previous studies showing that ^MeK^Dam1 antagonizes Ipl1 activity for error correction in these stages of the cell cycle [55].

To investigate the role of dynamic methylation of Dam1, we pursued studies with Jhd2 based on the co-localization of Set1, a lysine methyltransferase for Dam1 [55] with Jhd2, a histone lysine demethylase [72]. Our mass spectrometry analysis of Jhd2 associated proteins identified 1906 proteins including seven of the ten subunits of Dam1 complex. Our results showing an interaction of Dam1 with Jhd2 and reduced ^MeK^Dam1 levels in *GALJHD2* strains support a role for Jhd2 as lysine demethylase for Dam1 *in vivo*. Notably, Dam1 represents one of the first non-histone, kinetochore-associated substrate for Jhd2. Studies to date have reported a role for Jhd2 in demethylation of H3-K4 trimethylation [73,74]. Reduced levels of ^MeK^Dam1 were observed in cells overexpressing *JHD2* (*GALJHD2*) and these cells exhibit: a) synthetic growth defects when combined with kinetochore mutants, (b) reduction in the levels of *CEN*-associated kinetochore proteins Cse4, Mif2, and Ctf19, (c) declustering of the kinetochores, and (d) errors in chromosome segregation. These phenotypes of *GALJHD2* strains may not be entirely due to defects in ^MeK^Dam1 as we cannot rule out the direct or indirect consequences of the other Jhd2-interacting proteins identified in mass spectrometry. Moreover, *GALJHD2* showed reduced levels of ^MeK^Dam1 in *ubp8*∆ strains, which provides specificity for *GALJHD2*-mediated demethylation of ^MeK^Dam1. Using overexpression of *JHD2* as an experimental tool, we have shown that cell cycle dependent, dynamic methylation and/or demethylation of Dam1 is linked with the maintenance of kinetochore integrity and chromosome stability. The growth defects of *GALJHD2* with kinetochore mutants, such as *cse4–111, ctf19∆* and *ame1–4* correlate with the reduced levels of Cse4, Ctf19 and Mif2. We previously reported a crosstalk between Cse4 and Dam1 [32] and other studies have shown reduced levels of *CEN*-associated Cse4 in a *dam1* mutant [87]. Notably, growth defects were not observed when kinetochore mutants were combined with histone *H3-K4A* strain that phenocopies demethylated H3-K4. Furthermore, *CEN* levels of Ctf19 were not reduced in *H3-K4A* strain when compared to the levels in a wild-type strain. These data show that the growth defects and reduced levels of kinetochore proteins at *CEN* in *GALJHD2* strain are not due to effects of H3-K4 demethylation.

Our results showing defects in kinetochore clustering and biorientation in *GALJHD2* strains with reduced levels of ^MeK^Dam1 provide physiological relevance for the role of Dam1 lysine methylation in mitosis. A correlation between reduced levels of *CEN*-associated kinetochore proteins, and kinetochore declustering to CIN has been well documented [101–105]. Consistent with these studies, we observed CIN with increased frequency of chromosome missegregation in *GALJHD2* strain. Moreover, constitutive demethylase activity of Jhd2 (*GALJHD2*) contributes to the CIN phenotypes as significantly reduced levels of chromosome missegregation was observed in strain with catalytically inactive mutant of *JHD2* (*GALjhd2-H427A*). Further support that Jhd2-mediated demethylation of Dam1 and not H3K4 contributes to CIN is based on results showing that histone H3-K4A mutants do not exhibit chromosome missegregation [106]. Remarkably, a role of lysine methylation in chromosomal stability has also been reported in human cells [107,108].

In summary, we have shown that the dynamic cell cycle mediated ^MeK^Dam1 contributes to functional integrity of the kinetochore for faithful chromosome segregation. Moreover, Dam1 complex (Ska complex in humans), Set1 (SETD1A/B) and Jhd2 (KDM5) are evolutionarily conserved between budding yeast and human cells [15,109,110]. Notably, misregulation of the Ska complex, SETD1A/B and KDM5 has been linked to the development and progression of many types of cancers [111–116], however the mechanisms are unknown. Since the molecular functions of these complexes are evolutionarily conserved, our studies from budding yeast offer novel insights into the molecular mechanisms of protein lysine methylation and their physiological significance for chromosome segregation and how defects in these epigenetic pathways contribute to aneuploidy observed in many cancers.

## Materials and methods

### Yeast media, strains, and plasmids

The yeast strains used in this study were grown in yeast extract peptone dextrose (YPD; 1% yeast extract, 2% Bacto-peptone, 2% glucose) or in synthetic minimal medium supplemented with the required carbon source and amino acids drop out based on the plasmid selection. The demethylation and associated experiments were carried out using plasmid-borne *JHD2* expressed from *GAL1* promoter (*pGALJHD2*). All the experiments were performed in three independent biological replicates using identical methodologies. The yeast strains along with their genotypes, and plasmids used in this investigation are listed in Table 2.

**Table 2:** List of strains and plasmids used in this study.

(A) *Saccharomyces cerevisiae* strains:		
Strain	Genotype	Reference
ZK1–5	*MATa ura3–52 his3 dam1Δ::DAM1–3HA*	Sharon Dent, MD Anderson Cancer Center
ZK5	*MATα leu2 ura3–52 his3 dam1Δ::DAM1–3HA*	Sharon Dent, MD Anderson Cancer Center
9MYC-SET1-#2	*MATa dam1Δ::DAM1–3HA set1Δ::9MYC-SET1-LEU2*	Sharon Dent, MD Anderson Cancer Center
YMB12248	*MATa ura3Δ0 leu2Δ0 his3Δ1 met15Δ0 ctf19Δ::KAN pGAL1–6HIS-HA-JHD2-URA3 (pMB2030)*	This study
YMB12274	*MATa ura3Δ0 leu2Δ0 his3Δ1 met15Δ0 ctf19Δ::KAN pGAL1-URA3 Vector (pMB433)*	This study
YMB12281	*MATa ura3Δ0 leu2Δ0 his3Δ1 met15Δ0 pGAL1–6HIS-HA-JHD2-URA3 (pMB2030)*	This study
YMB12282	*MATa ura3Δ0 leu2Δ0 his3Δ1 met15Δ0 pGAL1-URA3 Vector (pMB433)*	This study
YMB12307	*MATa trp1∆63 his3∆200 lys2–801 leu2∆1 ura3–52 ade2–101 ame1–4 pGAL1-URA3 Vector (pMB433)*	This study
YMB12308	*MATa trp1∆63 his3∆200 lys2–801 leu2∆1 ura3–52 ade2–101 ame1–4 pGAL1–6HIS-HA-JHD2-URA3 (pMB2030)*	This study
YMB12364	*MATa trp1Δ63 leu2Δ ura3–52 his3 Δ 200 lys2–8Δ1 GFPCSE4::TRP1 (pKK1) SPC29CFP::KAN pGAL1-URA3 Vector (pMB433)*	This study
YMB12365	*MATa trp1Δ63 leu2Δ ura3–52 his3 Δ 200 lys2–8Δ1 GFPCSE4::TRP1 (pKK1) SPC29CFP::KAN pGAL1–6HIS-HA-JHD2-URA3 (pMB2030)*	This study
YMB12411	*MATa ade2 can1–100 his3,11,15 leu203,112 trp1–1 ura3–1 cse4Δ::HA-CSE4::TRP1-CEN pGAL1-URA3 Vector (pMB433)*	This study
YMB12412	*MATa ade2 can1–100 his3,11,15 leu203,112 trp1–1 ura3–1 cse4Δ::HA-CSE4::TRP1-CEN pGAL1–6HIS-HA-JHD2-URA3 (pMB2030)*	This study
YMB12415	*MATa ade2 can1–100 his3,11,15 leu203,112 trp1–1 ura3–1 cse4Δ::HA-cse4–111::TRP-CEN pGAL1-URA3 Vector (pMB433)*	This study
YMB12416	*MATa ade2 can1–100 his3,11,15 leu203,112 trp1–1 ura3–1 cse4Δ::HA-cse4–111::TRP-CEN pGAL1–6HIS-HA-JHD2-URA3 (pMB2030)*	This study
YMB12467	*MATa ura3–52 lys2–801 ade2–101 trp1∆63 his3∆200 leu2∆1 CFIII (CEN3L.YPH278) HIS3 SUP11 pGAL1-URA3 Vector (pMB433)*	This study
YMB12468	*MATa ura3–52 lys2–801 ade2–101 trp1∆63 his3∆200 leu2∆1 CFIII (CEN3L.YPH278) HIS3 SUP11 pGAL1–6HIS-HA-JHD2-URA3 (pMB2030)*	This study
YMB12469	*MATa ura3–52 lys2–801 ade2–101 trp1∆63 his3∆200 leu2∆1 CFIII (CEN3L.YPH278) HIS3 SUP11 pGAL1–6HIS-HA-jhd2-H427A-URA3 (pMB2153)*	This study
YMB12579	*MATα leu2 ura3–52 his3 dam1Δ::DAM1–3HA pGAL1-URA3 Vector (pMB433)*	This study
YMB12580	*MATα leu2 ura3–52 his3 dam1Δ::DAM1–3HA pGAL1-FLAG-JHD2-URA3 (pMB2163)*	This study
YMB12681	*MATa trp1Δ63 leu2Δ ura3–52 his3Δ200 lys2–8Δ1 GFPCSE4::HB (pKK1) SPC42-mCh::NAT pGAL1-URA3 Vector (pMB433)*	This study
YMB12682	*MATa trp1Δ63 leu2Δ ura3–52 his3Δ200 lys2–8Δ1 GFPCSE4::HB (pKK1) SPC42-mCh::NAT pGAL1-FLAG-JHD2-URA3 (pMB2163)*	This study
YMB12844	*MATa ura3–52 his3 dam1Δ::DAM1–3HA pGAL1-URA3 Vector (pMB433)*	This study
YMB12845	*MATa ura3–52 his3 dam1Δ::DAM1–3HA pGAL1-FLAG-JHD2-URA3 (pMB2163)*	This study
YMB12846	*MATa ura3–52 his3 dam1Δ::DAM1–3HA ubp8Δ::HIS3 pGAL1-URA3 Vector (pMB433)*	This study
YMB12847	*MATa ura3–52 his3 dam1Δ::DAM1–3HA ubp8Δ::HIS3 pGAL1-FLAG-JHD2-URA3 (pMB2163)*	This study
YMB12850	*MATα leu2 ura3–52 his3 dam1Δ::DAM1–3HA pGAL1-URA3 Vector (pMB433)*	This study
YMB12851	*MATα leu2 ura3–52 his3 dam1Δ::DAM1–3HA pGAL1–6HIS-HA-JHD2-URA3 (pMB2030)*	This study
YMB12852	*MATα leu2 ura3–52 his3 dam1Δ::DAM1–3HA pGAL1–6HIS-HA-jhd2-H427A-URA3 (pMB2153)*	This study
YMB12856	*MATa ura3Δ0 leu2Δ0 his3Δ1 met15Δ0 ubp8Δ::HIS3 pGAL1-URA3 Vector (pMB433)*	This study
YMB12857	*MATa ura3Δ0 leu2Δ0 his3Δ1 met15Δ0 ubp8Δ::HIS3 pGAL1–6HIS-HA-JHD2-URA3 (pMB2030)*	This study
YMB12858	*MATa ura3Δ0 leu2Δ0 his3Δ1 met15Δ0 ctf19Δ::KAN ubp8Δ::HIS3 pGAL1-URA3 Vector (pMB433)*	This study
YMB12859	*MATa ura3Δ0 leu2Δ0 his3Δ1 met15Δ0 ctf19Δ::KAN ubp8Δ::HIS3 pGAL1–6HIS-HA-JHD2-URA3 (pMB2030)*	This study
WT-12380	*MATa ura3Δ0 leu2Δ0 his3Δ1 met15Δ0*	This study
*ctf19Δ*-12381	*MATa ura3Δ0 leu2Δ0 his3Δ1 met15Δ0 ctf19Δ::KAN*	This study
*H3K4A*-12382	*MATa ura3Δ0 leu2Δ0 his3Δ1 met15Δ0 hht1-hhf1∆::NAT hht2-hhf2::[HHTS-HHFS] H3K4A-URA3*	This study
*H3K4Actf19Δ*-12383	*MATa ura3Δ0 leu2Δ0 his3Δ1 met15Δ0 hht1-hhf1∆::NAT hht2-hhf2::[HHTS-HHFS] H3K4A-URA3 ctf19Δ::KAN*	This study
(B) List of plasmids:		
Plasmid	Description	Reference
*pMB433*	*pGAL1-URA3 vector*	Basrai Lab
*pGAL1 JHD2 (pMB2030)*	*pGAL1–6HIS-HA-JHD2-URA3*	Open Biosystems
*pMB2153*	*pGAL1–6HIS-HA-jhd2-H427A-URA3*	This study
*pMB2163*	*pGAL1-FLAG-JHD2-URA3*	This study

### Detection of methylated lysine in Dam1

The yeast strains were grown at 25ºC in YPD or in selective media to LOG phase with constant shaking (200 rpm) and were synchronized in G1 with α-factor (3 μM, RP01002, GenScript Inc.), S-phase with hydroxyurea (0.2 M HU, H8627, Sigma Aldrich) or G2/M with nocodazole (20 μg/mL NOC, M1404, Sigma Aldrich) for 2 hours. For G1 synchronization-release experiments, yeast strains were grown to LOG phase at 25ºC in YPD, synchronized in G1 with α-factor and released into pheromone-free YPD medium. α-factor was re-added to the culture at 100 min after release to block cells in next G1. Samples for flow cytometry and IP were collected at time points after release from G1. For S-phase synchronization-release experiments, strains were grown in YPD to LOG phase at 25ºC, synchronized in S-phase with HU and released into YPD medium. HU was re-added at 100 min after release to block cells in next S-phase. Samples were collected for flow cytometry and IP at time points after release from the S-phase. Flow cytometry was performed to confirm the cell cycle synchronization using Cell Quest software in a BD FACSymphony flow cytometer (BD Biosciences, Boston, MA). Based on the flow cytometry, nuclear position and bud morphology, cells were categorized as G1, S, metaphase, anaphase, and telophase as described previously [29,63,64] using the Zeiss Axioskop 2 microscope (Carl Zeiss Inc., USA). Moreover, we classified cells based on the position of the nucleus with respect to size of the bud. If the bud size is at least half the size of the mother cell and there is a single nuclear mass, we classify them at metaphase. If, however, the bud size is smaller and there is a single nucleus, we categorize these as S-phase [29,63,64].

For G1 arrest-release experiments with overexpression of *JHD2*, strains were grown to LOG phase in 1xSC-Ura with 2% glucose at 25ºC. Cells were collected and washed 3-times with 50 mL of sterile water. Cells were used to prepare OD_600_ = 0.35 (final concentration) in 1xSC-Ura with 2% each galactose+raffinose and grown at 25ºC for 2 hours with α-factor to synchronize cells in G1. Cells were collected, washed 3-times with 50 mL of sterile water and released into pheromone free 1xSC-Ura with 2% each galactose+raffinose media at 25ºC. α-factor was re-added to the culture at 180 min after release to block cells in next G1. Samples for flow cytometry and IP were collected at different time points after release from the G1.

Proteins containing methylated lysine were enriched by immunoprecipitation experiments in three independent biological replicates following identical experimental conditions using methyl-lysine specific antibodies (PA5–77770, Thermo Fisher Scientific). Briefly, yeast cell pellets were dissolved in lysis buffer (20 mM Na_2_HPO_4_, 20 mM NaH_2_PO_4_, 5 mM tetra-sodium pyrophosphate, 50 mM NaF, 1 mM DTT, 10 mM beta-glycerolphosphate, 2 mM EDTA, 1% NP-40, 5 mM NEthylmaleimide, 1 mM PMSF) with protease inhibitor cocktail (P8215; Sigma Aldrich), and were lysed by bead beating (10 times, 40 sec each time) in a FastPrep-5G system (SKU: 116005500, MP Biomedical). Whole cell protein extracts were clarified by centrifugation (13000 rpm for 5 min), normalized for protein concentration using BioRad DC protein assay to obtain an equal value of 100 based on OD_750_ measurements for each sample. Input samples (1/10 volume of IP) were collected. The normalized whole cell extracts (OD_750_ = 100) were used for affinity purification with 15 μL of magnetic beads (30410, Qiagen) conjugated with methyl-lysine antibodies (~15 μg per sample, PA5–77770, Thermo Fisher Scientific) on a roller with constant rotation at 4°C for 16 hrs. Incubated magnetic bead samples were collected by centrifugation (5000 rpm for 1 min), washed once with 1 mL lysis buffer at room temperature for 10 min on a roller with constant rotation followed by one wash with 1 mL 1x TBST (25 mM Tris pH 8.0, 10 mM NEM, 0.3 M NaCl, 0.1% NP-40) as described above. Proteins bound to ^Me^K-magnetic beads were eluted in 50 μL of 1 × Laemmli buffer and used in western blotting. To detect the levels of ^MeK^Dam1 in the IP and total Dam1 in the input samples, eluted proteins were size-fractionated along with input samples by SDS-PAGE on 4–12% Bis-TRIS SDS-polyacrylamide gels and transferred to nitrocellulose membrane using XCell II Blot Module with 35 volts for 60 min following the manufacturer’s recommendations (Invitrogen Inc.). Blots were blocked in 5% milk at room temperature for 30 min and incubated in 1/1000 dilutions of α-HA antibodies (12CA5, Roche) in 5% milk at 4°C for 16 hours on a rocker platform. Blots were washed three-times at room temperature in 1xTBST for 10 min each time and incubated in 1/7500 dilutions of secondary antibodies (HRP-conjugated α-mouse IgG, Cat# NA931V; Cytiva Life Sciences) for 2 hours. After incubation with secondary antibodies, blots were washed again three-times with 1xTBST for 10 min each time, incubated for 2 min in Super Signal West Pico PLUS reagents (Thermo Scientific) and scanned with BioRad ChemiDoc MP Imager using Optimum Auto Exposure settings that selects for full dynamic range and sensitivity required for quantitative analysis (BioRad Inc). Enrichment of ^MeK^Dam1 was determined using a semi-quantitative approach. Protein intensity signals from input and ^MeK^IP samples were quantified from western blots to determine the enrichment of ^MeK^Dam1 using Image J version 1.53t software [117]. Protein bands corresponding to Dam1 were selected using rectangle tool (equal size across the samples), lanes were plotted using analyze option, and band area intensities were determined using the Wand (tracing) tool as described in Image J [117]. Relative enrichment of lysine methylation of Dam1 was measured as a ratio of band area intensities from ^MeK^Dam1 to input Dam1 for each sample, and statistical significance was determined by Student’s *t*-test.

### Immunoprecipitation experiments

Whole cell extracts were prepared by bead beating in a FastPrep-5G bead beating system (SKU: 116005500, MP Biomedical) from yeast cells grown at 25ºC to LOG phase in YPD or in selective medium based on the plasmids used and the cell cycle synchronization (G1 and metaphase). IP experiments were performed with anti-HA (A2095, Sigma Aldrich) and anti-Flag (A2220, Sigma Aldrich) agarose antibodies following the approach as described previously [118] and proteins were eluted using HA peptides (I2149, Sigma Aldrich) or Flag peptides (F3290, Sigma Aldrich). Immunoprecipitated protein samples were analyzed by western blotting as described above. Primary antibodies used were α-Myc (F3165, Sigma Aldrich), α-HA (H6908, Sigma Aldrich), α-FLAG (F7425, Sigma Aldrich), and α-Tub2 [119]. Secondary antibodies were HRP-conjugated α-mouse IgG (NA931V) and HRP-conjugated α-rabbit IgG (NA934V) (Cytiva Life Sciences).

### Protein extractions, and expression analysis

Total protein extracts were prepared using the Trichloroacetic Acid (TCA) protein extraction method [97], and the protein levels were quantified with Bio-Rad DC protein quantitation assay (Bio-Rad Laboratories, Hercules, CA). Protein samples were size-separated on 4–12% Bis-TRIS SDS-polyacrylamide gels by SDS-PAGE, transferred to nitrocellulose membrane, and western blot analysis was performed as described above. Primary antibodies used for western blotting were α-HA (H6908, Sigma Aldrich), α-FLAG (F7425, Sigma Aldrich), α-Myc (M4439, Sigma Aldrich), α-Mif2 (a gift from Pam Meluh), α-GFP (11814460001, Roche), α-Histone H3 (Ab1791, Abcam), α-Histone H3-K4^Me3^ (39160, Active Motif), α-Ctf19 (a gift from Arshad Desai) α-Dam1 [55] and α-Tub2 [119]. Secondary antibodies used for western blotting were HRP-conjugated α-rabbit IgG (NA934V) and HRP-conjugated α-mouse IgG (NA931V) (Cytiva Life Sciences).

### ChIP and qPCR experiments

ChIP was performed in three biological replications using the procedure as described [24,120]. Strains were grown in selective medium at 25˚C to LOG phase and the expression of *JHD2* was induced from the *GAL1* promoter. Protein-DNA complexes were captured using α-GFP (11814460001, Roche), α-Ctf19 (a gift from Arshad Desai), and α-Mif2 (a gift from Pam Meluh) antibodies following the procedure as described previously [24,120]. ChIP-qPCR was performed in a 7500 Fast Real Time PCR System using Fast SYBR Green Master Mix (Applied Biosystems, Foster City, CA) with primers for *CEN1*, *CEN3* and *ACT1* (negative control) regions as described [29]. The PCR amplification conditions were 95ºC for 20 sec, followed by 40 cycles of 95ºC for 3 sec, 60ºC for 30 sec. The enrichment was calculated from three biological replicates using the _ΔΔ_C_T_ method and is shown as percent input [121].

### Cell biology and microscopic assays

A strain in which Cse4 and Spc42 have been tagged with GFP and mCherry, respectively (GFP-Cse4, Spc42-mCherry) carrying vector or *GALJHD2* was grown in selective medium and expression of *JHD2* was induced by the *GAL1* promoter. Cells were imaged at 25°C on an Eclipse Ti wide-field inverted microscope (Nikon) with a 100 × Apo TIRF 1.49 NA objective (Nikon) and Clara charge-coupled device camera (Andor) using Nikon NIS Elements imaging software. All population imaging was performed by switching the channel prior to taking a Z-step. The filter set EGFP/DsRed Dichroic Mirror (86007bs) (480/20 × , 565/25× and 525/40 m, 620/60 m) and ECFP/EYFP/mCherry (89006) (430/24 × , 500/20 × , 572/35 × ; 470/24 m, 535/30 m, 632/60 m) from Chroma Technology, Bellows Falls, Vermont, USA were used following procedure as described previously [122].

### Viability and chromosome segregation assays

For viability assays, wild-type and kinetochore mutant strains containing vector (pMB433 *GAL1*) or *GAL1–6HIS-HA-JHD2* (Open Biosystems) were grown selectively in synthetic media at 25°C. Cells concentrations were determined and OD_600_ = 1.0 cells were collected, dissolved in 1 mL of sterile water and 1:5 serial dilution were made by pipetting 40 μL of cell suspension from each row above to the row immediately below carrying 160 μL of sterile water. Growth assays were carried out by spotting 5 μL from each serial dilution representing equal numbers of cells from three independent transformants for each strain on synthetic medium containing 2% glucose or 2% each galactose+raffinose and grown for 3 days at 25°C.

The frequency of chromosome segregation errors was measured by a colony color assay developed utilizing the mutation in the *ADE2* gene as described previously [96]. In this assay, we used a strain that carries a centromere 3L containing mini-chromosome fragment (CF), which has been used extensively to measure the frequency of chromosome segregation in budding yeast [96,123,124]. The loss of CF leads to the formation of red-sectored colonies instead of a white color yeast colony when cells are plated on synthetic complete media with limiting adenine (1/5^th^). Strains were grown to the LOG phase in medium selecting for the CF and plated on synthetic complete with limiting adenine medium at 25ºC. About 1000 colonies were counted for each strain from three independent transformants. The frequency of CF loss was determined by counting the colonies that showed red-sectors and were normalized to the total number of colonies. Statistical significance was determined by Student’s *t*-test.

### Mass spectrometry assay

The wild-type strain with vector (pMB433 *GAL1*) or *GAL1–6HIS-HA-JHD2* (Open Biosystems) were grown at 25ºC in 1x SC-Ura glucose (2%) medium to LOG phase. Cells were collected, washed with water and inoculated into 1x SC-Ura galactose+raffinose (2% each) medium and grown at 25ºC for 6 hours (~2 generations) to induce the expression of *6HIS-HA-JHD2.* Cells were collected and dissolved in IP lysis buffer [118] with protease inhibitors and subjected to bead-beating in a FastPrep-5G bead beating system (SKU: 116005500, MP Biomedical). Whole cell extracts were collected by centrifugation, and affinity purifications were performed with Ni-NTA agarose (30230, Qiagen Inc.) and anti-HA agarose (A2095, Sigma Aldrich) beads at 4°C for 24 hours. Beads were collected and washed sequentially for 5 min at room temperature with the IP lysis buffer (1x) [118], followed by PBS (1x), and HEPES buffer pH 8.0 (1x). The beads were resuspended in 50 mM HEPES, pH 8.0 and heated at 95°C for 5 min to denature the proteins. The samples were digested overnight with 2 ug of trypsin at 37°C. The digested peptide samples were acidified by Formic acid (FA) to a final concentration of 1% and desalted using Pierce peptide desalting columns according to manufacturer’s protocol (Thermo Fisher Scientific). Peptides were eluted from the columns using 50% AcN/ 0.1% FA, vacuum centrifuged to dryness and stored at -80°C until analyzed by mass spectrometry. The dried peptide fractions were reconstituted in 0.1% TFA and subjected to nanoflow liquid chromatography (Thermo Ultimat:M3000RSLC nano LC system, Thermo Fisher Scientific) coupled to an Orbitrap Eclipse mass spectrometer (Themo Scientific, CA). Peptides were separated using a low pH gradient using a 5–50% ACN over 120 minutes in mobile phase containing 0.1% formic acid at 300 nl/min flow rate. Full MS1 scans were performed in the Orbitrap at a resolution of 120,000 with an ion accumulation target set at 4e5 and max IT set at 50 ms over a mass range of 350–1600 m/z. lons with determined charge states between 2 and 5 were selected for MS2 scans in the orbitrap with HCD fragmentation (NCE 30%; maximum injection time 22 ms; AGC 5 × 104) at 15K resolution. Acquired MS/MS spectra ware searched against Yeast proteome fasta (version: Uniprot_yeast_aug2020_reviewed_Canonical fasta) using a SEQUEST and Percolator validator algorithms in the Proteome Discoverer 2.4 software (Thermo Fisher Scientific). The precursor ion tolerance was set at 10 ppm and the fragment ions tolerance was set at 0.02 Da along with methionine oxidation and phosphorylation of serine, threonine, and tyrosine included as dynamic modification. Dynamic modification of lysine residues for acetylation, mono-methylation, di-methylation and tri-methylation was also included. Trypsin was specified as the proteolytic enzyme, with up to 2 missed cleavage sites allowed. Searches used a reverse sequence decoy strategy to control for the false peptide discovery and identification were validated by Percolator software.

## Supporting information

S1 Fig Methyl lysine antibodies (^Me^K) specifically detect methylated proteins. Wild-type strains carrying HA-Cse4 (YMB9595) or HA-Cse4^16KR^ (YMB9596) expressed from the *GAL1* promoter were grown in 1x Sc-Ura galactose+raffinose (2% each) medium to logarithmic phase at 25°C. Whole cell extracts were prepared, and methylated proteins were enriched by immunoprecipitation with anti-methyl lysine antibodies (PA5–77770, Thermo Fisher Scientific). Western blotting using α-HA antibodies was performed. IN, input; ^MeK^IP, immunoprecipitated samples.
(TIF)

S2 Fig ^MeK^Dam1 is cell cycle regulated. Additional biological replicates of ⍺-factor arrest and release (**Related to Fig 1**). **(A)** Flow cytometry analysis showing DNA content and cell cycle progression. **(B)** The levels of ^MeK^Dam1 are higher in metaphase cells. Western blotting using α-HA (Dam1–3HA) antibodies. IN, input; ^MeK^IP, samples immunoprecipitated with methyl lysine antibodies.
(TIF)

S3 Fig ^MeK^Dam1 levels are increased in metaphase. Additional biological replicates of HU arrest and release (**Related to Fig 2**). **(A and B)** Flow cytometry showing DNA content and cell cycle progression. **(C and D)** ^MeK^Dam1 enrichment is higher in metaphase cells. Western blotting using α-HA (Dam1–3HA) antibodies. IN, input; ^MeK^IP, samples immunoprecipitated with methyl lysine antibodies.
(TIF)

S4 Fig Dam1 interacts *in vivo* with Set1 in a cell cycle dependent manner. Additional biological replicates (**Related to Fig 3**). **(A and C)** Flow cytometry analysis showing DNA content. **(B and D)** *In vivo* interaction of Dam1 with Set1 in G1, S-phase and metaphase cells. Proteins immunoprecipitated using α-HA conjugated agarose beads were analysed by western blotting with α-HA (Dam1), and α-Myc (Set1) antibodies. IN, input; IP, immunoprecipitated samples.
(TIF)

S5 Fig Enrichment of ^MeK^Dam1 requires kinetochore-microtubule interactions. Additional biological replicates (**Related to Fig 4**). **(A and C)** Flow cytometry showing DNA content and cell cycle stages. **(B and D)** The levels of ^MeK^Dam1 are reduced in nocodazole treated G2/M cells. Methylated proteins were enriched by immunoprecipitation with anti-methyl lysine antibodies and analysed by western blotting using α-HA (Dam1–3HA) antibodies. IN, input; ^MeK^IP, immunoprecipitated samples.
(TIF)

S6 Fig Jhd2 interacts with Dam1 *in vivo.* Additional biological replicates (**Related to Fig 5**). **(A and B)** Flow cytometry analysis representing DNA content. **(C, D, E and F)** *In vivo* interaction of Jhd2 with Dam1. Proteins were immunoprecipitated with α-HA conjugated and α-Flag conjugated agarose beads and analysed by western blotting with α-HA (Dam1), and α-Flag (Jhd2) antibodies. IN, input; IP, immunoprecipitated samples. Tub2 was used as a control.
(TIF)

S7 Fig Jhd2 contributes to demethylation of Dam1. Additional biological replicates (**Related to Fig 6**). **(A and B)** Flow cytometry analysis showing DNA content. **(C and D)** Western blots showing protein levels of Jhd2-Flag expressed from the *GAL1* promoter. **(E and F)** Overexpression of *JHD2* results in reduction in the levels of ^MeK^Dam1. Methylated proteins were immunoprecipitated with methyl lysine antibodies and analysed by western blotting using α-HA (Dam1–3HA) antibodies. IN, input; ^MeK^IP, immunoprecipitated samples. **(G and H)** Overexpression of *JHD2* results in reduction in the levels of Histone H3-K4^Me3^. Methylated proteins were immunoprecipitated with methyl lysine antibodies and analysed by western blotting using with histone H3 and histone H3K4^Me3^ antibodies. IN, input; ^MeK^IP, immunoprecipitated samples.
(TIF)

S8 Fig Jhd2 contributes to demethylation of Dam1 in metaphase cells. Additional biological replicates (**Related to Fig 7**). **(A and B)** Flow cytometry showing DNA content. **(C and D)** Overexpression of *JHD2* results in reduction in the levels of ^MeK^Dam1 in metaphase cells. Methylated proteins were immunoprecipitated with methyl lysine antibodies and analysed by western blotting using α-HA (Dam1–3HA) antibodies. IN, input; ^MeK^IP, immunoprecipitated samples.
(TIF)

S9 Fig Overexpression of Jhd2 throughout the cell cycle contributes to demethylation of Dam1. Wild-type strain expressing HA-tagged Dam1 (Dam1–3HA) from its endogenous promoter carrying *pGAL1*-*URA3* vector (strain# YMB12844) or *pGAL1-FLAG-JHD2-URA3* (strain# YMB12845) was grown at 25°C to LOG phase in 1xSC-Ura with 2% glucose at 25ºC. Cells were collected and washed 3-times with 50 mL of sterile water. Cells were used to prepare OD600 = 0.35 (final concentration) in 1xSC-Ura with 2% each galactose+raffinose and grown at 25ºC for 2 hours with ⍺-factor to synchronize cells in G1. Cells were collected, washed 3-times with 50 mL of sterile water and released into pheromone free 1xSC-Ura with 2% each galactose+raffinose media at 25ºC. ⍺-factor was re-added to the culture at 180 min after release to block cells in next G1. **(A)** Flow cytometry showing DNA content and cell cycle progression. **(B)** Cell cycle stages of samples from (A) determined as described in Fig 2B. Different stages: G1, S-phase **(S)**, metaphase **(M)**, anaphase **(A)**, and telophase **(T)**. Average ± SD from three biological replicates is shown. **(C)** Overexpression of *JHD2* results in reduced levels of ^MeK^Dam1 through the cell cycle. Methylated proteins were enriched by IP with anti-methyl lysine antibodies using whole cell extracts from (A) and analyzed by western blotting using α-HA (Dam1–3HA) antibodies. IN, input; ^MeK^IP, immunoprecipitated samples. **(D)** Relative enrichment of ^MeK^Dam1 in vector and *GALJHD2* strains. Enrichment was determined as described in Fig 1D. Three biological replicates were done. Average ±SE is shown. *p value <0.01, Student’s *t*-test.
(TIF)

S10 Fig *GALJHD2*-induced growth defects in *ctf19Δ* is not suppressed by deletion of *UBP8.* 5 μL of cell suspension (OD_600_ = 1 and its 5-fold dilutions) of *ctf19Δ* with *pGAL1-URA3* vector (strain# YMB12274) or *pGAL1–6HIS-HA-JHD2-URA3* (strain# YMB12248); *ubp8Δ* with *pGAL1-URA3* vector (strain# YMB12856) or *pGAL1–6HIS-HA-JHD2-URA3* (strain# YMB12857); *ctf19Δ ubp8Δ* with *pGAL1-URA3* vector (strain# YMB12858) or *pGAL1–6HIS-HA-JHD2-URA3* (strain# YMB12859); and wild type with *pGAL1-URA3* vector (strain# YMB12282) or *pGAL1–6HIS-HA-JHD2-URA3* (strain# YMB12281) were plated on SC-URA with glucose (2%) or galactose+raffinose (2% each) plates and grown at 25°C for 3 days.
(TIF)

S11 Fig Deletion of *UBP8* does not counteract *GALJHD2*-mediated demethylation of Dam1. Wild type strain carrying *pGAL1*-*URA3* vector (strain# YMB12844) or *pGAL1-FLAG-JHD2-URA3* (strain# YMB12845) and *ubp8△* strain carrying *pGAL1*-*URA3* vector (strain# YMB12846) or *pGAL1-FLAG-JHD2-URA3* (strain# YMB12847) were grown at 25°C to LOG phase in 1xSC-Ura media with galactose+raffinose (2% each) for 3 hours. All strains had HA-tagged Dam1 (Dam1–3HA) expressed from its endogenous promoter. **(A)** Flow cytometry showing DNA content. **(B)** Cell cycle stages of samples from (A) determined as described in Fig 2B. Different stages: G1, S-phase **(S)**, metaphase **(M)**, anaphase **(A)**, and telophase **(T)**. Average ± SD from three biological replicates is shown. **(C)** Overexpression of *JHD2* results in reduced levels of ^MeK^Dam1 in *ubp8△* strain. Methylated proteins were enriched by IP with anti-methyl lysine antibodies using whole cell extracts from (A) and analyzed by western blotting using α-HA (Dam1–3HA) antibodies. IN, input; ^MeK^IP, immunoprecipitated samples. **(D)** Relative enrichment of ^MeK^Dam1. Enrichment was determined as described in Fig 1D. Three biological replicates were done. Average ± SE is shown. **p value <0.01, Student’s *t*-test.
(TIF)

S12 Fig Reduced *CEN* association of kinetochore proteins Cse4, Mif2 and Ctf19 in *GALJHD2* strain. Additional biological replicates (**Related to Fig 9**). **(A and B)** Flow cytometry analysis representing DNA content. **(C)** ChIP-qPCR data showing *CEN* enrichment of Cse4. **(D and E)** Western blots showing protein expression of Cse4. **(F)** Flow cytometry analysis representing DNA content of samples used in ChIP-qPCR of Mif2 and Ctf19. **(G)** Cell cycle stages of samples used in ChIP-qPCR of Mif2 and Ctf19. **(H)** ChIP-qPCR data showing *CEN* enrichment of Mif2. **(I)** ChIP-qPCR data showing *CEN* enrichment of Ctf19. **(J and K)** Western blots showing protein expression of Mif2 and Ctf19.
(TIF)

S13 Fig *CEN* association of Ctf19 is not affected in *H3K4A* strain. Wild type (strain# WT-12380) and histone *H3K4A* (strain# *H3K4A*-12382) strains were grown at 25°C to LOG phase in YPD for ChIP experiments. **(A)** Flow cytometry analysis showing DNA content. **(B)** Cell cycle stages of samples from (A) were determined as described in Fig 2B. Cell cycle stages are: G1, S-phase **(S)**, metaphase **(M)**, anaphase **(A)**, and telophase **(T)**. Average ± SD from three biological replicates is shown. **(C)** Levels of Ctf19 are not affected at *CEN* chromatin in *H3K4A* strains. ChIP was performed with α-Ctf19 antibodies using chromatin from strains in (A). Enrichment of Ctf19 at *CENs* (*CEN1* and *CEN3*) and a negative control (*ACT1*) was determined by qPCR and is presented as % input. Average from three biological replicates ±SE. ns = not significant, Student’s *t*-test. **(D)** Expression of Ctf19 in wild type, and *H3K4A* strains are similar. The western blotting of protein extracts was done using α-Ctf19 and α-Tub2 (loading control) antibodies.
(TIF)

S14 Fig Jhd2-mediated demethylation of Dam1 is mediated by its catalytic activity. Additional biological replicates (**Related to Fig 10E and 10F**). **(A, B and C)** Flow cytometry showing DNA content. **(D)** Cell cycle stages of samples from (A) were determined as described in Fig 2B. G1, S-phase **(S)**, metaphase **(M)**, anaphase **(A)**, and telophase **(T)** are shown as average ± SD from three biological replicates. **(E and F)** Overexpression of *JHD2* results in reduction in the levels of ^MeK^Dam1 but not its catalytic mutant *jhd2-H427A*. Methylated proteins were immunoprecipitated with methyl lysine antibodies and analysed by western blotting using α-HA (Dam1–3HA) antibodies. IN, input; ^MeK^IP, immunoprecipitated samples.
(TIF)

S1 Table *In vivo* interactors of Jhd2 identified by mass spectrometry. (XLSX)

S2 Table Overexpression of *JHD2* leads to CIN. Chromosome loss rate data from different biological replicates (**Related to Fig 10**). **(A)** Overexpression of *JHD2* causes errors in chromosome segregation. **(B)** *GALJHD2*-induced chromosome loss is mediated by its catalytic activity.
(DOCX)

S1 Datas Western blots and raw experimental data from the study. (PDF)
