# Supplementary figures and images for: Cell cycle dependent methylation of Dam1 contributes to kinetochore integrity and faithful chromosome segregation
Source: PLoS Genet. 2025 Jun 16;21(6):e1011760. doi: 10.1371/journal.pgen.1011760 (PMC12204631; doi:10.1371/journal.pgen.1011760)

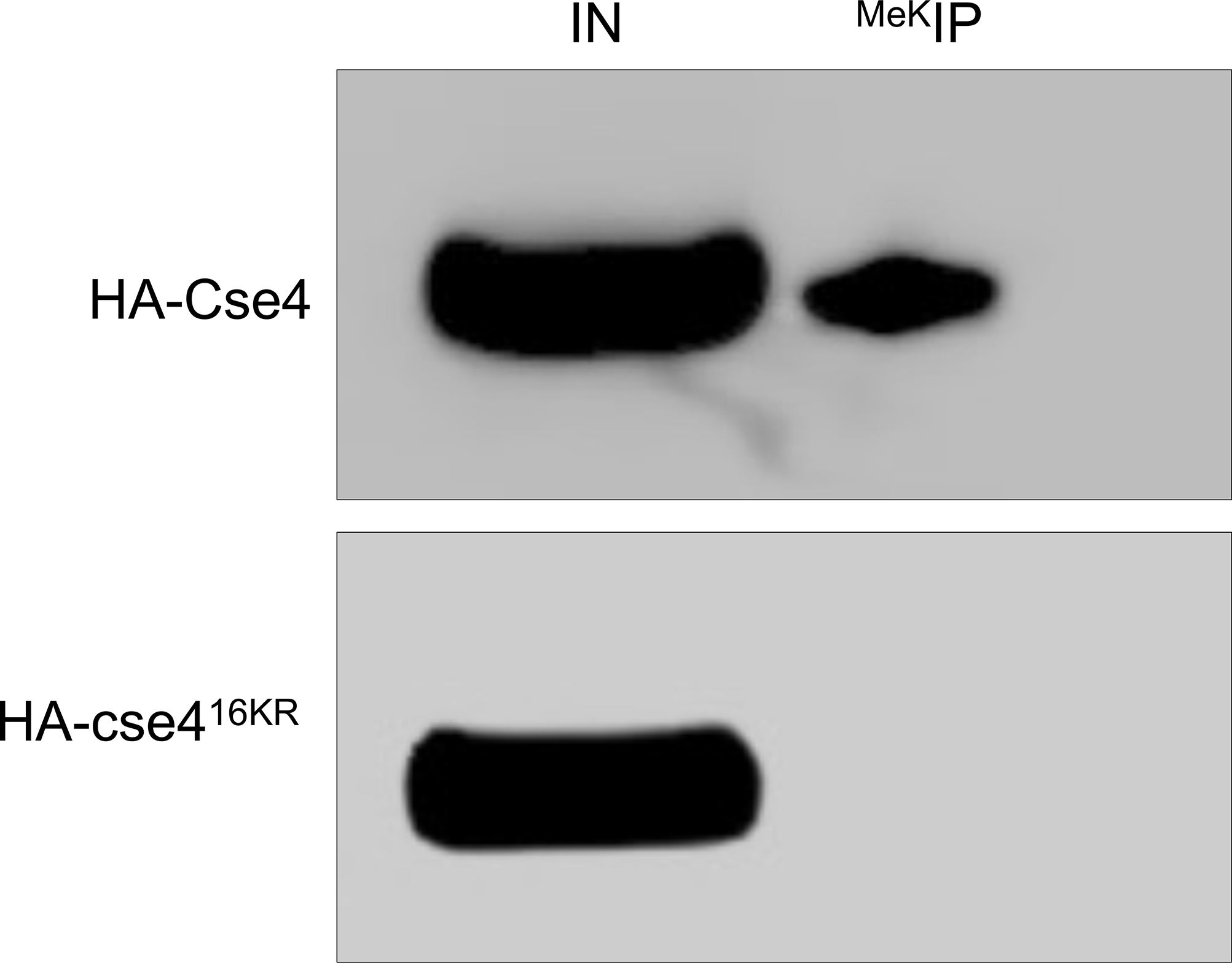

Supplement: S1 Fig — Wild-type strains carrying HA-Cse4 (YMB9595) or HA-Cse416KR (YMB9596) expressed from the GAL1 promoter were grown in 1x Sc-Ura galactose+raffinose (2% each) medium to logarithmic phase at 25°C. Whole cell extracts were prepared, and methylated proteins were enriched by immunoprecipitation with anti-methyl lysine antibodies (PA5–77770, Thermo Fisher Scientific). Western blotting using α-HA antibodies was performed. IN, input; MeKIP, immunoprecipitated samples. (TIF) [file pgen.1011760.s001.tif]

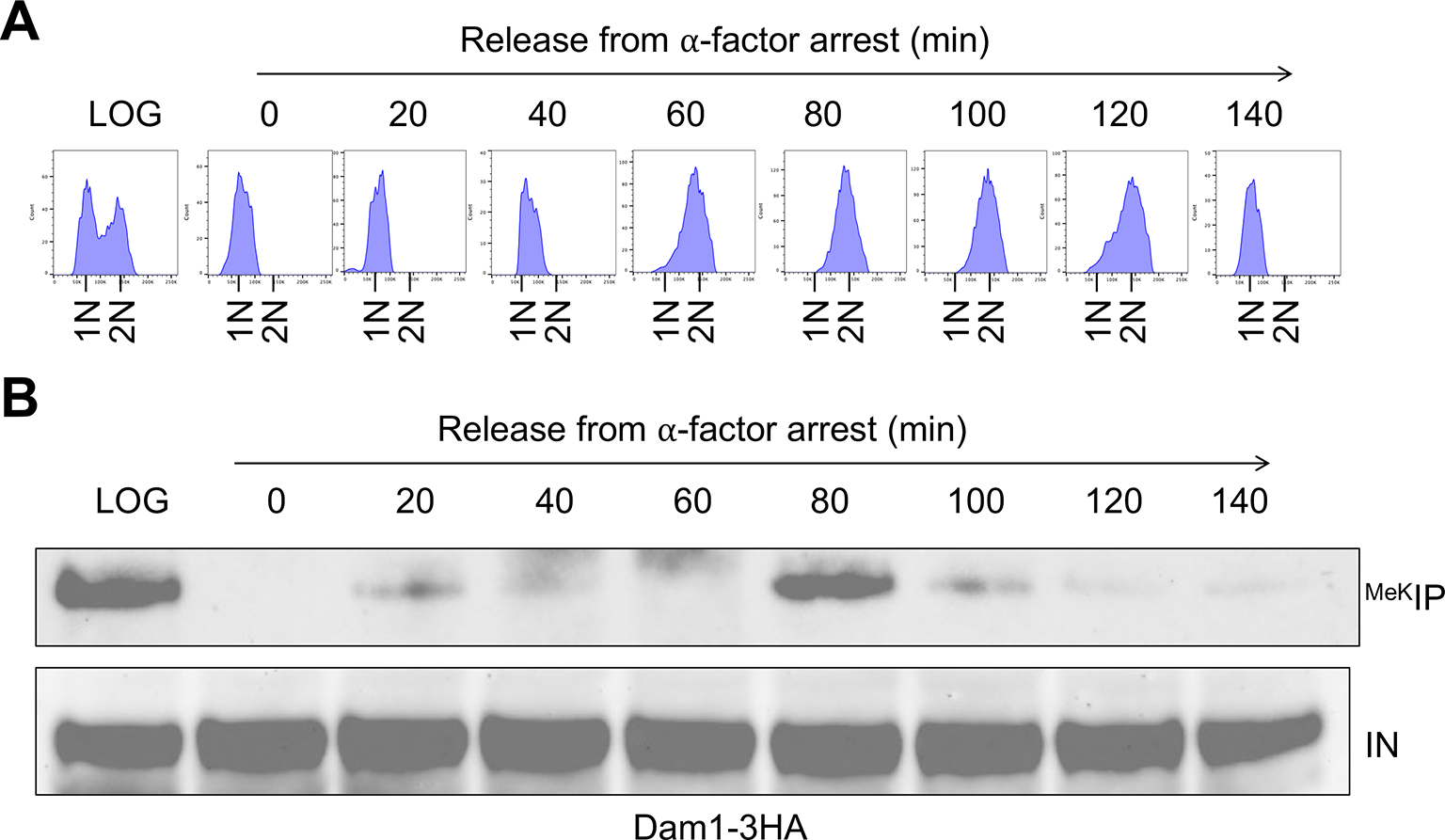

Supplement: S2 Fig — Additional biological replicates of ⍺-factor arrest and release (Related to Fig 1). (A) Flow cytometry analysis showing DNA content and cell cycle progression. (B) The levels of MeKDam1 are higher in metaphase cells. Western blotting using α-HA (Dam1–3HA) antibodies. IN, input; MeKIP, samples immunoprecipitated with methyl lysine antibodies. (TIF) [file pgen.1011760.s002.tif]

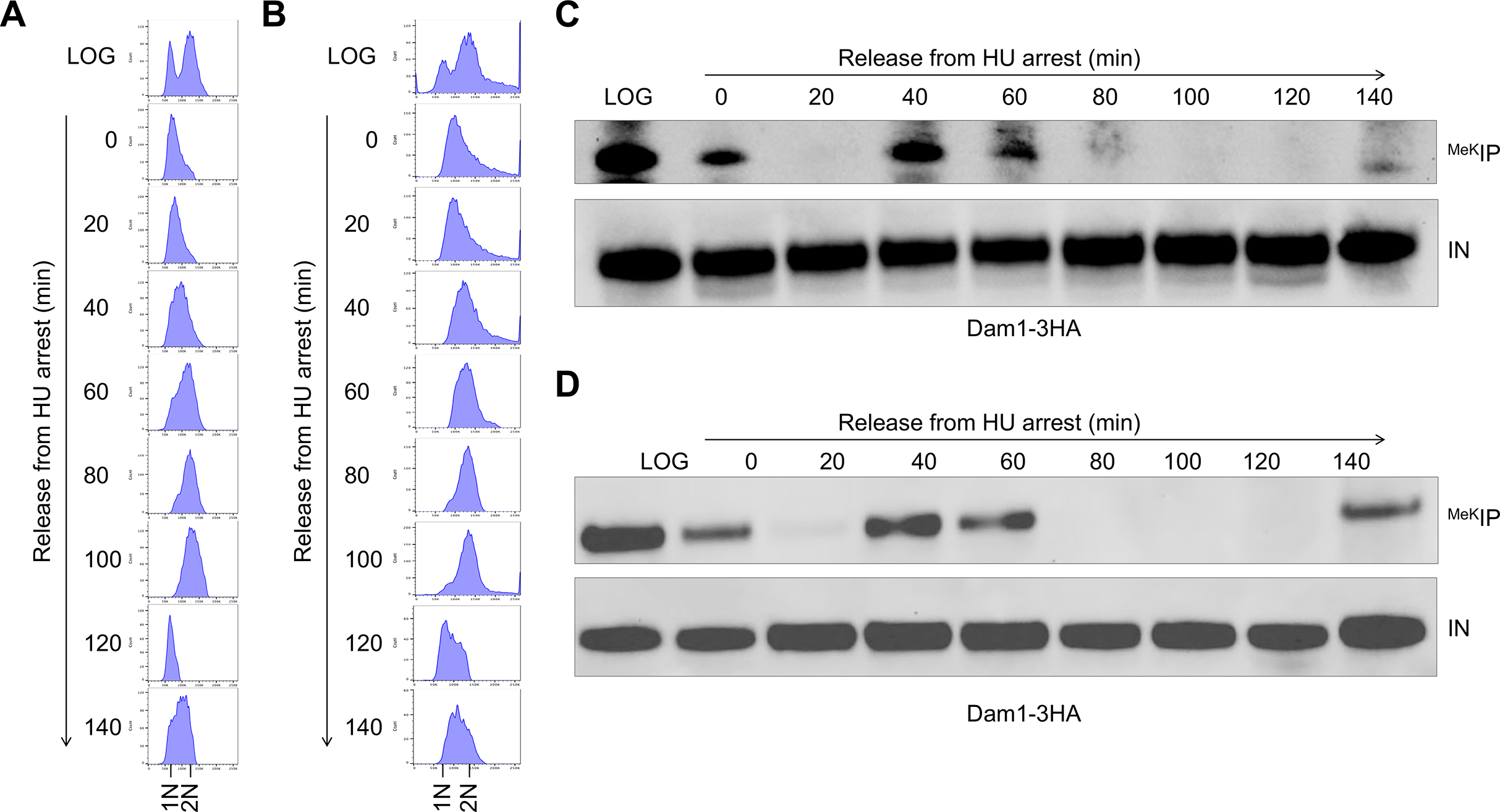

Supplement: S3 Fig — Additional biological replicates of HU arrest and release (Related to Fig 2). (A and B) Flow cytometry showing DNA content and cell cycle progression. (C and D) MeKDam1 enrichment is higher in metaphase cells. Western blotting using α-HA (Dam1–3HA) antibodies. IN, input; MeKIP, samples immunoprecipitated with methyl lysine antibodies. (TIF) [file pgen.1011760.s003.tif]

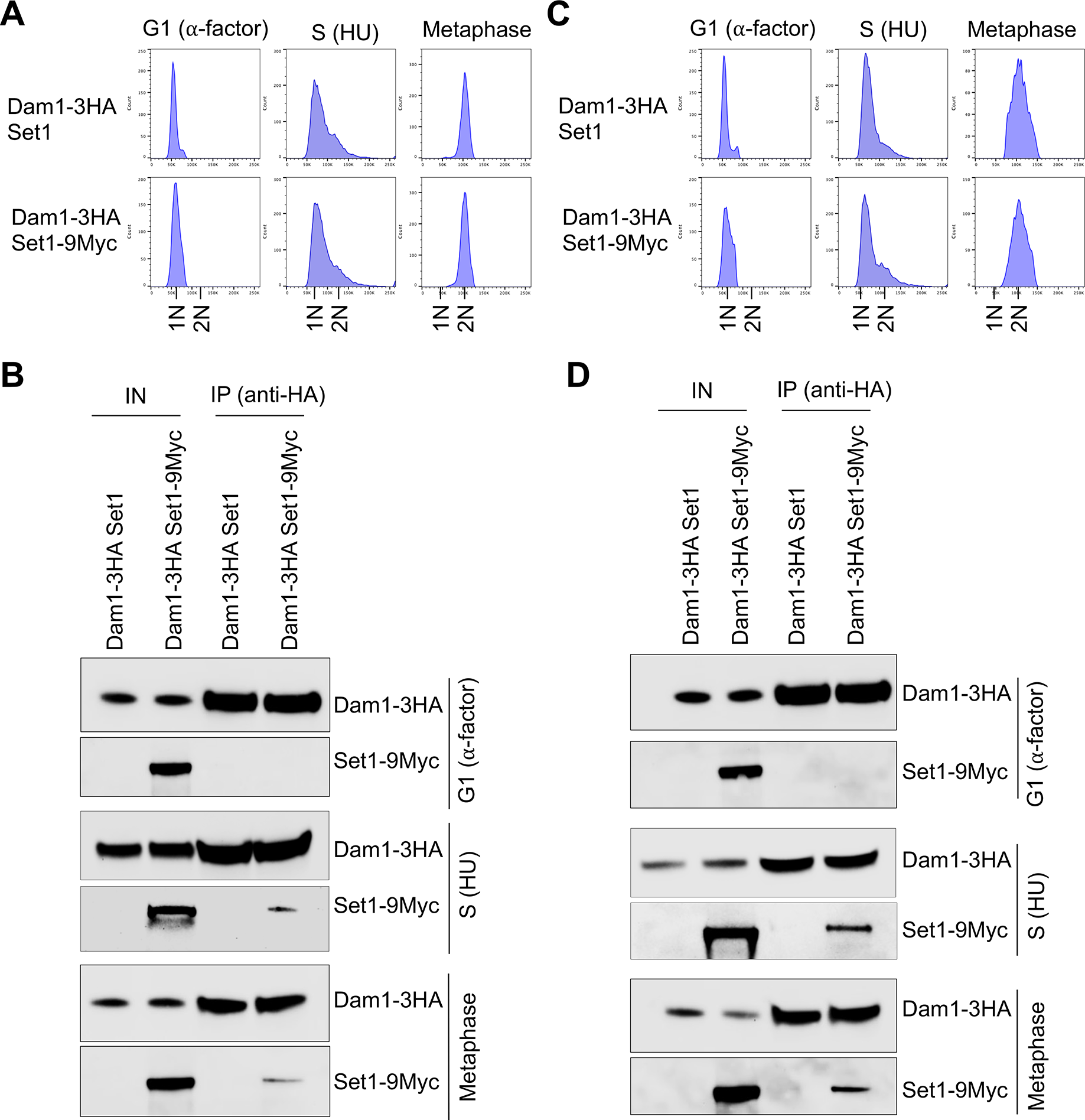

Supplement: S4 Fig — Additional biological replicates (Related to Fig 3). (A and C) Flow cytometry analysis showing DNA content. (B and D) In vivo interaction of Dam1 with Set1 in G1, S-phase and metaphase cells. Proteins immunoprecipitated using α-HA conjugated agarose beads were analysed by western blotting with α-HA (Dam1), and α-Myc (Set1) antibodies. IN, input; IP, immunoprecipitated samples. (TIF) [file pgen.1011760.s004.tif]

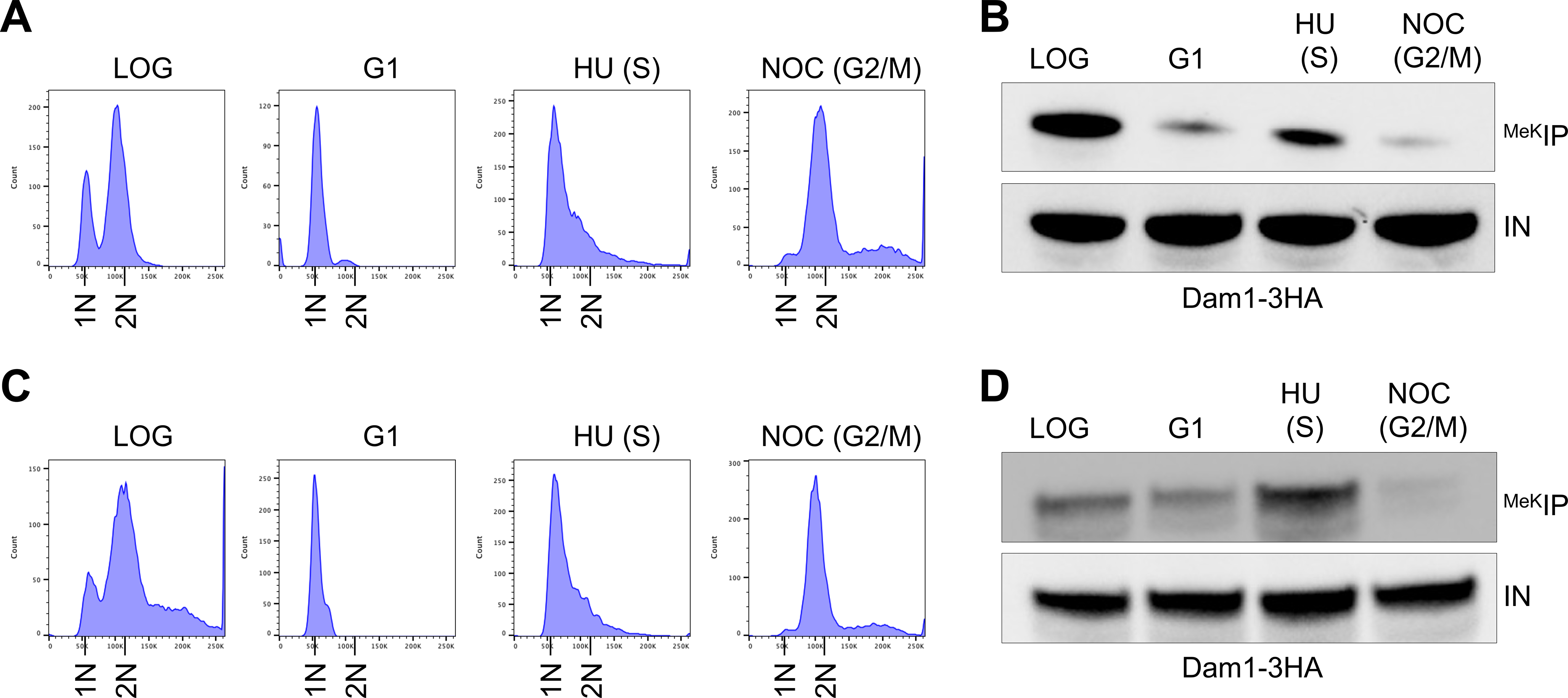

Supplement: S5 Fig — Additional biological replicates (Related to Fig 4). (A and C) Flow cytometry showing DNA content and cell cycle stages. (B and D) The levels of MeKDam1 are reduced in nocodazole treated G2/M cells. Methylated proteins were enriched by immunoprecipitation with anti-methyl lysine antibodies and analysed by western blotting using α-HA (Dam1–3HA) antibodies. IN, input; MeKIP, immunoprecipitated samples. (TIF) [file pgen.1011760.s005.tif]

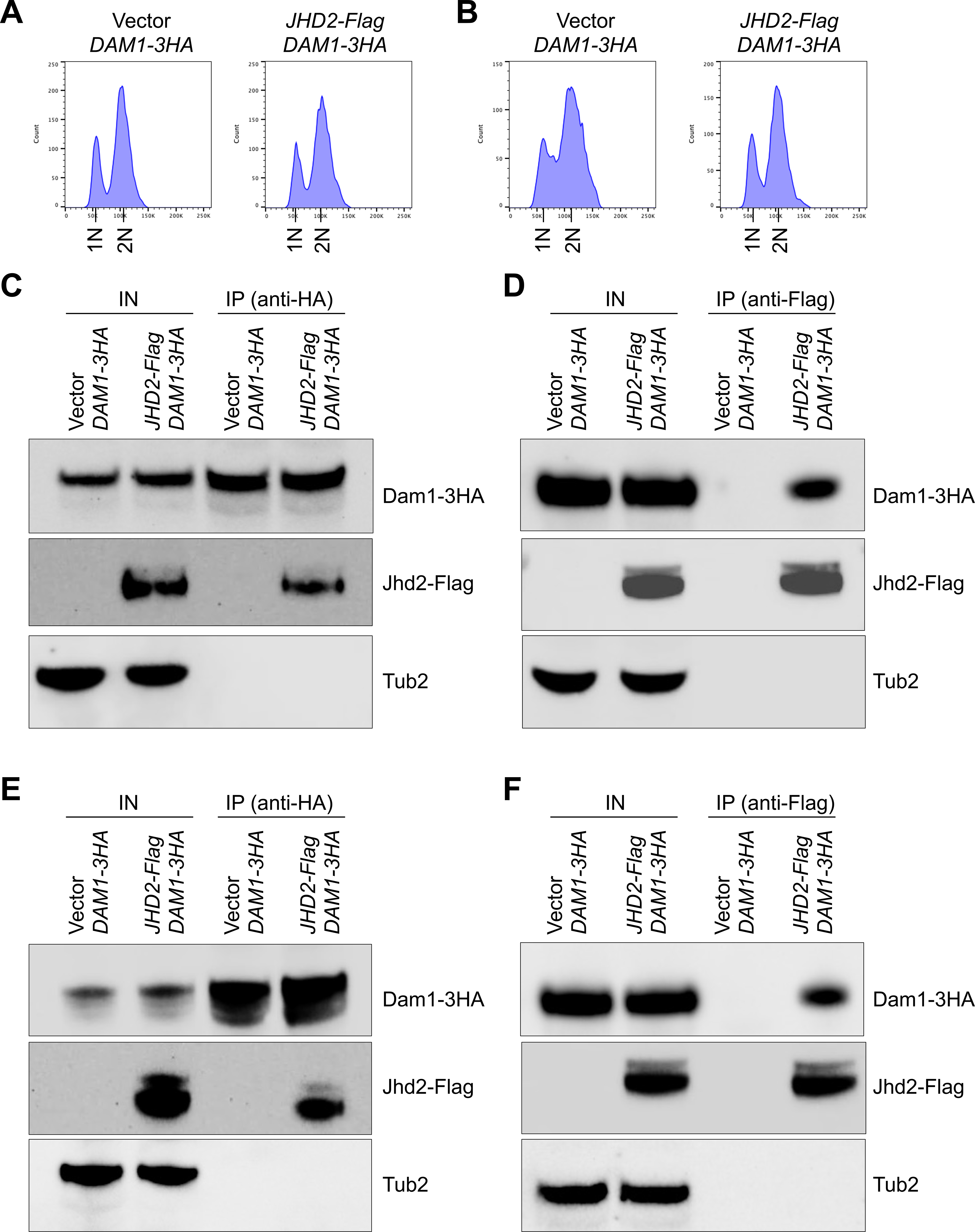

Supplement: S6 Fig — Additional biological replicates (Related to Fig 5). (A and B) Flow cytometry analysis representing DNA content. (C, D, E and F) In vivo interaction of Jhd2 with Dam1. Proteins were immunoprecipitated with α-HA conjugated and α-Flag conjugated agarose beads and analysed by western blotting with α-HA (Dam1), and α-Flag (Jhd2) antibodies. IN, input; IP, immunoprecipitated samples. Tub2 was used as a control. (TIF) [file pgen.1011760.s006.tif]

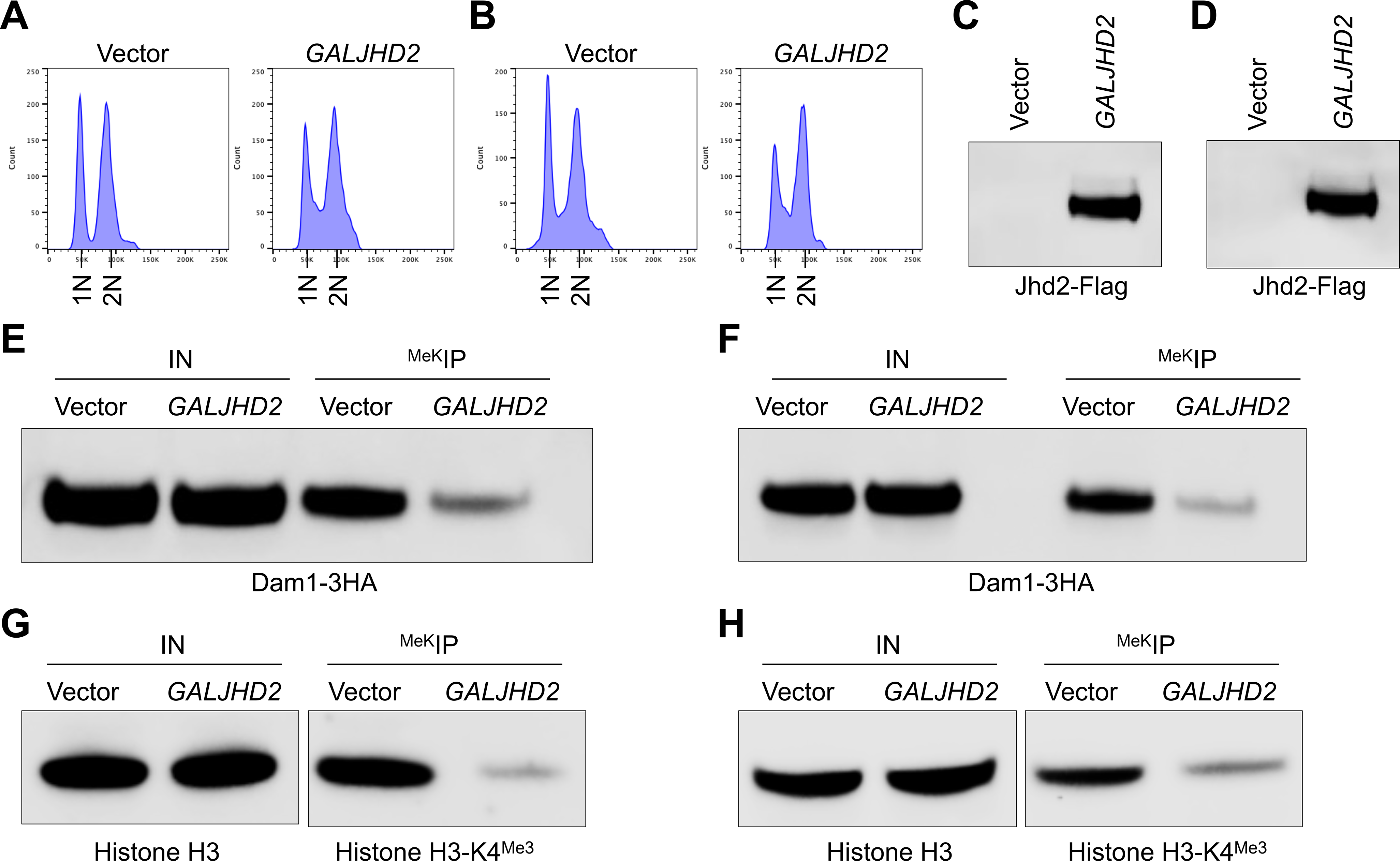

Supplement: S7 Fig — Additional biological replicates (Related to Fig 6). (A and B) Flow cytometry analysis showing DNA content. (C and D) Western blots showing protein levels of Jhd2-Flag expressed from the GAL1 promoter. (E and F) Overexpression of JHD2 results in reduction in the levels of MeKDam1. Methylated proteins were immunoprecipitated with methyl lysine antibodies and analysed by western blotting using α-HA (Dam1–3HA) antibodies. IN, input; MeKIP, immunoprecipitated samples. (G and H) Overexpression of JHD2 results in reduction in the levels of Histone H3-K4Me3. Methylated proteins were immunoprecipitated with methyl lysine antibodies and analysed by western blotting using with histone H3 and histone H3K4Me3 antibodies. IN, input; MeKIP, immunoprecipitated samples. (TIF) [file pgen.1011760.s007.tif]

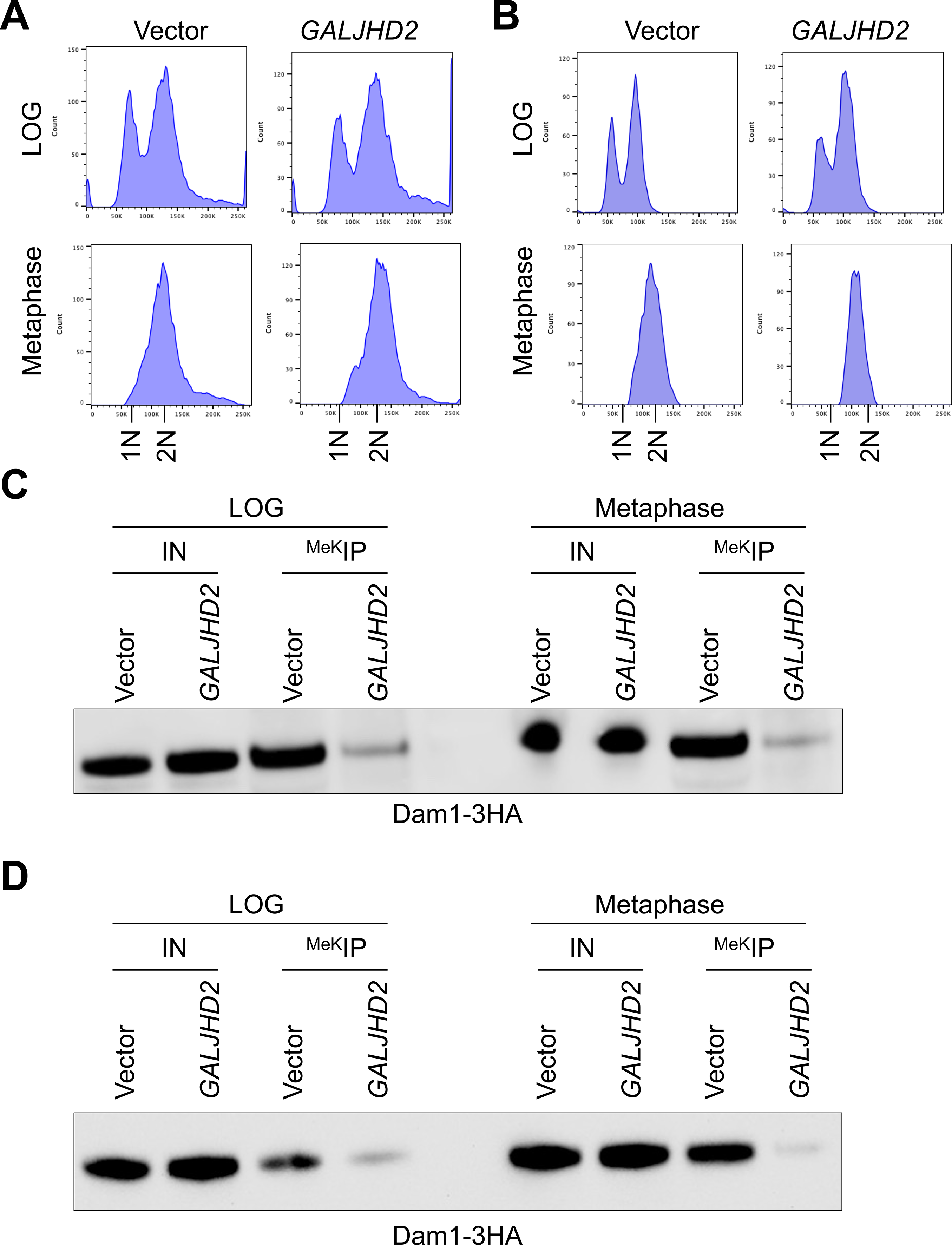

Supplement: S8 Fig — Additional biological replicates (Related to Fig 7). (A and B) Flow cytometry showing DNA content. (C and D) Overexpression of JHD2 results in reduction in the levels of MeKDam1 in metaphase cells. Methylated proteins were immunoprecipitated with methyl lysine antibodies and analysed by western blotting using α-HA (Dam1–3HA) antibodies. IN, input; MeKIP, immunoprecipitated samples. (TIF) [file pgen.1011760.s008.tif]

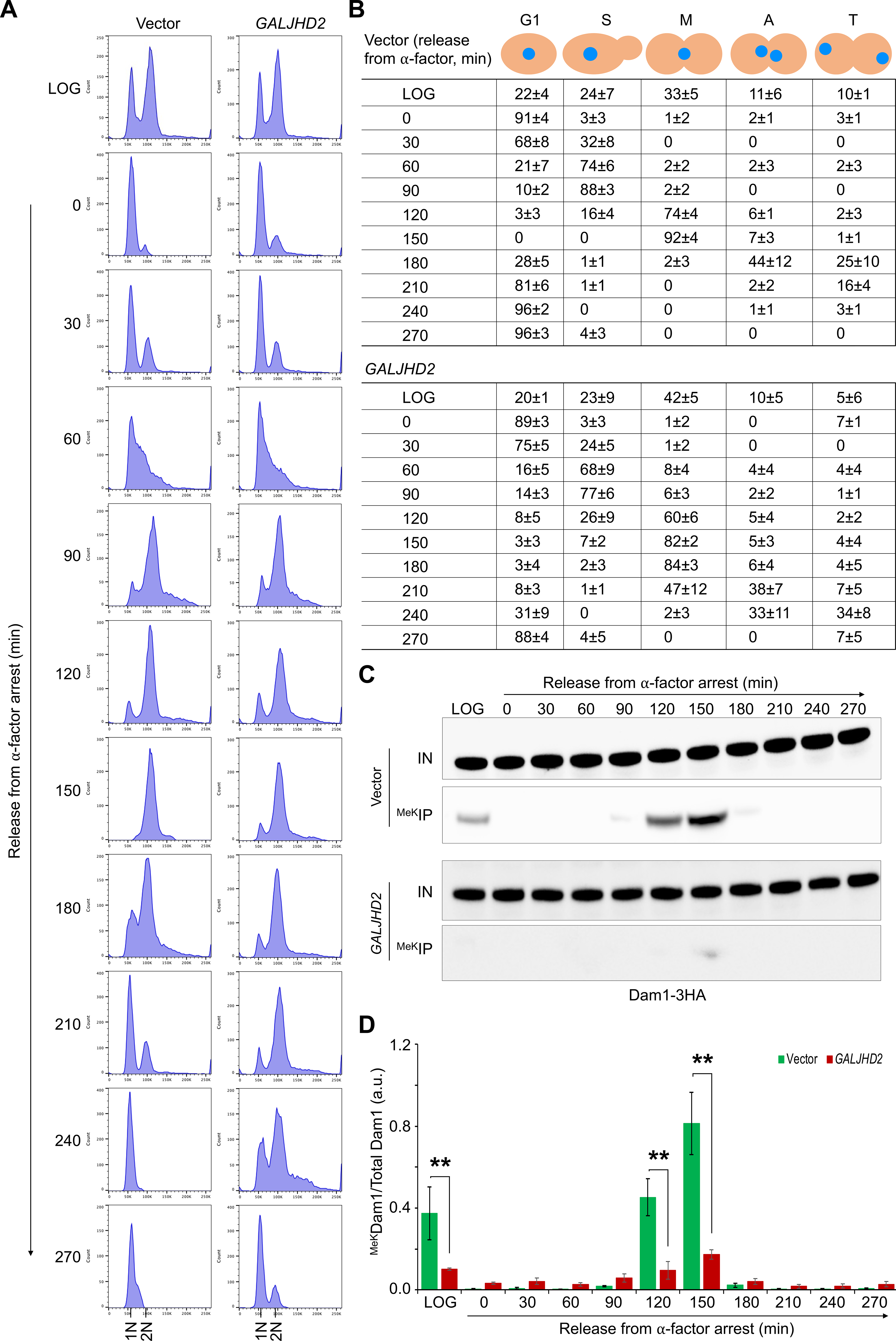

Supplement: S9 Fig — Wild-type strain expressing HA-tagged Dam1 (Dam1–3HA) from its endogenous promoter carrying pGAL1-URA3 vector (strain# YMB12844) or pGAL1-FLAG-JHD2-URA3 (strain# YMB12845) was grown at 25°C to LOG phase in 1xSC-Ura with 2% glucose at 25ºC. Cells were collected and washed 3-times with 50 mL of sterile water. Cells were used to prepare OD600 = 0.35 (final concentration) in 1xSC-Ura with 2% each galactose+raffinose and grown at 25ºC for 2 hours with ⍺-factor to synchronize cells in G1. Cells were collected, washed 3-times with 50 mL of sterile water and released into pheromone free 1xSC-Ura with 2% each galactose+raffinose media at 25ºC. ⍺-factor was re-added to the culture at 180 min after release to block cells in next G1. (A) Flow cytometry showing DNA content and cell cycle progression. (B) Cell cycle stages of samples from (A) determined as described in Fig 2B. Different stages: G1, S-phase (S), metaphase (M), anaphase (A), and telophase (T). Average ± SD from three biological replicates is shown. (C) Overexpression of JHD2 results in reduced levels of MeKDam1 through the cell cycle. Methylated proteins were enriched by IP with anti-methyl lysine antibodies using whole cell extracts from (A) and analyzed by western blotting using α-HA (Dam1–3HA) antibodies. IN, input; MeKIP, immunoprecipitated samples. (D) Relative enrichment of MeKDam1 in vector and GALJHD2 strains. Enrichment was determined as described in Fig 1D. Three biological replicates were done. Average ±SE is shown. *p value <0.01, Student’s t-test. (TIF) [file pgen.1011760.s009.tif]

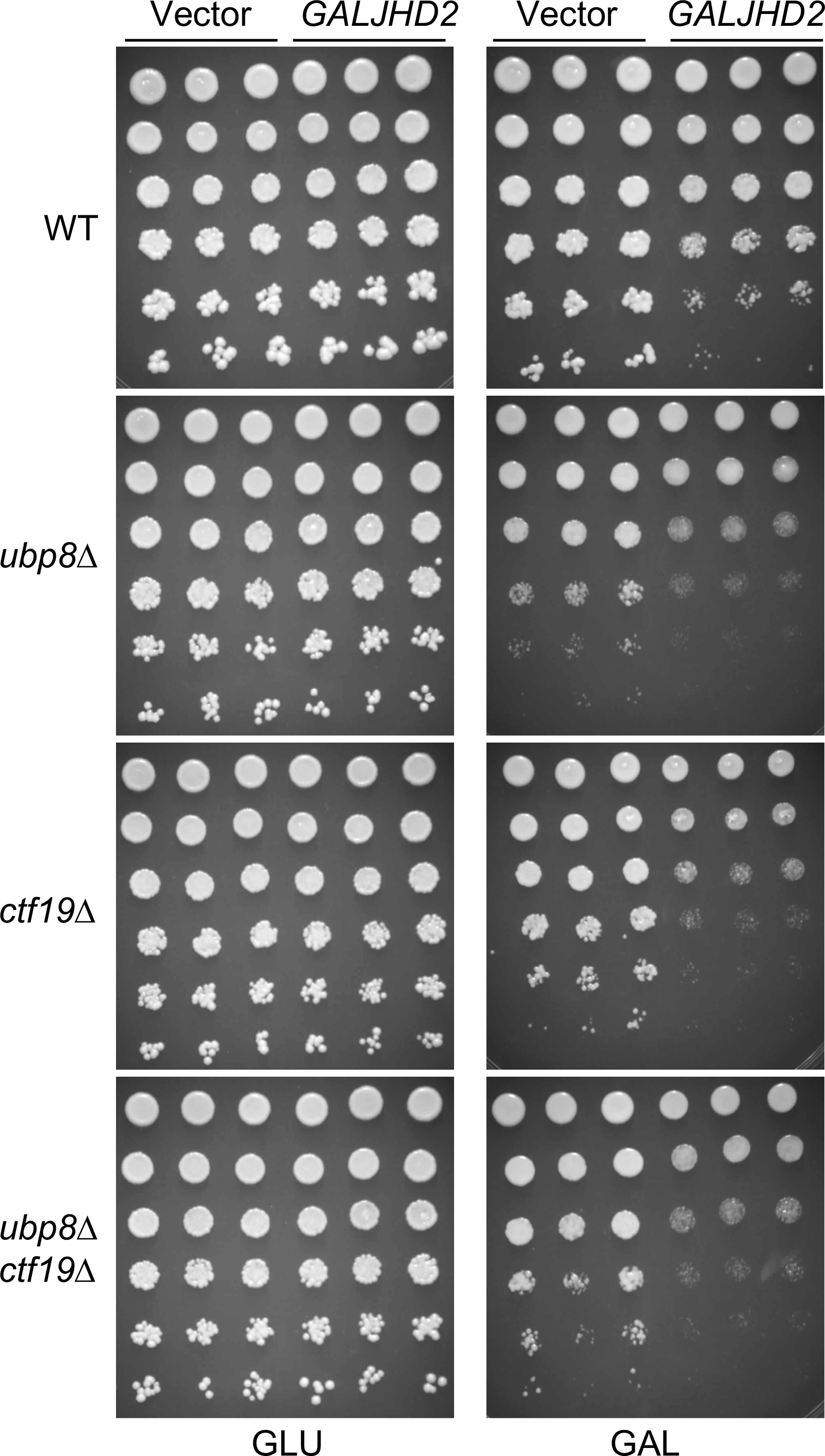

Supplement: S10 Fig — 5 μL of cell suspension (OD600 = 1 and its 5-fold dilutions) of ctf19Δ with pGAL1-URA3 vector (strain# YMB12274) or pGAL1–6HIS-HA-JHD2-URA3 (strain# YMB12248); ubp8Δ with pGAL1-URA3 vector (strain# YMB12856) or pGAL1–6HIS-HA-JHD2-URA3 (strain# YMB12857); ctf19Δ ubp8Δ with pGAL1-URA3 vector (strain# YMB12858) or pGAL1–6HIS-HA-JHD2-URA3 (strain# YMB12859); and wild type with pGAL1-URA3 vector (strain# YMB12282) or pGAL1–6HIS-HA-JHD2-URA3 (strain# YMB12281) were plated on SC-URA with glucose (2%) or galactose+raffinose (2% each) plates and grown at 25°C for 3 days. (TIF) [file pgen.1011760.s010.tif]

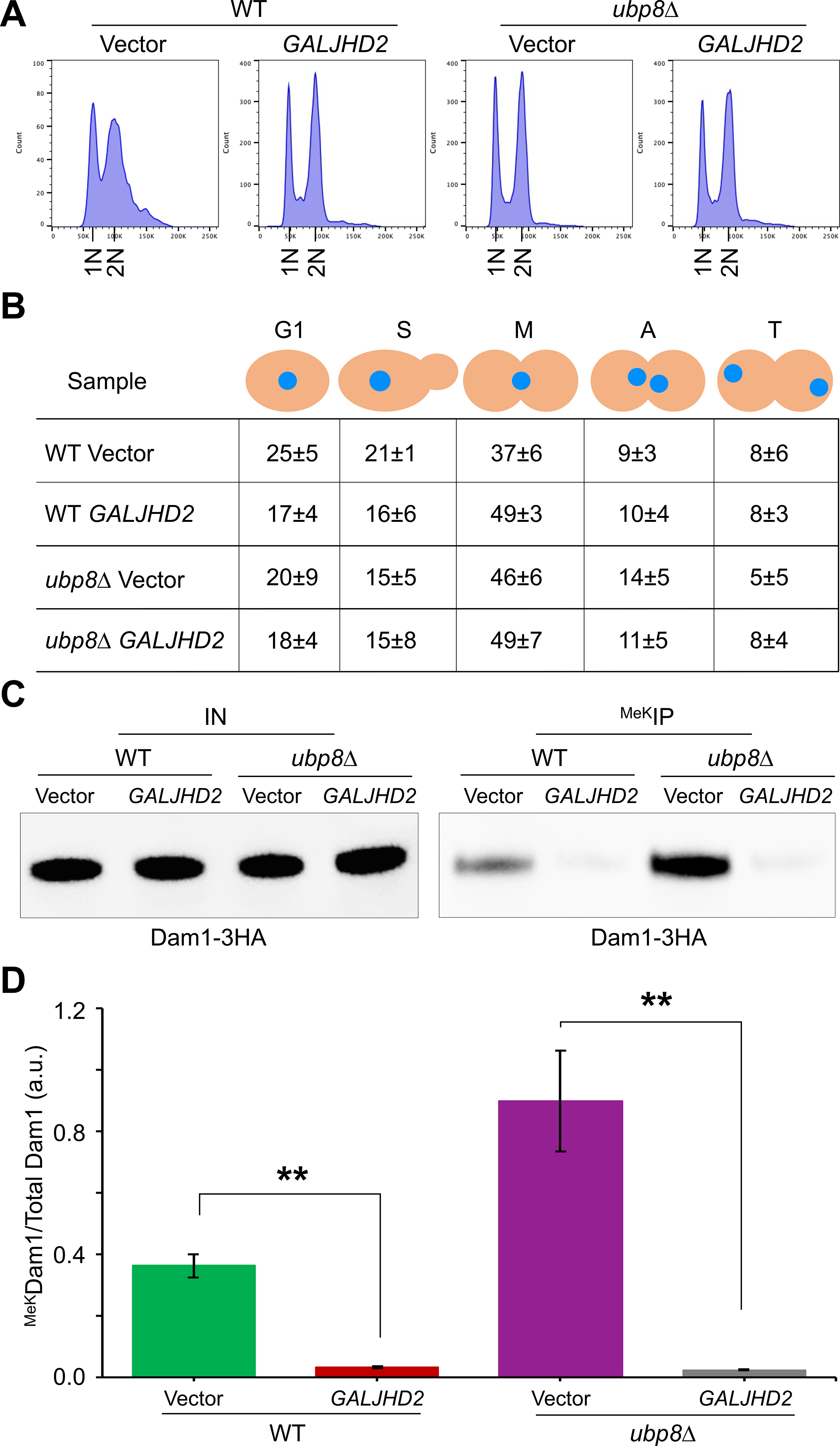

Supplement: S11 Fig — Wild type strain carrying pGAL1-URA3 vector (strain# YMB12844) or pGAL1-FLAG-JHD2-URA3 (strain# YMB12845) and ubp8△ strain carrying pGAL1-URA3 vector (strain# YMB12846) or pGAL1-FLAG-JHD2-URA3 (strain# YMB12847) were grown at 25°C to LOG phase in 1xSC-Ura media with galactose+raffinose (2% each) for 3 hours. All strains had HA-tagged Dam1 (Dam1–3HA) expressed from its endogenous promoter. (A) Flow cytometry showing DNA content. (B) Cell cycle stages of samples from (A) determined as described in Fig 2B. Different stages: G1, S-phase (S), metaphase (M), anaphase (A), and telophase (T). Average ± SD from three biological replicates is shown. (C) Overexpression of JHD2 results in reduced levels of MeKDam1 in ubp8△ strain. Methylated proteins were enriched by IP with anti-methyl lysine antibodies using whole cell extracts from (A) and analyzed by western blotting using α-HA (Dam1–3HA) antibodies. IN, input; MeKIP, immunoprecipitated samples. (D) Relative enrichment of MeKDam1. Enrichment was determined as described in Fig 1D. Three biological replicates were done. Average ± SE is shown. **p value <0.01, Student’s t-test. (TIF) [file pgen.1011760.s011.tif]

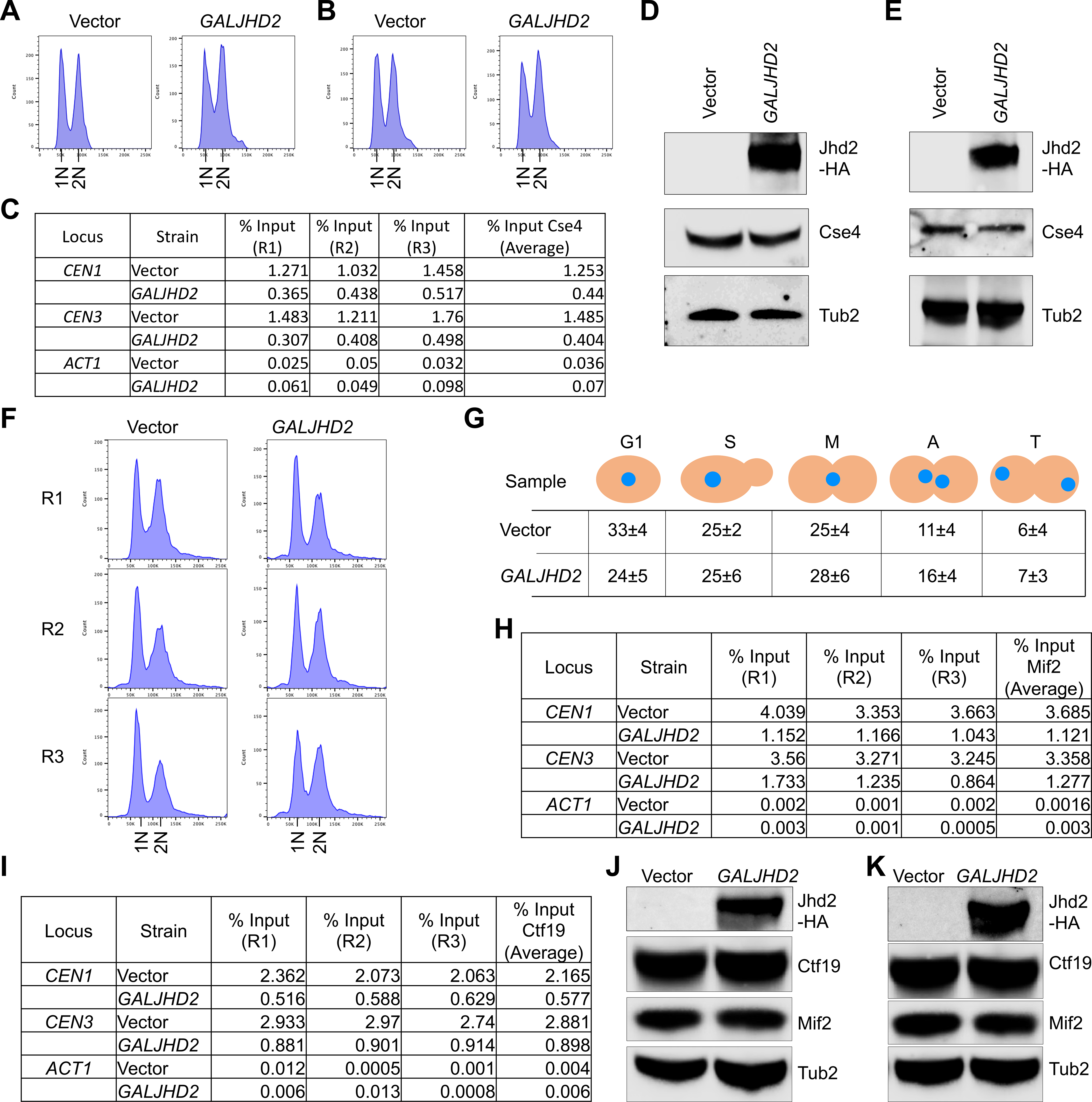

Supplement: S12 Fig — Additional biological replicates (Related to Fig 9). (A and B) Flow cytometry analysis representing DNA content. (C) ChIP-qPCR data showing CEN enrichment of Cse4. (D and E) Western blots showing protein expression of Cse4. (F) Flow cytometry analysis representing DNA content of samples used in ChIP-qPCR of Mif2 and Ctf19. (G) Cell cycle stages of samples used in ChIP-qPCR of Mif2 and Ctf19. (H) ChIP-qPCR data showing CEN enrichment of Mif2. (I) ChIP-qPCR data showing CEN enrichment of Ctf19. (J and K) Western blots showing protein expression of Mif2 and Ctf19. (TIF) [file pgen.1011760.s012.tif]

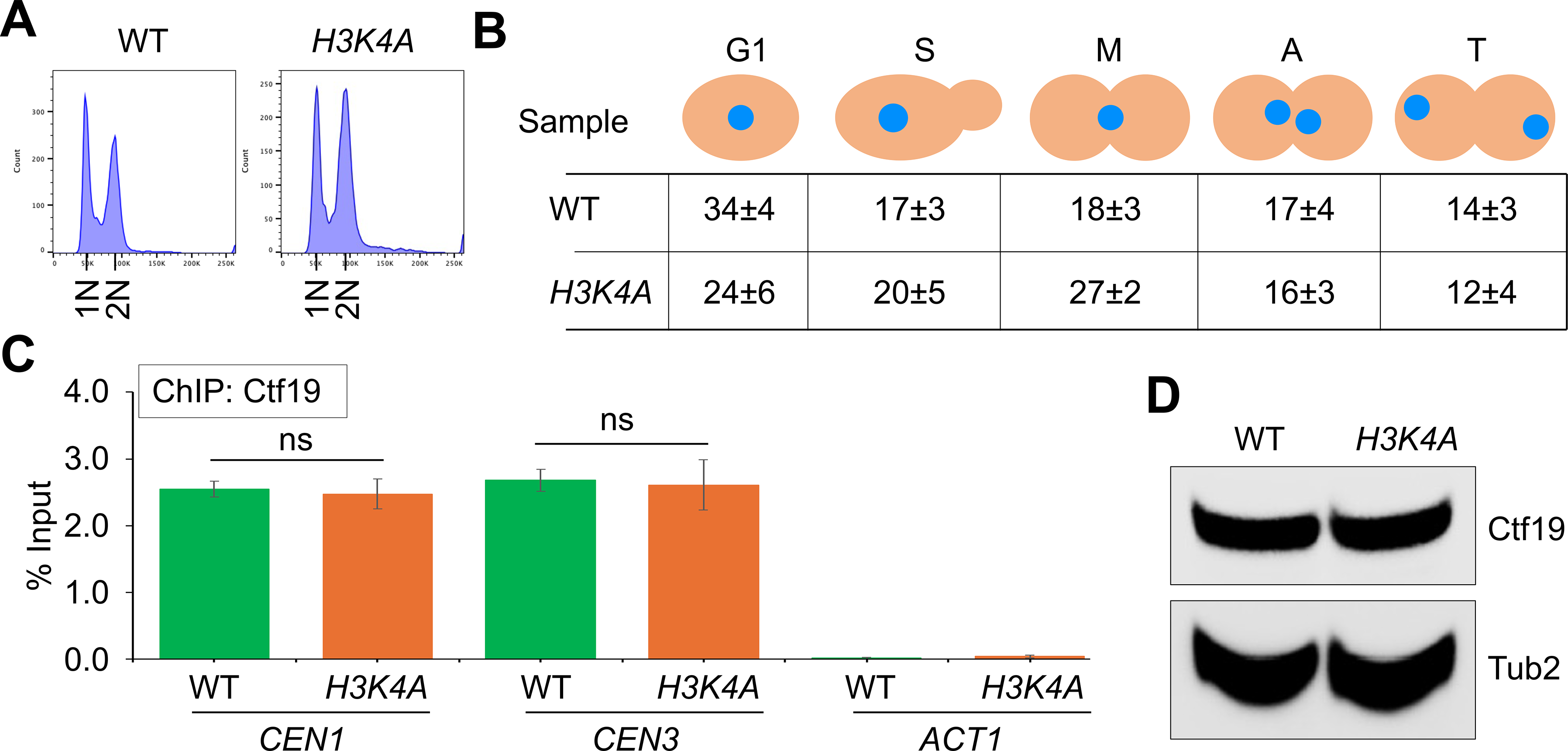

Supplement: S13 Fig — Wild type (strain# WT-12380) and histone H3K4A (strain# H3K4A-12382) strains were grown at 25°C to LOG phase in YPD for ChIP experiments. (A) Flow cytometry analysis showing DNA content. (B) Cell cycle stages of samples from (A) were determined as described in Fig 2B. Cell cycle stages are: G1, S-phase (S), metaphase (M), anaphase (A), and telophase (T). Average ± SD from three biological replicates is shown. (C) Levels of Ctf19 are not affected at CEN chromatin in H3K4A strains. ChIP was performed with α-Ctf19 antibodies using chromatin from strains in (A). Enrichment of Ctf19 at CENs (CEN1 and CEN3) and a negative control (ACT1) was determined by qPCR and is presented as % input. Average from three biological replicates ±SE. ns = not significant, Student’s t-test. (D) Expression of Ctf19 in wild type, and H3K4A strains are similar. The western blotting of protein extracts was done using α-Ctf19 and α-Tub2 (loading control) antibodies. (TIF) [file pgen.1011760.s013.tif]

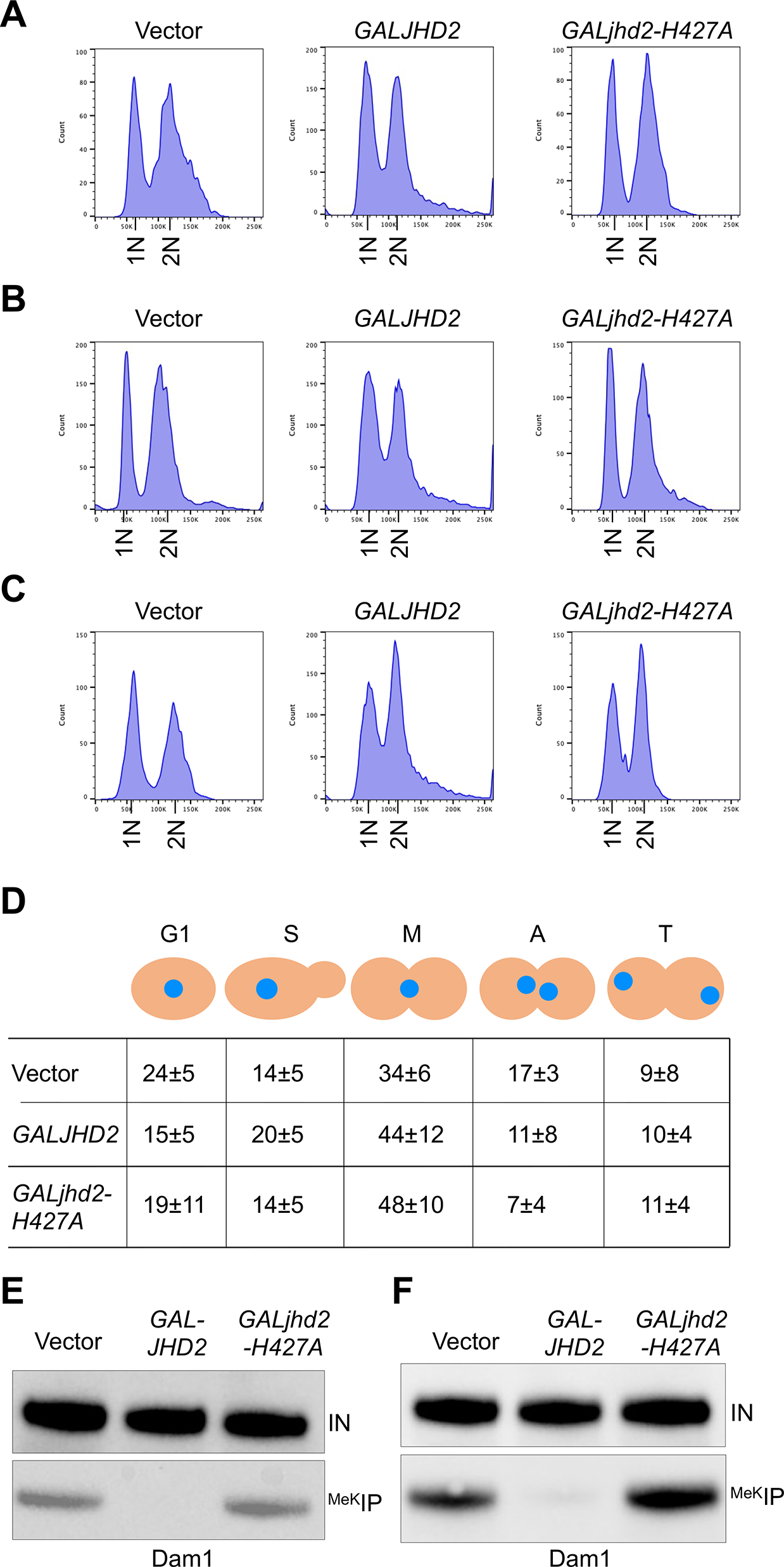

Supplement: S14 Fig — Additional biological replicates (Related to Fig 10E and 10F). (A, B and C) Flow cytometry showing DNA content. (D) Cell cycle stages of samples from (A) were determined as described in Fig 2B. G1, S-phase (S), metaphase (M), anaphase (A), and telophase (T) are shown as average ± SD from three biological replicates. (E and F) Overexpression of JHD2 results in reduction in the levels of MeKDam1 but not its catalytic mutant jhd2-H427A. Methylated proteins were immunoprecipitated with methyl lysine antibodies and analysed by western blotting using α-HA (Dam1–3HA) antibodies. IN, input; MeKIP, immunoprecipitated samples. (TIF) [file pgen.1011760.s014.tif]
