# Supplementary material for: Cell cycle dependent methylation of Dam1 contributes to kinetochore integrity and faithful chromosome segregation
Source: PLoS Genet. 2025 Jun 16;21(6):e1011760. doi: 10.1371/journal.pgen.1011760 (PMC12204631; doi:10.1371/journal.pgen.1011760)
Supplement: S2 Table — Chromosome loss rate data from different biological replicates (Related to Fig 10). (A) Overexpression of JHD2 causes errors in chromosome segregation. (B) GALJHD2-induced chromosome loss is mediated by its catalytic activity. (DOCX) [file pgen.1011760.s016.docx]

**S2 Table.**

**Overexpression of *JHD2* leads to CIN.** Chromosome loss rate data from different biological replicates (Related to Fig 10). (A) Overexpression of *JHD2* causes errors in chromosome segregation. (B) *GALJHD2-*induced chromosome loss is mediated by its catalytic activity.

**
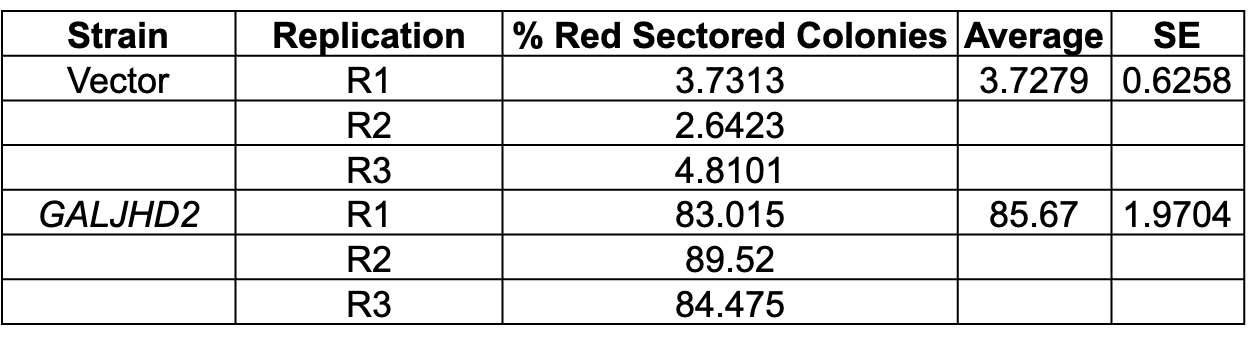
A**

**
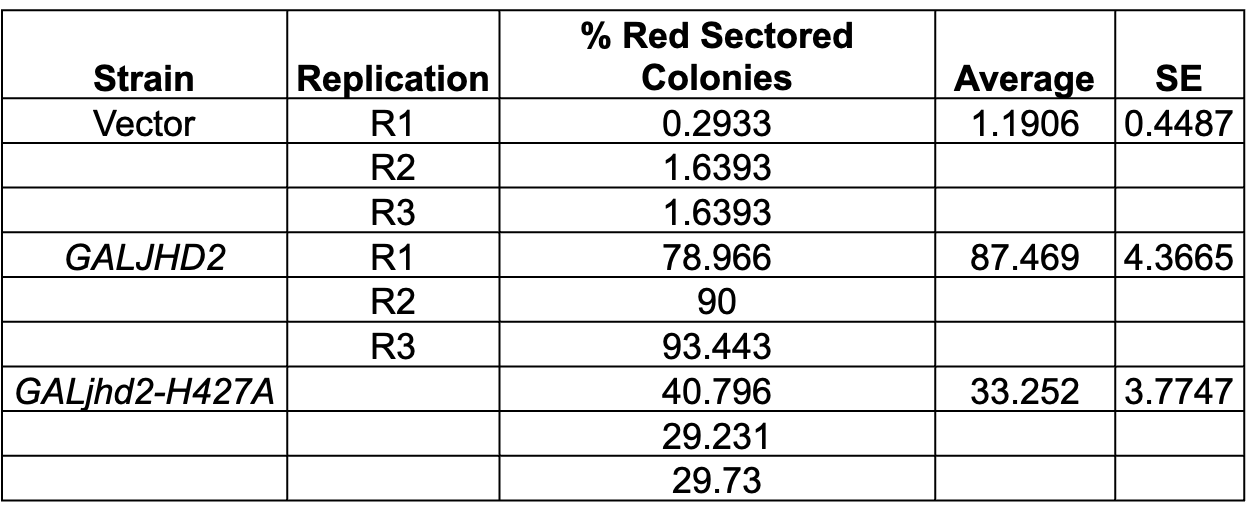
B**
