# Supplementary material for: Cell cycle dependent methylation of Dam1 contributes to kinetochore integrity and faithful chromosome segregation
Source: PLoS Genet. 2025 Jun 16;21(6):e1011760. doi: 10.1371/journal.pgen.1011760 (PMC12204631; doi:10.1371/journal.pgen.1011760)
Supplement: S1 Datas — (PDF) [file pgen.1011760.s017.pdf]

Supporting Information:

Supplemental Dataset - Mishra et al.

Western blots and raw experimental data from the study.
(labels are shown in corresponding figures in the manuscript).

Figure 1C

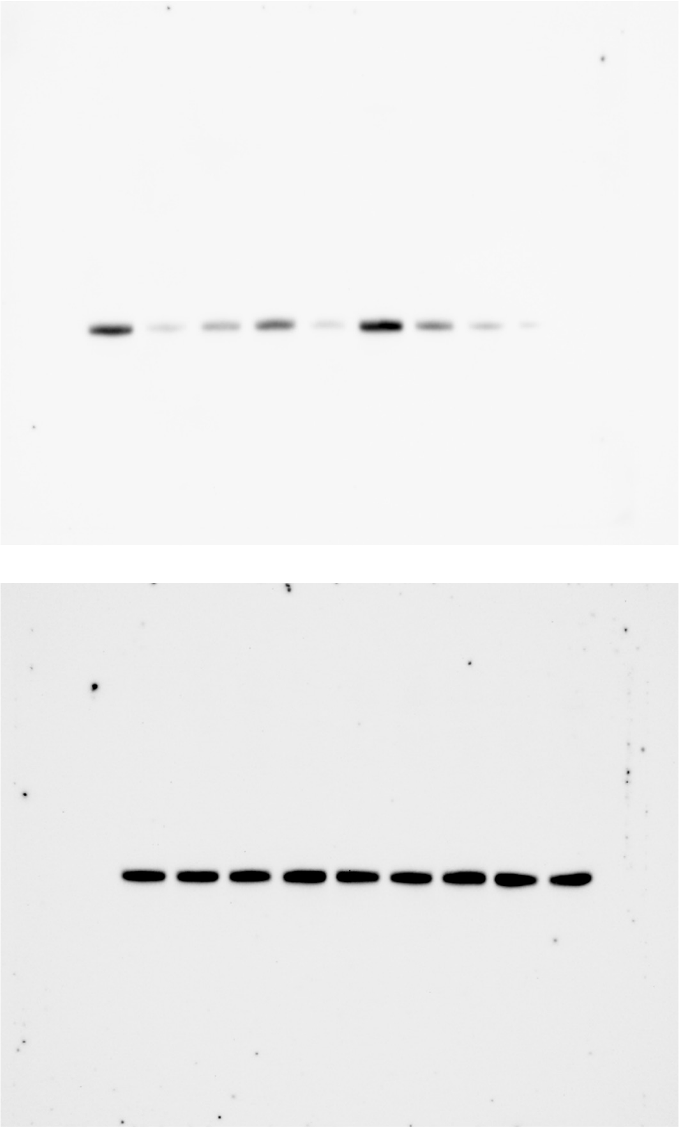

Figure 2C

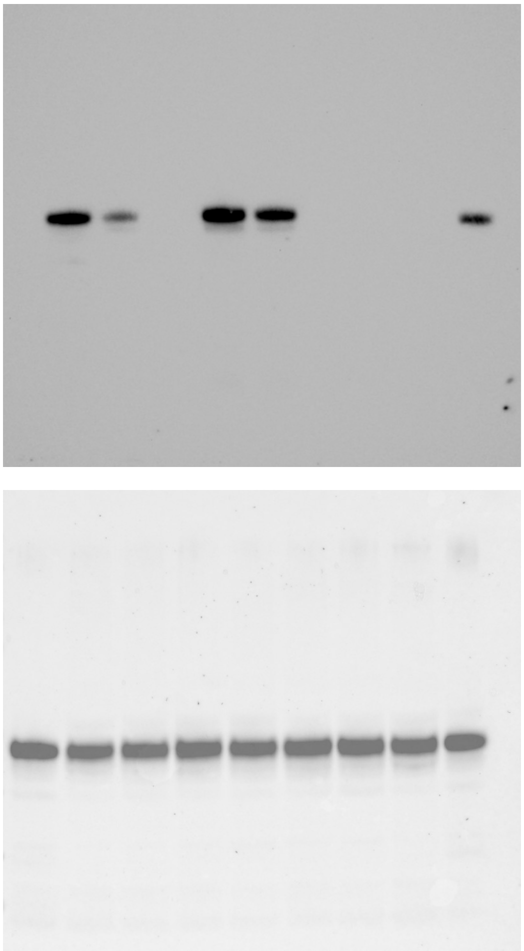

Figure 3C

G1 (α-factor) S (HU) Metaphase

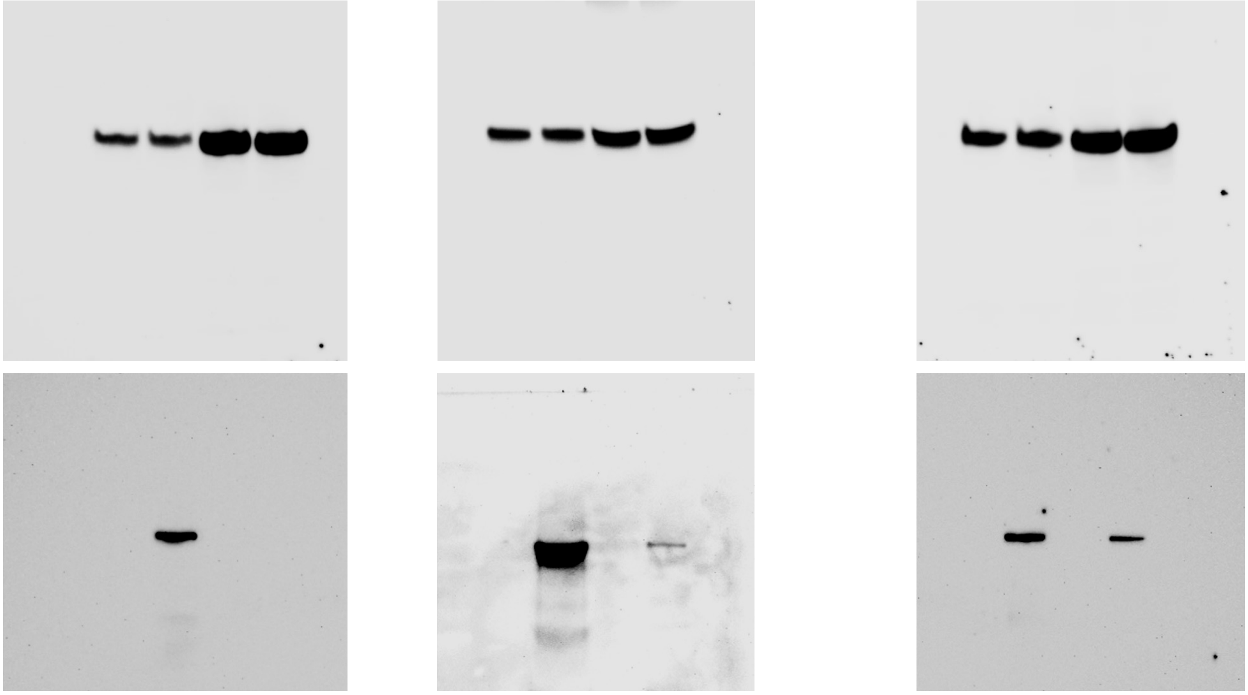

Figure 4C

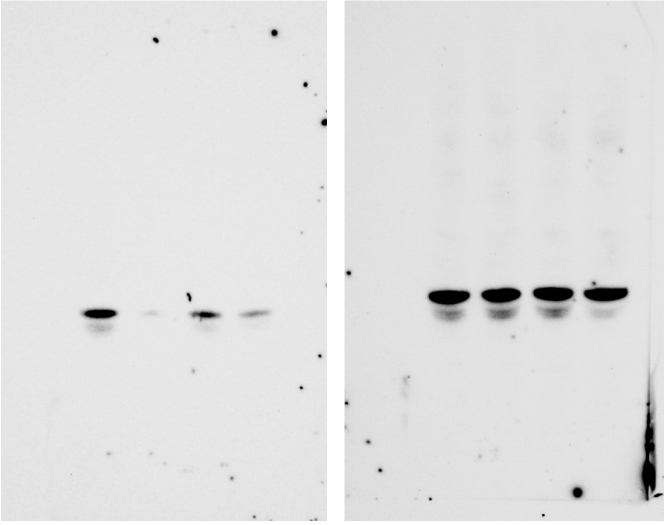

Figure 5C

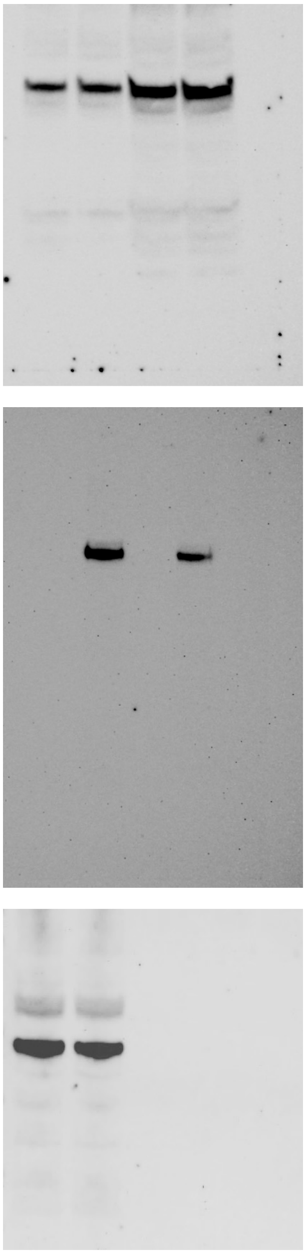

Figure 5D

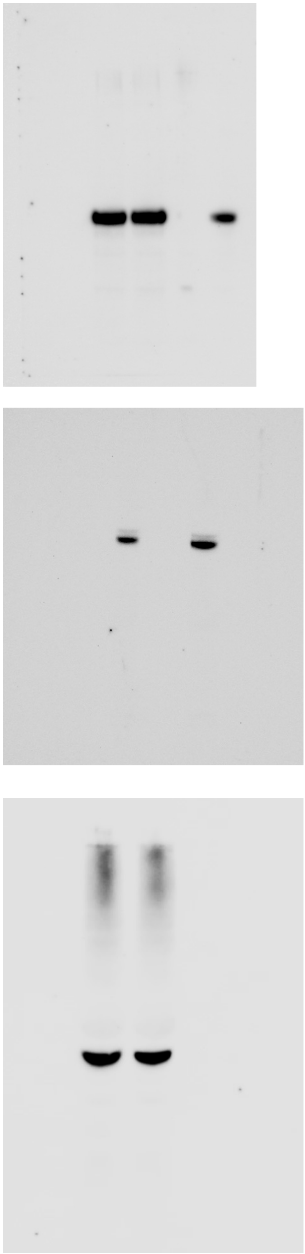

Figure 6C

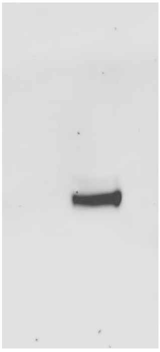

Figure 6D

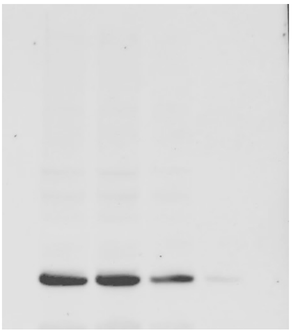

Figure 6F

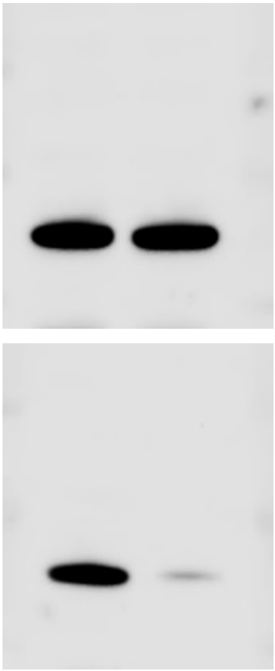

Figure 7C

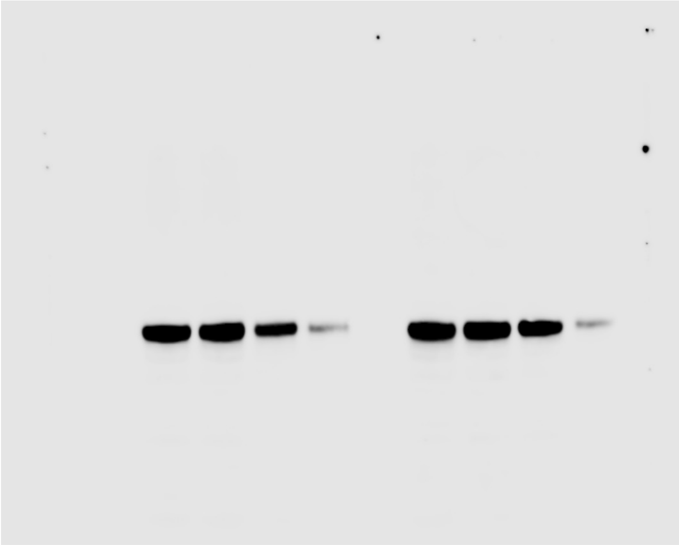

Figure 9F

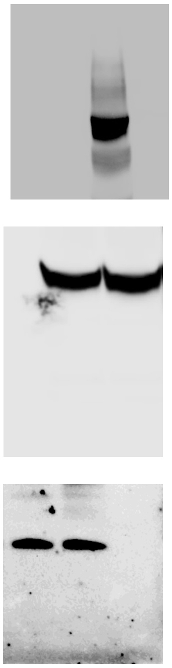

Figure 9G

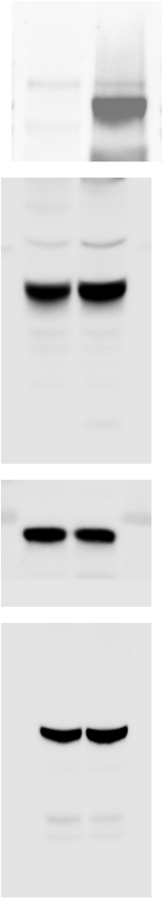

Figure 10E

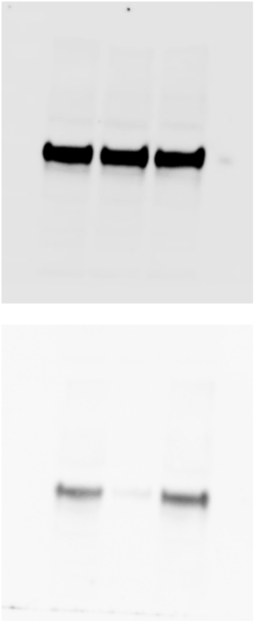

Figure S1

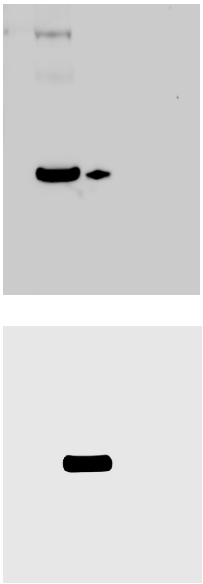

Figure S2

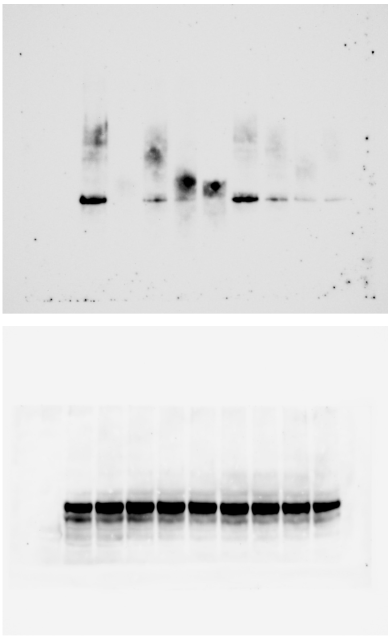

Figure S3

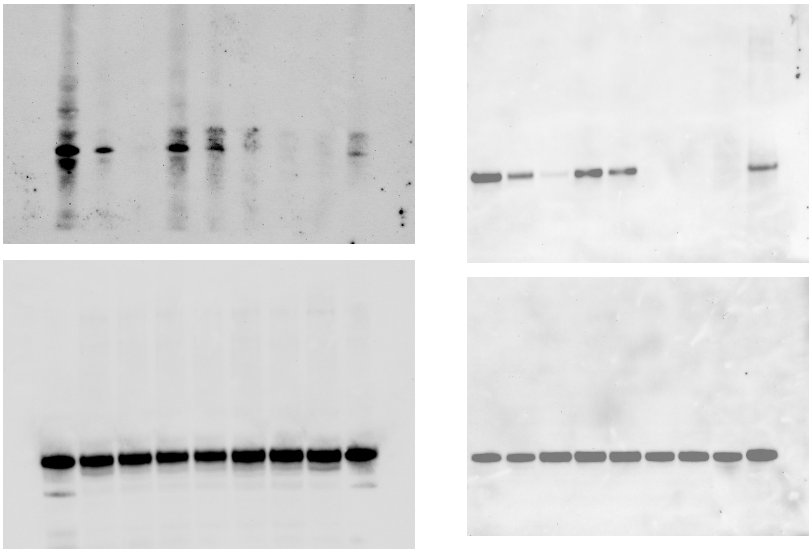

Figure S4B

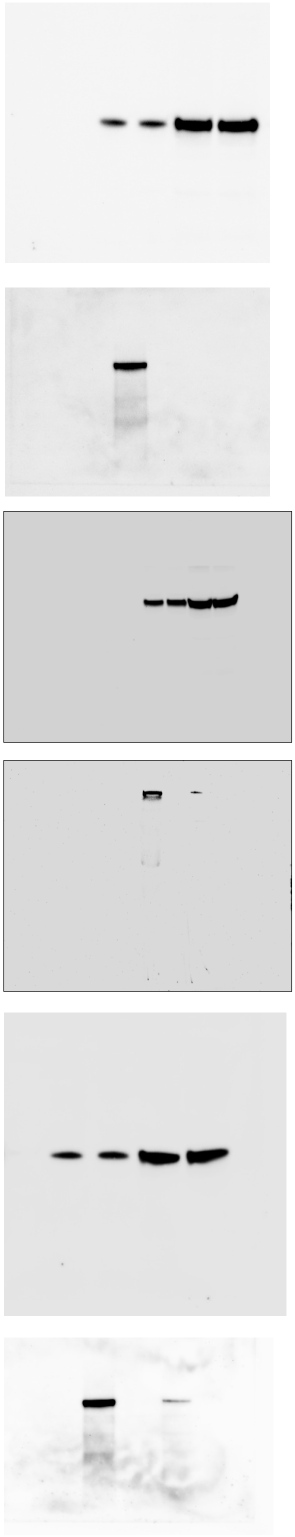

Figure S4D

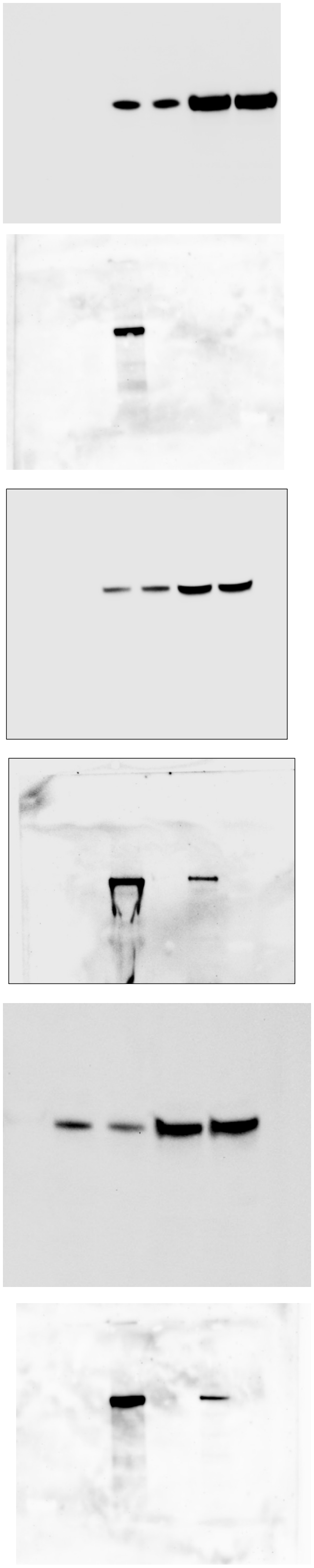

Figure S5B

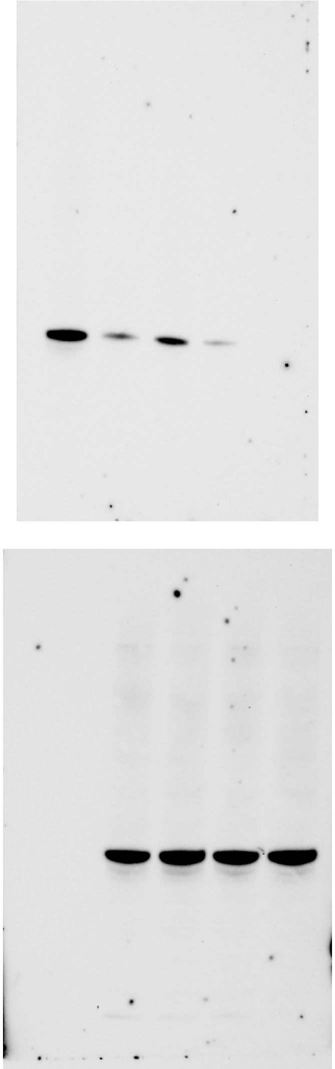

Figure S5D

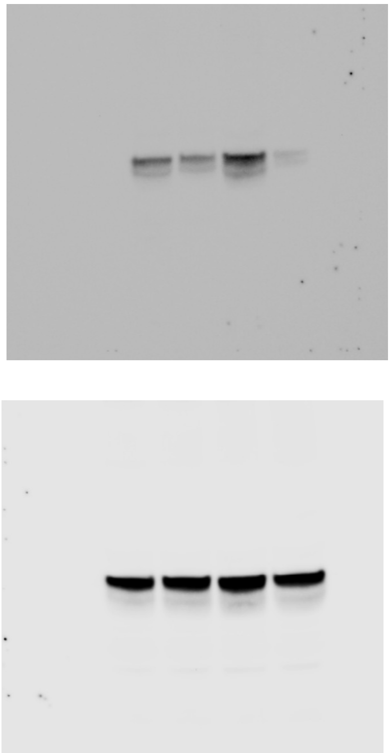

Figure S6C

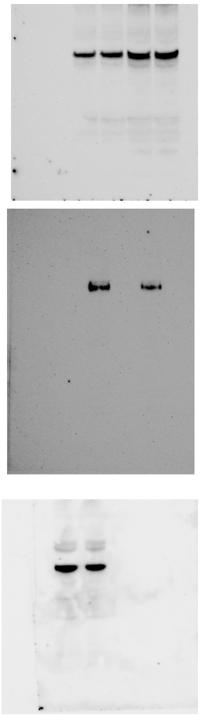

Figure S6D

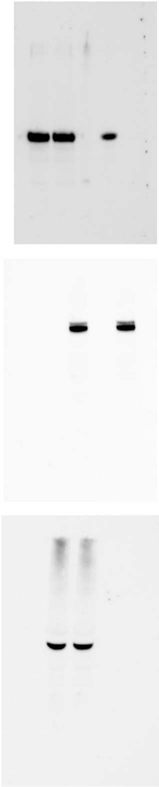

Figure S6E

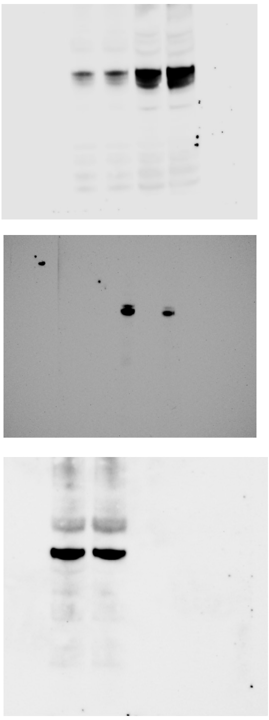

Figure S6F

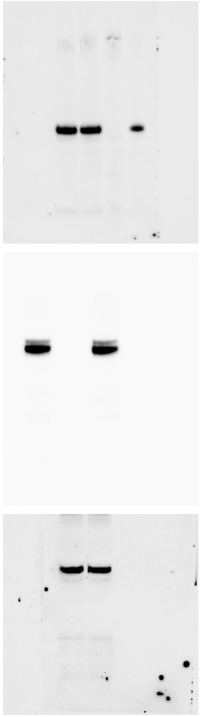

Figure S7E

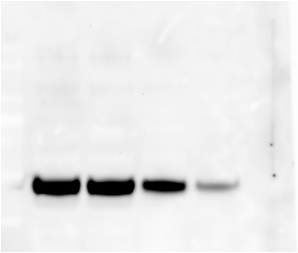

Figure S7G

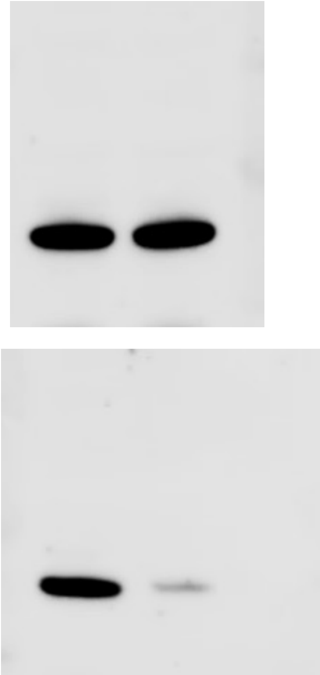

Figure S7C and D

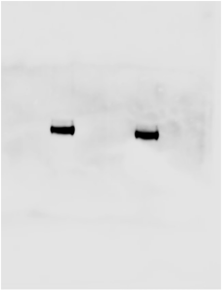

Figure S7F

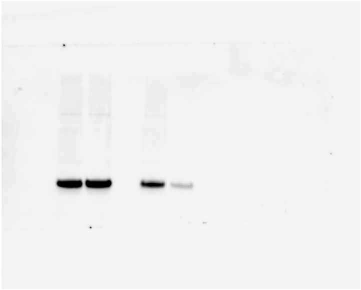

Figure S7H

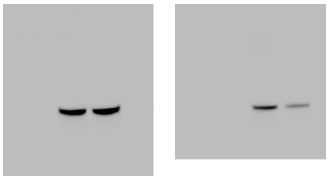

Figure S8C

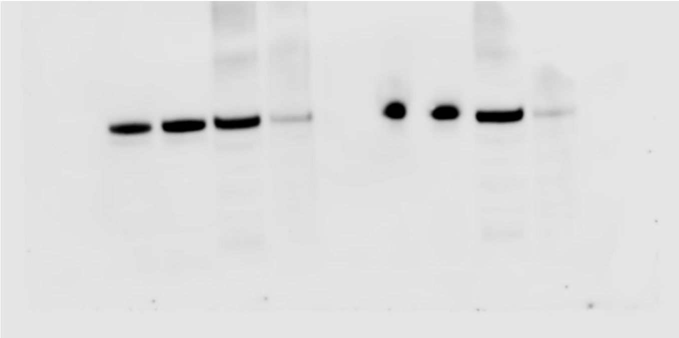

Figure S8D

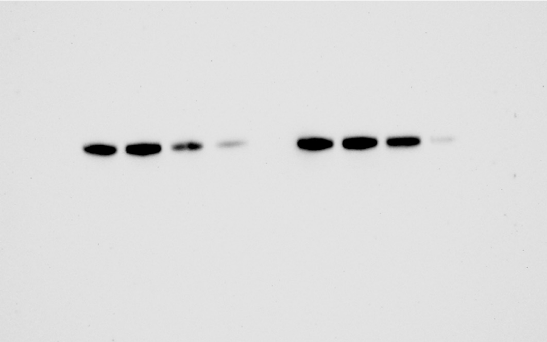

Figure S9C

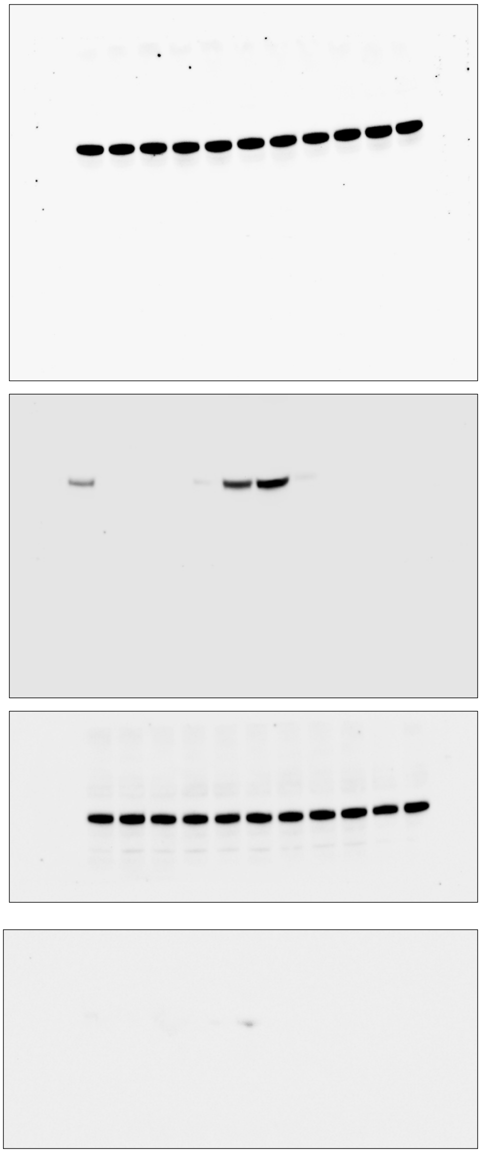

Additional Replicates
(related to Figure S9C)

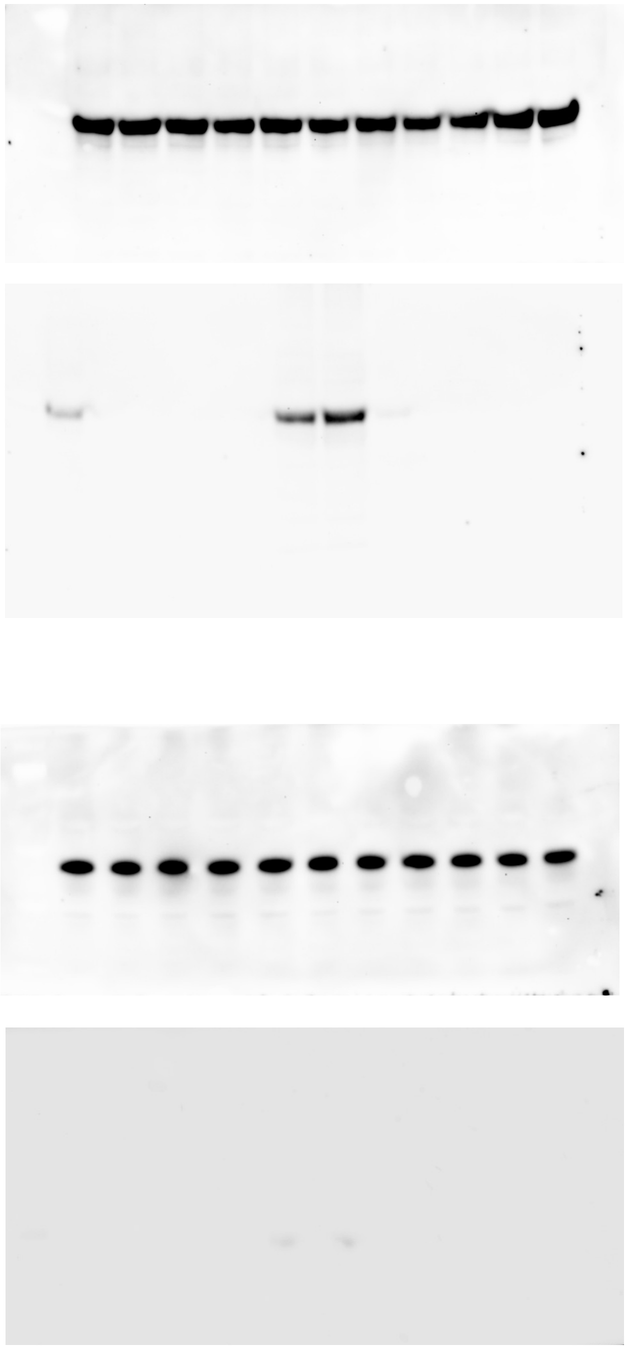

Additional Replicates
(related to Figure S9C)

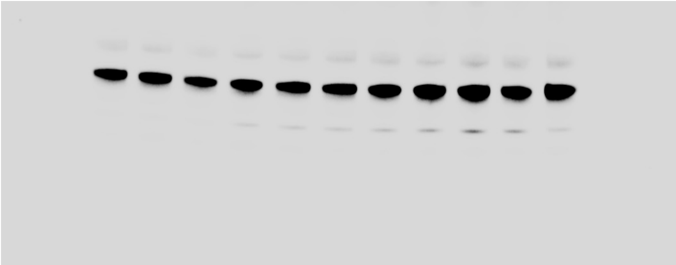

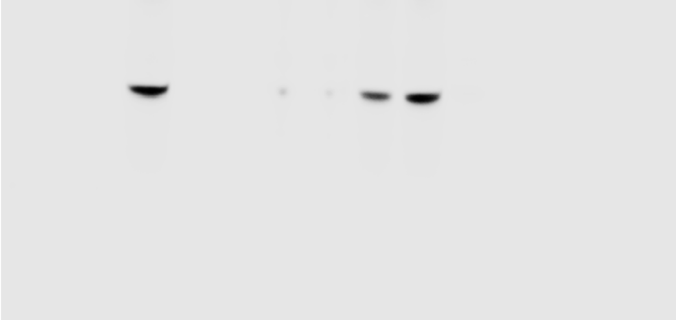

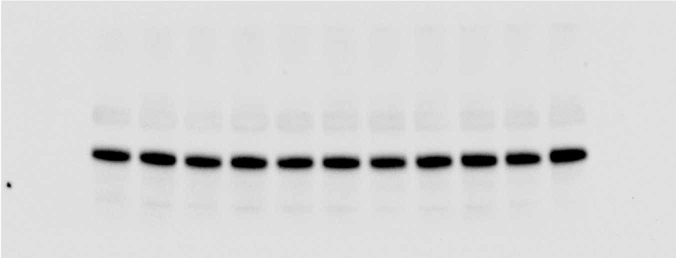

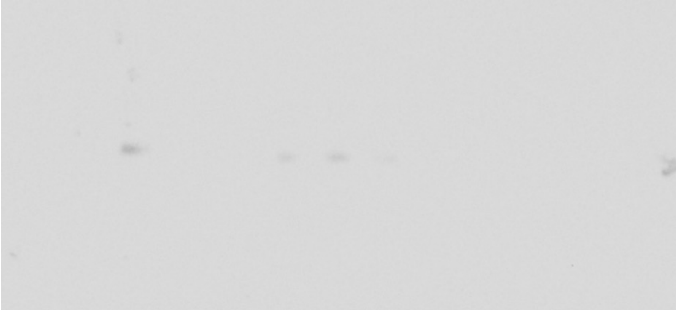

Figure S11C

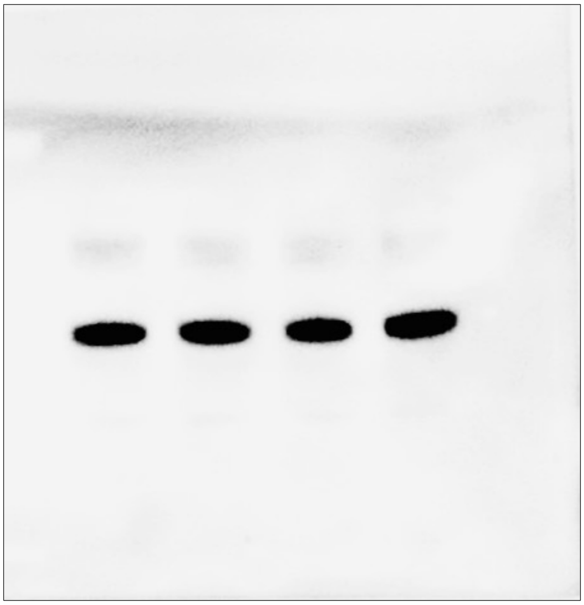

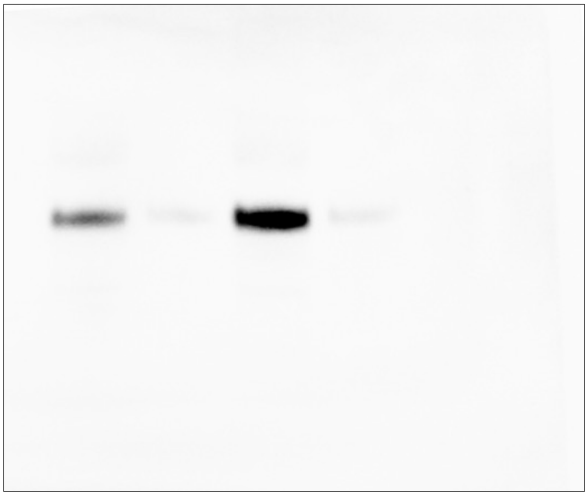

Figure S12D

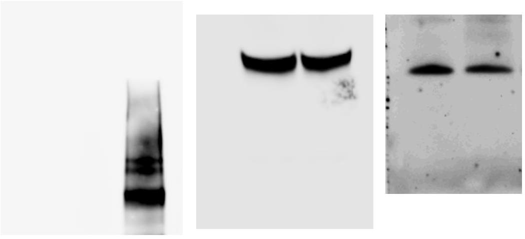

Figure S12J Figure S12K

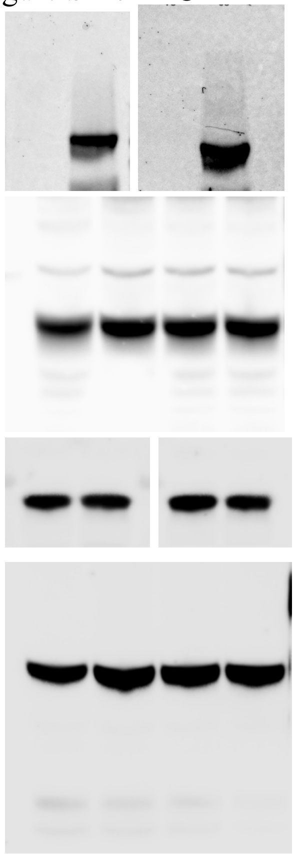

Figure S12E

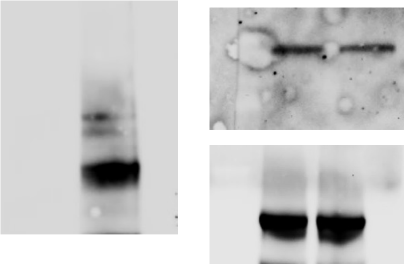

Figure S13D

Ctf19

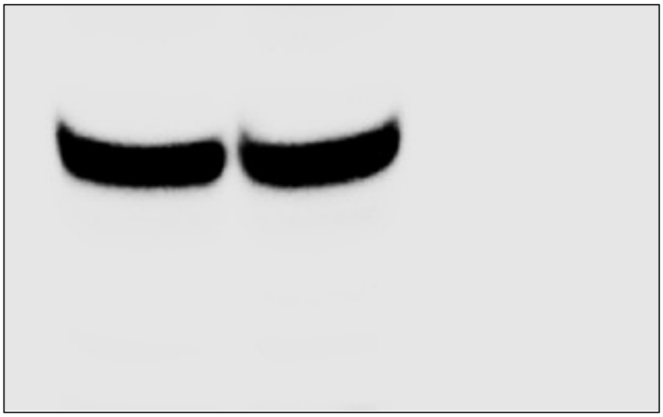

Tub2

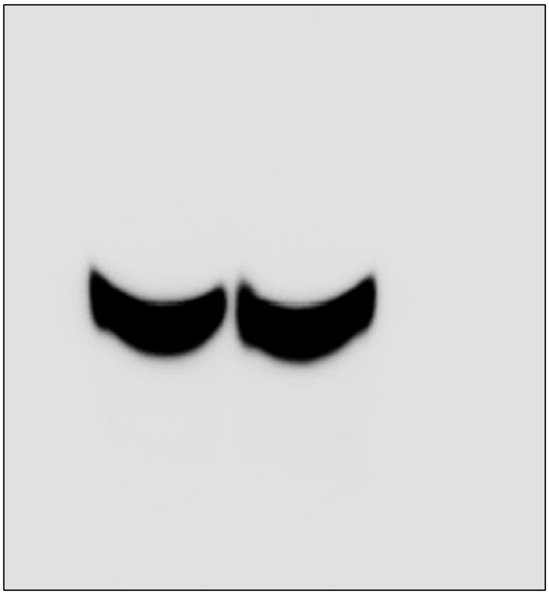

Additional Replicates
(related to Figure S13D)

Ctf19

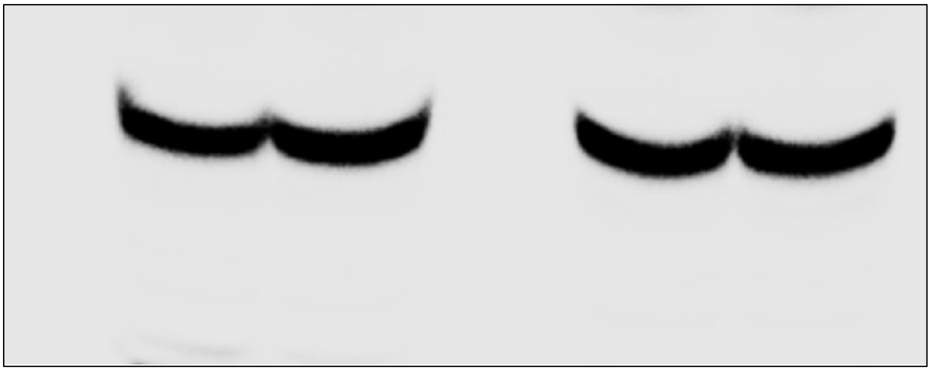

Tub2

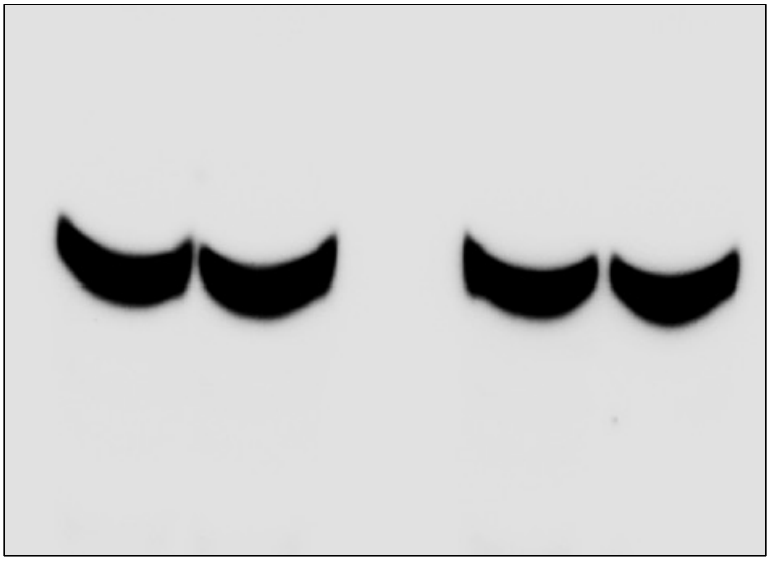

Figure S14E

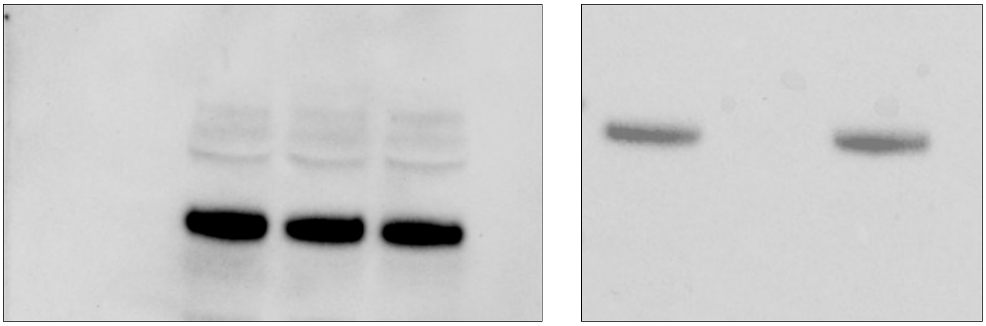

Figure S14F

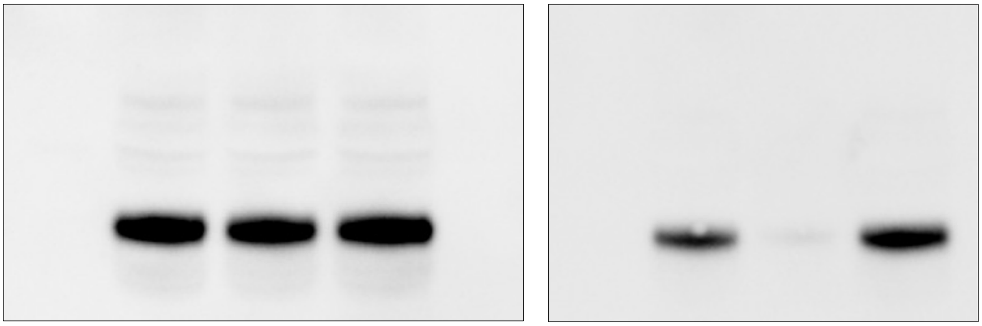

Figure 1D

Sample ID	R1 (meK-Dam1/Dam1)	R2 (meK-Dam1/Dam1)	Average (meK-Dam1/Dam1)	Range
LOG	0.92109883	0.59400633	0.75755258	0.3270925
0 min	0.00795567	0.04125073	0.0246032	0.03329506
20 min	0.13256138	0.12023428	0.12639783	0.0123271
40 min	0.07072926	0.25521173	0.1629705	0.18448246
60 min	0.04308197	0.0332545	0.03816824	0.00982747
80 min	0.54333608	0.82991316	0.68662462	0.28657708
100 min	0.10169473	0.15542153	0.12855813	0.0537268
120 min	0.02987159	0.03798079	0.03392619	0.0081092
140 min	0.02956951	0.0040053	0.0167874	0.02556422

Figure 2D

Sample ID	R1 (meK-Dam1/Dam1)	R2 (meK-Dam1/Dam1)	R3 (meK-Dam1/Dam1)	Average (meK-Dam1/Dam1)	SE
LOG	0.46643825	1.08834962	0.65716489	0.73731759	0.18394907
0	0.27855013	0.42083511	0.21518293	0.30485606	0.06080627
20	0.0188257	0.08672167	0.0634909	0.05634609	0.01992278
40	0.45702715	0.63652947	0.77519287	0.62291649	0.09209839
60	0.27309	0.54327877	0.52415559	0.44684145	0.08705094
80	0.10662084	0.05677326	0.02320166	0.06219859	0.02423335
100	0.01140996	0.07530494	0.02658608	0.03776699	0.01927349
120	0.02524973	0.14076398	0.0460263	0.07068	0.03555156
140	0.22648651	0.28932873	0.31802331	0.27794618	0.02703034

Figure 4D

Sample ID	R1 (meK-Dam1/Dam1)	R2 (meK-Dam1/Dam1)	R3 (meK-Dam1/Dam1)	Average (meK-Dam1/Dam1)	SE
LOG	0.59047933	1.19981538	0.70396772	0.83142081	0.18708806
G1	0.22278933	0.19607166	0.01724786	0.14536962	0.0645235
S	0.45858383	0.37083836	0.26530268	0.36490829	0.05587419
G2/M	0.06671415	0.05798551	0.09536523	0.07335496	0.01128991

Figure 6E

Sample ID	R1 (MeK-Dam1/Dam1)	R2 (MeK-Dam1/Dam1)	R3 (MeK-Dam1/Dam1)	Average (MeK-Dam1/Dam1)	SE
Vector	0.5848811	0.68111423	0.62310442	0.62969991	0.02797516
GALJHD2	0.08301742	0.21036912	0.12735075	0.14024576	0.03732437

Figure 6G

Sample ID	R1 (H3K4Me3/H3)	R2 (H3K4Me3/H3)	R3 (H3K4Me3/H3)	Average (H3K4Me3/H3)	SE
Vector	0.67858827	0.74840095	0.75147123	0.72615348	0.02379912
GALJHD2	0.0875706	0.13978788	0.37028121	0.19921323	0.08685208

Figure 7D

Sample ID	Treatment	R1 (MeK-Dam1/Dam1)	R2 (MeK-Dam1/Dam1)	R3 (MeK-Dam1/Dam1)	Average (MeK-Dam1/Dam1)	SE
LOG	Vector	1.17369795	0.74023962	0.49765923	0.8038656	0.19773154
	GALJHD2	0.15394881	0.09501184	0.08327141	0.11074402	0.02186664
Metaphase	Vector	1.63912026	0.73681741	0.68018031	1.01870599	0.3106377
	GALJHD2	0.13353946	0.02229039	0.05411009	0.06997998	0.0330806

Figure 10F

Sample ID	R1 (MeK-Dam1/Dam1)	R2 (MeK-Dam1/Dam1)	R3 (MeK-Dam1/Dam1)	Average (MeK-Dam1/Dam1)	SE
Vector	0.49373236	0.43164676	0.47651543	0.46729818	0.01850562
GALJHD2	0.01028035	0.0229105	0.03889551	0.02402879	0.00827939
GALJHD2-H427A	0.5364265	0.62082551	0.5151432	0.55746507	0.03227049

Figure S9D

Vector					
Sample ID	R1 (MeK-Dam1/Dam1)	R2 (MeK-Dam1/Dam1)	R3 (MeK-Dam1/Dam1)	Average (MeK-Dam1/Dam1)	SE
LOG	0.22047453	0.27157038	0.629106	0.37371697	0.12854359
0 min	0.00780549	0.0005486	0.0031108	0.00382163	0.00212482
30 m	0.00230376	0.00171562	0.01558172	0.0065337	0.00452719
60 m	0.00080368	0.00092909	0.00652811	0.00275362	0.00188759
90 m	0.01668167	0.0115891	0.02422158	0.01749745	0.00366942
120 m	0.62965896	0.39119426	0.33551662	0.45212328	0.09021121
150 m	1.07763644	0.55126221	0.80791355	0.81227073	0.15196677
180 m	0.03945129	0.02388898	0.00423866	0.02252631	0.01018782
210 m	0.00680744	0.00246869	0.00455053	0.00460889	0.00125283
240 m	0.00207667	0.00112781	0.00742431	0.00354293	0.00195992
270 m	0.00367199	0.00168334	0.01200351	0.00578628	0.00316118

GALJHD2					
Sample ID	R1 (MeK-Dam1/Dam1)	R2 (MeK-Dam1/Dam1)	R3 (MeK-Dam1/Dam1)	Average (MeK-Dam1/Dam1)	SE
LOG	0.08740136	0.10424706	0.11146859	0.101039	0.00713037
0 min	0.03896552	0.03795598	0.02120514	0.03270888	0.00575925
30 m	0.04417807	0.06627452	0.0149344	0.04179566	0.01486841
60 m	0.0297581	0.03863934	0.00979995	0.02606579	0.00852745
90 m	0.03881922	0.03556581	0.0988324	0.05773914	0.02056808
120 m	0.17789542	0.03811543	0.07117401	0.09572829	0.04217739
150 m	0.20595406	0.18363	0.12885002	0.17281136	0.0229059
180 m	0.06467371	0.01391762	0.04430591	0.04096575	0.01474689
210 m	0.03473287	0.00789589	0.00694128	0.01652335	0.00910893
240 m	0.03854152	0.0088305	0.00714446	0.01817216	0.0101963
270 m	0.05479273	0.01190334	0.01518176	0.02729261	0.01378259

Figure S11D

Sample ID	R1 (MeK-Dam1/Dam1)	R2 (MeK-Dam1/Dam1)	R3 (MeK-Dam1/Dam1)	Average (MeK-Dam1/Dam1)	SE
WT Vector	0.29686502	0.42317239	0.36864332	0.36289358	0.03657496
WT GALJHD2	0.03874831	0.02848534	0.03469626	0.03397664	0.00298443
ubp8-deletion vector	0.61500127	1.18410969	0.8993768	0.89949592	0.16428746
ubp8-deletion GALJHD2	0.02163543	0.02251006	0.02890713	0.02435088	0.00229208

Figure S13C

Locus	Strain	% Input (R1)	% Input (R1)	% Input (R1)	% Input Ctf19 (average)
CEN1	WT	2.39305937	2.4671684	2.77763338	2.54595372
	H3K4A	2.91168884	2.1702424	2.34706239	2.47633121
CEN3	WT	3.01019342	2.47401833	2.5612644	2.68182538
	H3K4A	2.50160878	3.31464919	2.01790211	2.61138669
ACT1	WT	0.00240101	0.00889282	0.03728802	0.01619395
	H3K4A	0.07074913	0.05547018	0.00442795	0.04354909
